# Photochemical Transformations of Diverse Biologically Active Resveratrol Analogs in Batch and Flow Reactors

**DOI:** 10.3390/molecules29010201

**Published:** 2023-12-29

**Authors:** Milena Mlakić, Hana Perinić, Vitomir Vušak, Ottó Horváth, Diego Sampedro, Raúl Losantos, Ilijana Odak, Irena Škorić

**Affiliations:** 1 Department of Organic Chemistry, Faculty of Chemical Engineering and Technology, University of Zagreb, Marulićev trg 19, HR-10 000 Zagreb, Croatia; mdragojev@fkit.unizg.hr (M.M.); hperinic@fkit.hr (H.P.); 2 Pliva R&D, Teva Pharmaceutical Industries Ltd., Prilaz baruna Filipovića 29, HR-10 000 Zagreb, Croatia; 3 Environmental and Inorganic Photochemistry Research Group, Center for Natural Sciences, Faculty of Engineering, University of Pannonia, P.O. Box 1158, H-8210 Veszprém, Hungary; horvath.otto@mk.uni-pannon.hu; 4 Departamento de Química, Centro de Investigación en Síntesis Química (CISQ), Universidad de La Rioja, 26006 Logroño, Spain; diego.sampedro@unirioja.es (D.S.); raul.losantosc@unirioja.es (R.L.); 5 Department of Chemistry, Faculty of Science and Education, University of Mostar, Matice Hrvatske bb, 88000 Mostar, Bosnia and Herzegovina; ilijana.odak@fpmoz.sum.ba

**Keywords:** computational study, flow photochemistry, hydroxy stilbenes

## Abstract

Previous biological tests have shown that some resveratrol analogs exhibited significant antioxidative and cholinesterase inhibitory potential, as evidenced by lower IC_50_ values compared to the established standards, resveratrol and galantamine, respectively. Photochemical transformations were made in parallel on these compounds in the presence of porphyrin photocatalysts in batch and microreactor, showing the significant advantage of flow photochemistry concerning productivity, selectivity, and yields. In this research, the products of photocatalysis and direct irradiation (photolysis) of resveratrol analogs were compared to elucidate how the types and ratios of the products depend on the excitation energy, to reveal the effects of the substituent on the photoinduced reactions and to rationalize experimentally and computationally the nature and ratio of the obtained products. Thus, two main paths were computed in agreement with the experimental results: isomerization with the participation of triplet state intermediates to yield the experimentally detected *cis*-isomers and subsequent cyclization following a pathway not available for the *trans*-isomers. The investigation of five model compounds confirmed the advantages of the flow photoreactor in the photochemical reactions of heterocyclic resveratrol analogs.

## 1. Introduction

**Figure 1:**
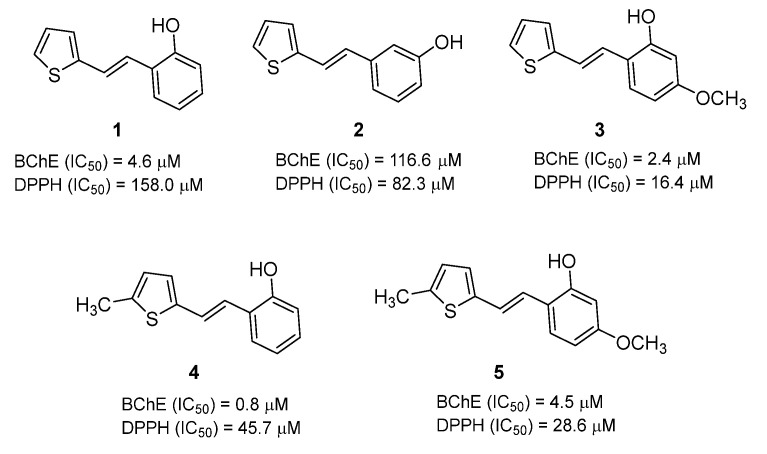
Structures of the investigated biologically active resveratrol derivatives **1**–**5**.

In our previous research [1], new analogs of the well-known bioactive molecule resveratrol were synthesized to investigate their potential biological activity. The configurational isomers of those derivatives underwent evaluation for antioxidant activity (AOA) using DPPH and CUPRAC assay, and their potential to inhibit acetylcholinesterase (AChE) and butyrylcholinesterase (BChE) was also measured. The biological tests have shown that the same compounds with *trans*-configuration (**1**–**5**, Figure 1) exhibited significant antioxidative and cholinesterase inhibitory potential, as evidenced by lower IC_50_ values compared to the established standards, *trans*-resveratrol and galantamine, respectively. On the other hand, precisely for compound **1** (Figure 1), we examined its photochemical transformations in the presence of porphyrin photocatalysts [2] in batch and microreactor. It has been proven that the use of microflow reactors can better control the formation of the desired products than is possible in batch reactors. The starting thiophene analog of *trans*-resveratrol (compound **1**) was chosen as a representative of heterostilbenes with proven biological activity for the transformation studies of photocatalytic oxygenation using an anionic and a cationic free-base porphyrin as well as their Mn(III) complexes. From the preparative synthesis in a batch reactor, four photoproducts were isolated as results of isomerization, dimerization, cyclization, and oxygenation, respectively.

Porphyrins offer several advantageous features for the realization of various types of catalytic reactions. They have a special macrocyclic aromatic molecular structure with 18π electrons. Free-base porphyrins are rather rigid tetradentate ligands which can coordinate a metal ion in the core of the macrocycle. Both such complexes and the free bases possess special redox properties and, due to the large, conjugated bond system, very efficient absorption ability in the visible range. All of these features make them suitable to catalyze a wide variety of processes in both natural and artificial systems. In a considerable part of these porphyrin-catalyzed reactions, due to the favorable photophysical and -chemical behavior of these macrocyclic compounds, visible-light irradiation is applied to populate their excited states from which further thermal or photochemical reaction steps take place. Free-base porphyrins, for example, were used for photocatalytic hydrogen evolution [3] and functionalization of biomolecules [4]. Metalloporphyrins were applied, among others, for various solar energy conversion systems [5] such as CO_2_ reduction [6], H_2_ production [7], and dye-sensitized solar cells [8,9], as well as in water treatment [10] and for medical purposes [11]. In the latter case, the most frequently used technique is photodynamic therapy, where porphyrins are photosensitizers producing highly reactive singlet oxygen via collisional energy transfer (ET) from their long-lived triplet state. However, this state can also be quenched efficiently by species other than dissolved oxygen molecules. For instance, different reactions of organic molecules could be sensitized with metalloporphyrins, e.g., *cis*-*trans* isomerization [12]. Also, in our previous work [2], such a sensitized *trans*-*cis* isomerization of compound **1** was observed. Additionally, we have also demonstrated that further reactions (such as dimerization and cyclization) of the *cis*-isomer formed could be promoted by both free-base and metalloporphyrins. In our systems, water-soluble porphyrins were used in order to meet some requirements of green chemistry. In hydroxylation reactions, mostly the photogenerated singlet oxygen molecules play the role of the oxidizing agent. However, in the presence of manganese (III) porphyrins, very reactive Mn^IV^=O and Mn^V^=O species can promote oxidation reactions. These species are formed in the following photoinitiated reaction steps [13,14,15] (Equations (1)–(3)):

(P)Mn^III^OH + *hν* → (P)Mn^II^ + ^•^OH (1)

2 (P)Mn^II^ + O_2_ → 2 (P)Mn^IV^=O (2)

2 (P)Mn^IV^=O + H^+^ → (P)Mn^V^=O + (P)Mn^III^OH (3)

The (P)Mn^V^=O species have the highest oxidizing potential in these systems, but the (P)Mn^IV^=O complexes could play important roles in the photocatalytic asymmetric epoxidation of *trans*-stilbene [16].

Our previous results regarding the photocatalytic transformation of **1** motivated us to compare the products of this method and direct irradiation (photolysis) of this resveratrol analog to elucidate how the types and ratios of the products depend on the excitation energy. In addition, other resveratrol analogs were also involved in this study to reveal the effects of the substituent on the photoinduced reactions of these compounds. Accordingly, in this research, the comparison of direct illumination of the resveratrol analogs **1**–**5** (Figure 1) with photocatalytic reactions was examined to rationalize experimentally and computationally the nature and ratio of the obtained products. Additionally, the influence of the reactor design and type on the photochemical transformations was analyzed for compounds **1**–**5**. The investigation of five model compounds confirmed the advantages of the flow photoreactor in the photochemical reactions of heterocyclic resveratrol analogs.

## 2. Results and Discussion

### 2.1. Photochemical Transformations of Resveratrol Derivatives ***1**–**5*** in Batch Reactor

**Scheme 1:**
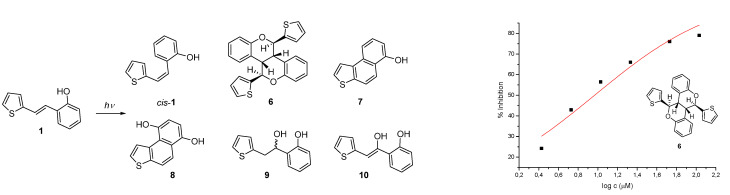
Direct irradiation (*λ* = 313/365 nm) of **1** or using free base and Mn(III) cationic/anionic porphyrins (*λ* = 365/405/420/450 nm) and dose-inhibition curve for inhibition of BChE by dimer **6** in a batch reactor.

Resveratrol analogs, hydroxy stilbenes **1**–**5**, were synthesized using the Wittig reaction according to a known procedure [1] as starting substrates for photochemical transformations. Compound **1** under direct illumination in a batch reactor showed primarily *trans*-*cis* isomerization and formation of *cis*-**1**, while in the presence of a photocatalyst, the reaction mixture was more complex (photoproducts **6**–**10**), but with prominent differences depending on the type of catalyst (Scheme 1). 

The synthesis and photochemistry of numerous heterostilbenes were thoroughly investigated in our research group in order to determine the biological properties of obtained compounds. Photocyclization products of stilbenes were usually more successful in inhibiting enzyme cholinesterases, particularly butyrylcholinesterase. Because of their relatively high photochemical stability, resveratrol analogs allowed us to investigate only on prepared stilbene skeleton, and they proved to be very strong inhibitors, especially compound **1** (IC_50_ = 15.7 μM for acetylcholinesterase and 4.6 μM for butyrylcholinesterase), a precursor for dimer **6**. Since photoproduct **6** was obtained and approved earlier by its crystal structure [2], it was interesting to check whether this dimeric product **6** is biologically active. Dimer **6** was screened for acetylcholinesterase (AChE) and butyrylcholinesterase (BChE) inhibition activity in a wide range of concentrations, and the results were reported as maximal inhibition of AChE and BChE at the maximal concentration tested and, if achieved, the IC_50_ value was calculated. Dimer **6** showed no activity toward AChE, while excellent inhibition was achieved for BChE in the micromolar range. IC_50_ value of 9.3 μM was in the same range as for standard reversible inhibitor galantamine (IC_50_ 7.9 μM for BChE). It can be concluded that dimer **6** is a selective, strong inhibitor of BChE (Table 1).

This fact provided justification for an even more detailed examination of the photochemical reactivity of compound **1** (but also of other analogs **2**–**5**, Figure 1) and finding reaction conditions under which either the corresponding *cis*-isomer of the starting compound or one of the photoproducts with potential biological activity can be selectively obtained. 

Table 2 summarizes the yields of photoproducts obtained from the starting compound **1** under direct irradiation, i.e., photolysis, compared to those achieved by photocatalysis using free-base anionic and cationic porphyrins and the corresponding complexes with manganese(III) metal center. Concerning starting compound **1**, conversions were very good in all cases. As the results of our previous study suggested [2], the primary photoinduced step in the photocatalytic transformation of starting compound **1** is *trans*-*cis* isomerization, probably facilitated by sensitization, i.e., via triplet-singlet energy transfer from a long-lived triplet excited state of the applied porphyrins to compound **1**. Not surprisingly, direct irradiations at higher energies than those used for the excitation of the porphyrin sensitizers also caused efficient isomerization of the starting compound. In the case of photolysis, irradiation was realized in the UV range at a higher and a lower energy (with the wavelengths of 313 and 365 nm, respectively). Although the conversion at 313 nm irradiation was lower than that at 365 nm excitation (80% vs. 100%), this difference originated from the lower photon flux caused by, the higher energy per photon ratio and partly by the lower efficiency of electric to light energy conversion at the shorter wavelength. However, the efficiencies of the further reactions of the *cis*-**1** isomer strongly deviate at the two applied irradiation wavelengths. Despite the higher conversion at 365 nm irradiation, the *cis*-**1** isomer accumulated (with a 91% ratio), i.e., it reacted further to a very limited extent, resulting in 7% of the electrocyclic product (**7**) and 2% of its hydroxylated derivative (**8**). These ratios also suggest that the formation of **8** is preceded by that of **7**. No other products were formed, which confirms that higher energy is needed for efficient further reactions of *cis*-**1**. In accordance with this conclusion, at higher-energy (313 nm) irradiation, the *cis*-**1** isomer accumulated to a lesser extent (52%, which would be 65% of the products, taking the 80% conversion into account), and also a considerable ratio of the dimer (**6**) (8% → 10%) and a double amount of product (**9**) hydroxylated at a carbon atom of the aliphatic part (16% → 20%). These data clearly indicate that the formations of **6** and **9** require higher excitation energy, i.e., the reactions leading to these products take place from a higher-energy excited state of *cis*-**1**, which can be populated by a 313 nm irradiation, but not by a 365 nm one. Also, upon 313 nm irradiation, the cyclization product (**7**) and its hydroxylated derivative (**8**) were formed, but with lower ratios (3% and 1%, respectively), indicating that the formation of **7** less efficiently takes place from the corresponding excited state of *cis*-**1** than those leading to the products **6** and **9**. Notably, deviating from the porphyrin-based photocatalysis where singlet oxygen can play an important role in hydroxylation, under direct irradiation (in the absence of photocatalyst), hydroxylation can only take place via interaction between the corresponding excited state of the organic molecule and the ground-state of the dissolved oxygen molecule.

**Figure 2:**
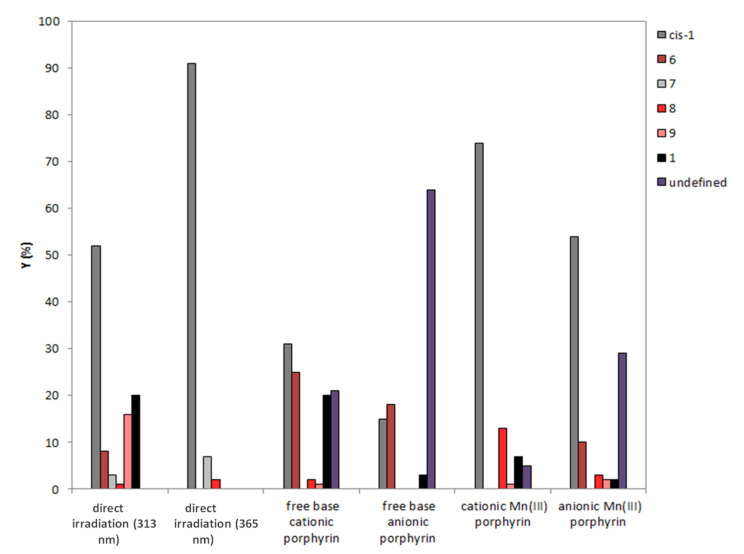
Yields (%) of photoproducts from **1** obtained using direct irradiation or Mn(III) and free base cationic/anionic porphyrins for reactions performed in a batch reactor (30 min, 2 h, and 6 h).

The results of the photocatalyses shown in Table 2 suggest some general tendencies. The free-base porphyrins promote the further reactions of *cis*-**1** much more efficiently than the corresponding Mn(III) complexes do, i.e., the accumulation of this isomer reached much higher ratios in the latter case, and, accordingly, the ratios of further products are much smaller. The free-base porphyrins facilitate the dimerization of *cis*-**1** and the subsequent polymerization processes more effectively than the corresponding metalloporphyrins do. These results may be accounted for by the different interactions between the *cis*-**1** isomer and the porphyrins. In the case of the free bases, π-π (stacking) interactions between the aromatic double-bond moieties of porphyrins and the *cis*-**1** isomer can connect several isomers to the same side of the macrocycle. In the case of the Mn(III) porphyrins, however, the metal center can coordinate only one molecule of *cis*-**1** due to steric limitations, but more strongly than π-π interactions do. Hence, the diminished number of coordinated isomers decreases the chance for dimerization and subsequent polymerization. Interestingly, the anionic porphyrins promote dimerization and polymerization much more effectively than the cationic ones do. The reason for this may be that the higher electron densities can result in stronger π-π interactions. In the case of photocatalysis, no cyclic product was detected because either it did not form at all, or it reacted further very efficiently. The latter possibility is demonstrated by the ratios of the hydroxylated derivative of the cyclic product in the presence of metalloporphyrins. The efficient hydroxylation can be the result of the reaction with singlet oxygen generated by these porphyrins as sensitizers, but in the case of Mn(III) porphyrins, also the oxidizing effects of the Mn(IV) and Mn(V) species can play considerable roles. In the latter case, the cationic metalloporphyrin is more efficient due to its stronger electron withdrawing effect. The described observations are shown for compound **1** also in Figure 2.

**Figure 3:**
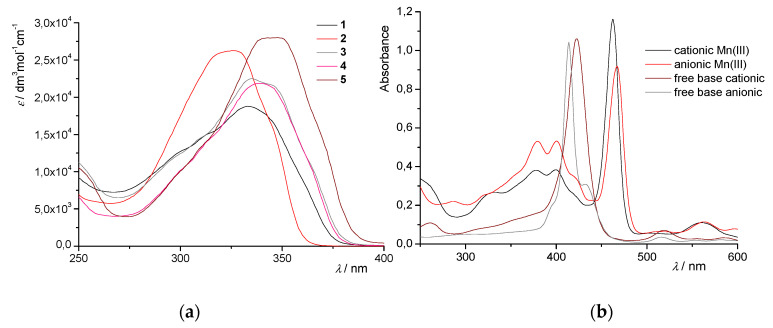
UV spectra (ACN) of the used (**a**) substrates **1**–**5** and (**b**) photocatalysts.

As it turned out from the above experiments performed on compound **1**, the most selective reaction in terms of *trans*-*cis* isomerization was the one under direct illumination (especially at 365 nm). Figure 3 shows parallel quantitative UV spectra of starting compounds **1**–**5** (a) and the applied photocatalysts (b). It can be seen that compounds **1**–**5** absorb radiation very well at the used wavelengths of 313 and 365 nm, while photocatalysts at longer wavelengths. At the same time, free bases absorb only in a narrow range at around 420 nm, while Mn(III) complexes have a maximum absorption at 450 nm but also absorb at wavelengths below 400 nm (Figure 3), which was reflected in the experimental results for photochemical transformations. UV-Vis spectra of **1**–**5** in ACN (Figure 3) are typical for diarylethenes [17,18] with strong absorption with a maximum of 335–340 nm.

**Scheme 2:**
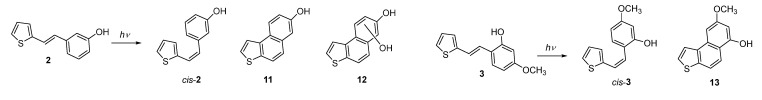
Direct irradiation (*λ* = 313/365 nm) of **2** and **3** in a batch reactor.

**Scheme 3:**
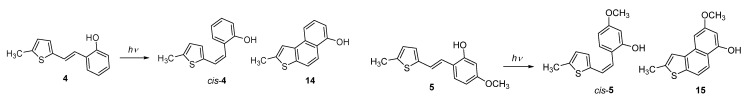
Direct irradiation (*λ* = 313/365 nm) of **4** and **5** in a batch reactor.

As reactions were cleaner without photocatalysts in the case of **1**, for resveratrol analogs **2**–**5,** only direct irradiations at 313 and 365 nm were conducted in a batch reactor (Scheme 2 and Scheme 3, Table 3). As can be seen, compounds **2**–**5** gave mainly the corresponding *cis*-isomer and its electrocyclization product under the applied conditions. Only in the case of compound **2** (the only one with a *meta*-positioned OH group) was the formation of the hydroxylated derivative **12** detected (obtained from the photoproduct **11**).

**Figure 4:**
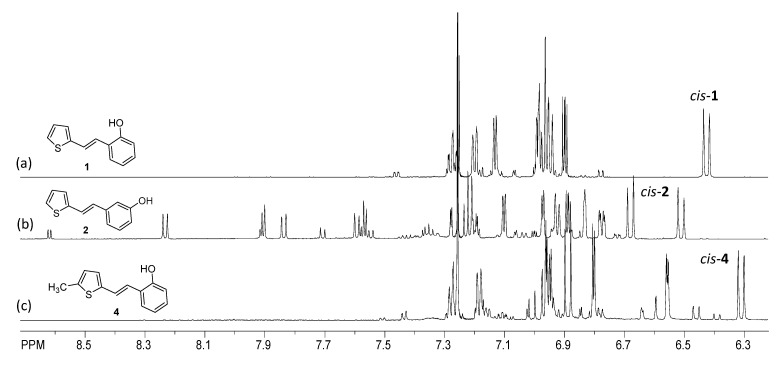
Parts of the NMR spectra of the photomixtures from **1** (**a**), **2** (**b**), and **4** (**c**) after 30 min of direct irradiation at 365 nm in a batch reactor.

Figure 4 shows the comparative ^1^H NMR spectra of the obtained photomixtures after 30 min of direct irradiation of derivatives **1**, **2**, and **4** at 365 nm in a batch reactor. In addition to the unreacted initial *trans*-isomers of compounds **1**, **2**, and **4**, doublets for protons of double bonds with characteristic coupling constants for *cis*-isomers are most clearly visible. In the previous research [2], photoproducts **6**–**10** obtained from compound **1** were separated by column chromatography, and chromatograms were recorded for them in which all their retention times were detected, so they were easily identified in this new experiment. Also, the pure *trans*- and *cis*-isomers of compounds **2**–**5** were also confirmed by NMR, MS, and HPLC techniques (Supplementary Materials), so their photoproducts **11**–**15** were relatively easy to identify, taking into account the comparison of their retention times with the same type of photoproducts obtained by illuminating compound **1**.

Table 3 summarizes the conversion data and yields of photoproducts obtained from the starting compounds **1**–**5** under direct irradiations at two different wavelengths (313 and 365 nm) in a batch reactor. The conversion data indicate relatively high efficiencies of the photoinduced transformations of all starting compounds except for **3**. Since, in the case of all starting compounds, the predominant product is the corresponding *cis*-isomer, the relatively low conversion data of **3** indicate that its photoinduced isomerization is hampered by at least one factor, which is negligible or of minor significance in the transformation of the other compounds. One may suspect the sterical hindrance because of the two substituents on the benzene ring. This situation, however, also occurs in the case of compound **5**, in the case of which rather high conversions were reached, moreover, significant ratios of the cyclic product formed. These results may suggest the electronic role of the substituents. While both substituents on the benzene ring are electron withdrawing, the methyl group on the thiophene is an electron donating one. Hence, the latter one, via the double bond, can compensate hindering effect of the electron withdrawing groups. The various substituents may influence the properties of the excited states populated by the 313 and 365 nm irradiations, therefore affecting the *trans*-isomer formation efficiency. Dimerization is not favored for any of the starting compounds except for **1** (even in that case only with higher-energy irradiation, as discussed previously). These results suggest the positions of the substituents in compounds **2**–**4** are not favorable for dimer formation. Cyclization relatively efficiently took place in most cases, reasonably from the *cis*-isomer. Only irradiations of compounds **3** and **4** did not produce cyclic products, probably due to unfavorable positions of the substituents. Hydroxylation of the cyclic products took place basically in the case of the starting compounds already having one hydroxy group on the benzene ring. 

**Figure 5:**
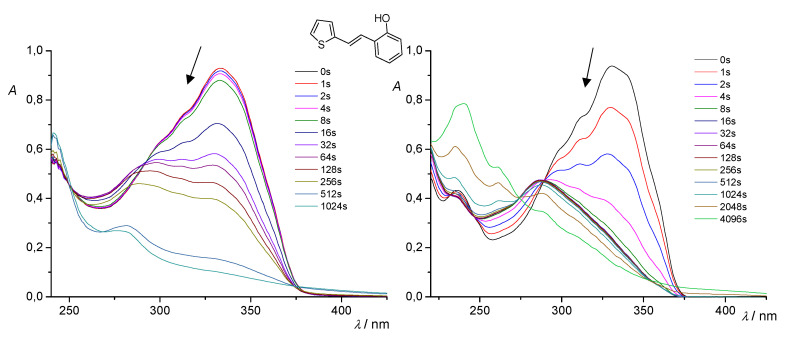
Spectral changes during the irradiation of **1** in ACN (**left**) *λ*_ir_ = 313 nm, *l* = 1 cm [19] and (**right**) *λ*_ir_ = 365 nm, *l* = 1 cm.

**Figure 6:**
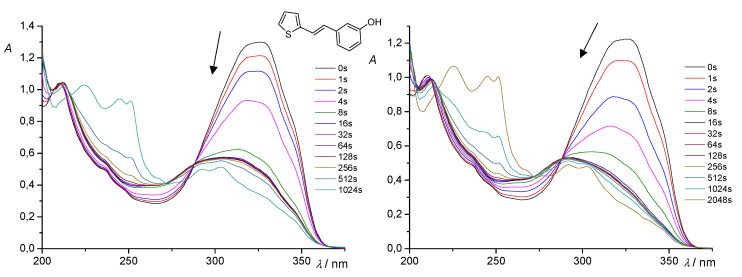
Spectral changes during the irradiation of **2** in ACN (**left**) *λ*_ir_ = 313 nm, *l* = 1 cm, and (**right**) *λ*_ir_ = 365 nm, *l* = 1 cm.

**Figure 7:**
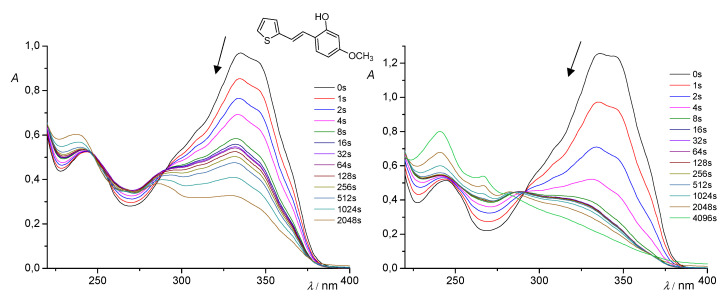
Spectral changes during the irradiation of **3** in ACN (**left**) *λ*_ir_ = 313 nm, *l* = 1 cm, and (**right**) *λ*_ir_ = 365 nm, *l* = 1 cm.

**Figure 8:**
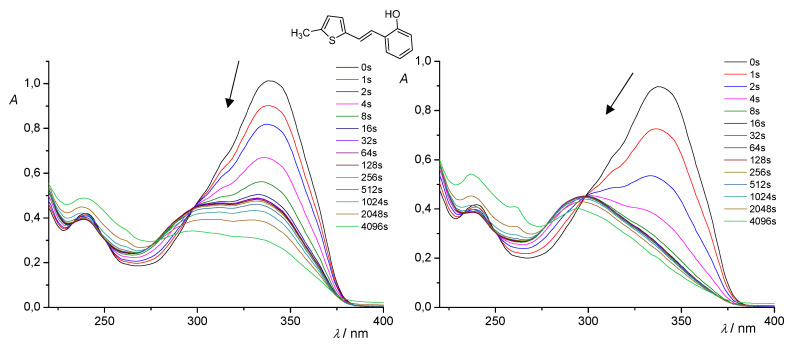
Spectral changes during the irradiation of **4** in ACN (**left**) *λ*_ir_ = 313 nm, *l* = 1 cm, and (**right**) *λ*_ir_ = 365 nm, *l* = 1 cm.

**Figure 9:**
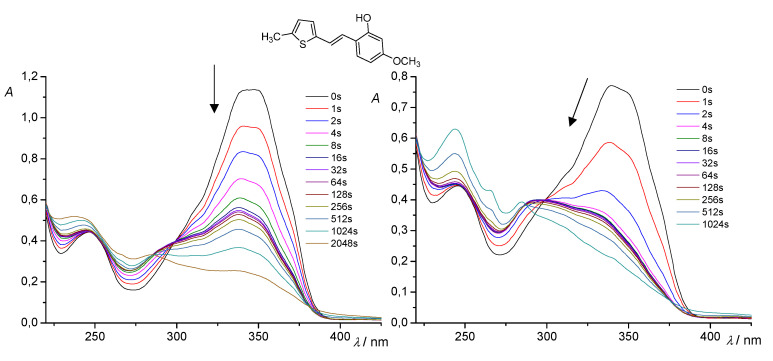
Spectral changes during the irradiation of **5** in ACN (**left**) *λ*_ir_ = 313 nm, *l* = 1 cm, and (**right**) *λ*_ir_ = 365 nm, *l* = 1 cm.

As for the spectral series recorded during the direct irradiations of the various starting compounds **1**–**5** in time at 313 and 365 nm (Figure 5, Figure 6, Figure 7, Figure 8 and Figure 9), they are rather similar. In all cases, the absorption spectrum of the starting *trans*-isomers gradually decreased and transformed to another species, which was the corresponding *cis*-isomers. At prolonged irradiations, the absorbance increased at shorter wavelengths, indicating that the products contain less unsaturated bonds. More specifically, in the case of compound **1**, isomerization at 313 nm is somewhat slower than at 365 nm (Figure 5). Of course, it should be kept in mind that these observations are at much lower concentrations of solutions in ACN than the preparative experiments. In the case of compound **2**, there are very similar tendencies of changes at both wavelengths (Figure 6), while in the case of **3,** the situation is similar to that of compound **1**, although, in the preparative experiment, there are significant differences apparently due to concentration (Figure 7). Illumination of compound **4** at 365 nm results in a faster change to the *cis*-isomer than at 313 nm and a faster shift of the maximum wavelength toward lower values (Figure 8). In the case of derivative **5**, the increase in absorbance at lower wavelengths, especially for experiments at 365 nm, clearly indicates the formation of the electrocyclization photoproduct (Figure 9).

### 2.2. Photochemical Transformations of Resveratrol Derivatives ***1**–**5*** in Flow Reactor

**Figure 10:**
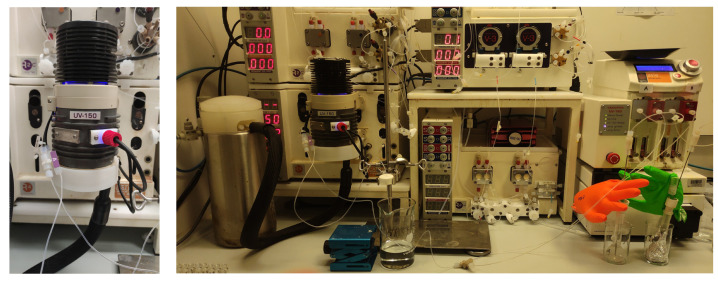
Flow photochemistry system.

Experiments on photochemical transformations of resveratrol analogs **1**–**5** were also carried out in a flow photochemical reactor (Figure 10) in order to see the influence of the reactor design and lamp type on the photochemical transformations under the following concentration condition: compounds **1**–**5**/photocatalyst = 10, flow rates 0.5 or 0.1 mL/min, applied wavelengths 365, 405, 420 and 450 nm at room temperature or at 40 °C. Table 4, Table 5, Table 6 and Table 7 summarize the yields of photoproducts obtained from the starting compound **1** achieved by photocatalysis using cationic and anionic complexes with manganese(III) metal center and the corresponding free-base cationic and anionic porphyrins, respectively. 

**Figure 11:**
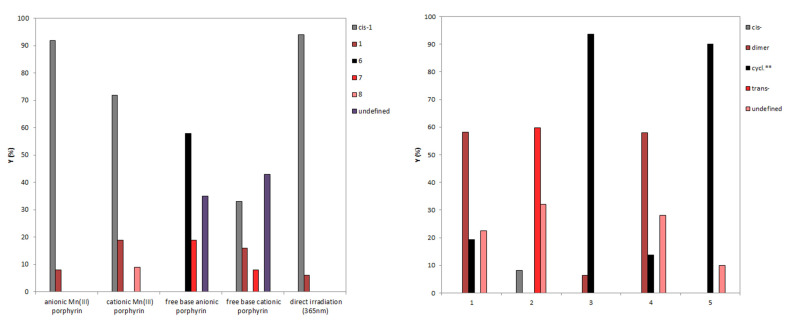
In parallel, (**left panel**) for resveratrol analog **1** showed the best results for all applied photocatalysts and for direct irradiation at 365 nm, and (**right panel**, ** product of photochemical electrocyclization reaction, see Table 8) for derivatives **1**–**5** display of utilization of photoproducts (%) under the best conditions with a photocatalyst (FbA, *τ* = 10 min, 420 nm), all in a flow reactor.

For compound **1**, the best conditions for selective isomerization to the *cis*-**1** are those at 365 nm using anionic Mn(III) prophyrin (flow rate 0.5 mL/min, Table 5) or by direct illumination without a photocatalyst at the same wavelength (Figure 11). Namely, compound **1** in the reaction without catalyst at 365 nm (flow rate 0.5 mL/min, direct irradiation) gave the following ratio, *cis*-**1** (94%):**1** (6%), without any other photoproducts or side-products. These are better results in comparison with those obtained in a batch reactor (Table 2) as the reaction is more selective toward *trans*-*cis* isomerization. As for the proven biologically active dimer **6**, the best reaction condition in the flow reactor is the use of an anionic free-base (flow rate 0.1 mL/min, Table 7), where there is neither the starting compound nor the *cis*-isomer present in the photomixture, but photoproducts **6** and **7** (electrocyclization product). This ratio of photoproducts toward the synthesis of dimer **6** is also better than that achieved in the batch reactor (Table 2).

The best results for the selective obtaining of the electrocyclization product **7** were achieved in the case of the use of free-base cationic porphyrin (flow rate 0.1 mL/min, Table 6) again more effectively than under the batch reaction conditions (Table 2).

As indicated, the most favorable outcomes of the reaction in the flow system were compared with those of the batch system on the basis of yield, conversion, and *trans*/*cis* ratio. Additionally, various process conditions such as temperature, flow rates and residence time were changed to investigate their influence on the process performance. As can be seen, the reaction was significantly faster in a flow reactor. With a residence time of only 2 min, higher yields of the targeted photoproducts were obtained compared to the batch reactor. The results provided clearly show the advantages of better mass transfer in flow reactors compared to batch reactors. It was also found that by changing (increasing) the temperature, the ratio of photoproducts remained similar as under room temperature except in the case of free-base anionic porphyrin in which more cyclization product **7** is formed compared to the dimeric product **6** (Table 4, Table 5, Table 6 and Table 7). 

The most favorable termination of the reaction for compounds **2**–**5** in the flow reactor is presented in Figure 11. By utilization of the photocatalysis of **1**–**5** under the most appropriate conditions for the photoproduct (dimer and electrocyclization product) formation (FbA, *τ* = 10 min, 420 nm) in a flow reactor, it can be clearly seen that compounds **1** and **4** with the same position and number of substituents on the aryl nucleus react very similarly. The main product is dimeric in about 60% yield. Derivatives **3** and **5**, which also have an identical aryl part of the stilbene skeleton as the main product, give the photocyclization product about 90% yield. Resveratrol analog **2**, possessing only one OH group in the *meta*-position, showed the weakest reactivity under the same conditions. It is obvious that the methyl substituent on the thiophene subunit does not affect sterically or electronically the outcome of the reaction in the flow reactor, while the crucial role in directing photocatalysis is played by the substitution on the aryl part of the molecule. Table 8 summarizes the conversion data and yields of photoproducts obtained from the starting compounds **1**–**5** under direct irradiations at 365 nm) and with a photocatalyst (FbA, *τ* = 10 min, 420 nm) in a flow reactor. As in the case of batch reactors and in flow reactors, Mn(III) complexes are more favorable as catalysts for isomerization than their free bases, while the anionic free base is most suitable for the formation of dimeric or electrocyclic photoproducts (except for **2**).

**Figure 12:**
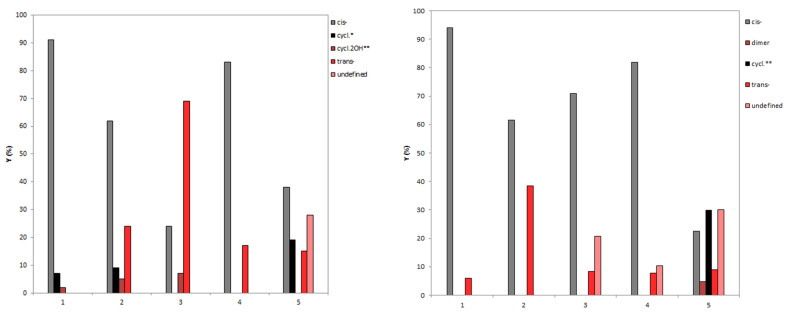
Yields (%) of photoproducts obtained and the remained *trans*-isomer of compounds **1**–**5** for reactions performed in (**left panel**, * product of photochemical electrocyclization reaction and ** cyclization products with 2OH functional groups, see Table 3) a batch reactor (*t* = 30 min) and (**right panel,** ** product of photochemical electrocyclization reaction, see Table 8) flow reactor (*τ* = 2′) using LED lamp of 365 nm (without photocatalyst).

Figure 12 compares yields (%) of photoproducts obtained and of the remained *trans*-isomer of compounds **1**–**5** for reactions performed in a batch reactor (*t* = 30′) and flow reactor (*τ* = 2′) using an LED lamp of 365 nm without photocatalyst. In this case, a better conversion to the corresponding *cis*-isomer is achieved in the case of compounds **1**, **3,** and **4** in the flow reactor. In the case of resveratrol analog **5**, the conversion is also somewhat better, but the reaction is also faster because, ultimately, there are fewer *cis*-**5** than in the batch reactor and more dimer and cyclization products. Again, compound **2**, which is the only one with an OH group in the *meta*-position of the aryl ring, is the least reactive, and it seems that the flow reactor slowed down the conversion but gave a more selective isomerization. As a final confirmation of the much higher efficiency of the photochemical transformations of resveratrol analogs **1**–**5** in the flow reactor, there are productivity values for the batch reactor range from 0.016 to 0.051 μmol mL^−1^ min^−1^ (on the basis of data from Table 3) and for the flow reactor, 0.201–0.500 μmol mL^−1^ min^−1^ (Table 8).

### 2.3. Computational Study

In order to explain the photochemical behavior of **1**, we performed a comprehensive computational exploration of its photoreactivity using both CASSCF//CASPT2 and TD-DFT methodologies (see Section 3 for complete Computational Details). As a first step, we optimized the structures for compound **1** at the CASSCF/6-31G* level of theory using an active space of 12 electrons in 12 orbitals, including all the π cloud and the corresponding π* orbitals; see the ESI for more details. The obtained geometries are described below. All the energies were computed under the MS-CASPT2 framework on top of CASSCF-optimized geometries considering four roots and weighing all with the same contribution into the averaged description. In the following Table 9, the relative energies in kcal/mol are shown taking as reference the ground state of the starting *trans*-isomer of **1** (*trans*-**1**).

As can be seen, there is a small energy difference between the two isomers of *cis*-**1,** being both ca. 3 kcal/mol more unstable than *trans*-**1**, proving this is the thermodynamically stable isomer, in agreement with the experimental results. In the case of *trans*-**1**, it should be marked that the brightest state corresponds to the excitation to S_2_, in contrast to the obtained for both *cis*-isomers, where the S_1_ became brighter. Also, it should be noted that for *trans*-isomer, the nature of S_1_ and S_2_ is the opposite at CASSCF and after CASPT2 correction, this will be relevant to compute the relaxation pathways starting from this isomer.

**Figure 13:**
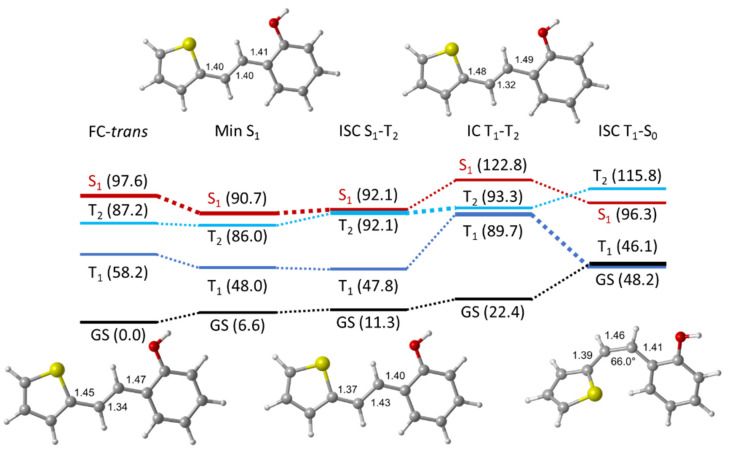
Isomerization profile. Energies referred to *trans*-**1** in the ground state in kcal/mol.

As seen before, direct photoreactivity of **1** seems to involve both photocyclization (formation of **7**) and photoisomerization (formation of *cis*-**1**, see Scheme 1) among other, more complex products. Thus, we initially focused on the reactive pathways leading to these products. The simplest approach was to consider a straightforward C=C photoisomerization process. However, unfruitful efforts were made to find any conical intersection (CI) point along the typical torsion and inversion mechanisms using CASSCF. As an alternative, we envisioned the possibility of the involvement of intermediates of different multiplicity, potentially caused by the thiophene moiety. For that, we considered the contribution of triplet states promoted by the sulfur-containing ring to participate in the isomerization process for the initial compound **1** to yield *cis*-**1**. This required the use of TD-DFT under the ωB97XD/6-31G* methodology (Figure 13). Thus, after recomputing the critical points along the potential energy surface, we found that the excitation of the thermodynamically stable *trans*-**1** yields on S_1_ evolving via an initial stretching to a high energy minimum with a minimal geometry distortion (Min S_1_). From here, an intersystem crossing point is readily accessible (ISC S_1_-T_2_, ca. 1 kcal/mol above), allowing the population of T_2_. This, in turn, easily converts to T_1,_ which evolves, rotating to two isoenergetic ISC points with the ground state. Both ISCs feature a torsion of 66° (ISC T_1_-S_0_, Figure 13) and 122°, respectively, being slightly lower in energy than the one at 66°.

**Figure 14:**
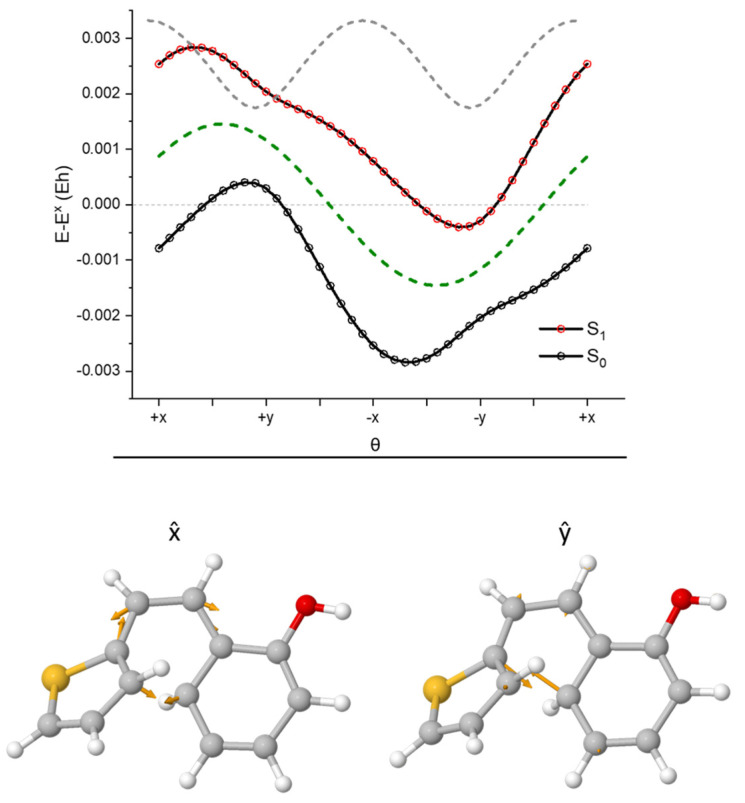
(**Top**) plot of S_0_ (black) and S_1_ (red) around the conical intersection varying angle θ at a distance of r = 0.01 Å, the dashed lines represent the average energy (green) and the energy difference (grey), the circles are the values obtained from single-point calculations. (**Bottom**) topology around the CI and the proper $\hat{x}$ (**left**) and $\hat{y}$ (**right**) vectors describing it.

This complex pathway explains the isomerization process to yield *cis*-**1**. Once the *cis*-isomer is obtained, it can easily rotate and convert between *cis*-**1** and *cis*-**1-isom**, with a negligible energy gap of 0.3 kcal/mol, computed at the SA-CASPT2//CASSCF level of theory (Table 9). Thus, both isomers should be present under the experimental conditions. Then, *cis*-**1-isom** could undergo a photochemical intramolecular cyclization as obtained by relaxation from the Franck-Condon region at the CASSCF level, passing through a small energy minimum. A typical conical intersection geometry was found at ca. 10 kcal/mol below the FC region. This CI point can be directly populated in the evolution of S_1_ out of the FC region. This CI presents a very sloped and single-path character according to its descriptors *p* = 2.27 and B = 1.21 (Table 10 and Figure 14), meaning sloped (*p* > 1) and single-path (B > 1) [20].

Thus, two main paths were computed in agreement with the experimental results of the direct irradiation of **1**. First, the starting material can isomerize with the participation of triplet state intermediates to yield the experimentally detected *cis*-isomer (*cis*-**1**). In a subsequent photoreaction under similar reaction conditions, the *cis*-isomer can cyclize following a pathway not available for the *trans*-isomer to yield the cyclic product **7** experimentally isolated. These linked photochemical reactions (together with the competing photoisomerization back to the *trans*-isomer explain the accumulation of **7** relatively low yields. In turn, this compound could react further to provide **8**.

## 3. Materials and Methods

### 3.1. General Remarks

Nuclear magnetic resonance (NMR) spectroscopic data for ^1^H and ^13^C nuclei were recorded at room temperature on Bruker Avance 300 MHz and 600 MHz spectrometers. Deuterated chloroform, CDCl_3_, with tetramethylsilane, TMS, as a standard, was used to record NMR spectra. Chemical shifts were expressed in parts per million, ppm (eng. parts per million). All solvents used were commercially available and purified by distillation. Anhydrous magnesium sulfate, MgSO_4_, was used to dry the organic layers after extraction. Column chromatography was performed on silica gel columns (60 Å, technical purity). Thin-layer chromatography was performed using plates coated with silica gel (0.2 mm, Kiselgel 60 F254). Abbreviations used in this experimental procedure were UV—ultraviolet spectrophotometry, PE—petroleum ether, E—diethyl ether, ACN—acetonitrile, and EtOH—ethanol. UV spectra were recorded on a UV/Vis spectrophotometer. Spectral changes during illumination were monitored for compounds dissolved in ACN. Before the measurement, the reaction mixture was purged with nitrogen for 15 min. Preparative batch photochemical reactions were carried out in a closed quartz cuvette using two photochemical reactors, Rayonet and Luzchem, equipped with UV lamps with wavelengths of 313 and 365 nm. All solvents were removed from the solutions using a rotary evaporator under reduced pressure. Preparative flow photochemical reactions were carried out in a Vapourtec UV-150 reactor equipped with LED lamps at 50% power; 2 mL; high purity PFA; OD: 1/16″; ID = 1.06 mm. Photocatalysts used in the experiments were cationic free base porphyrin (FbC): 5,10,15,20-tetrakis(1-methylpyridinium-4-yl) porphyrin (tetratosylate salt) (H_2_TMPyP4^+^ (+4 CH_3_C_6_H_4_SO^3−^, TMP-1363; cationic Mn(III)porphyrin (MnC): Mn(III) 5,10,15,20-tetrakis(1-methylpyridinium-4-yl) porphyrin pentachloride; anionic free base porphyrin (FbA): 5,10,15,20-tetrakis (4-sulfonatophenyl)porphyrin (tetrasodium salt dodecahydrate) and anionic Mn(III) porphyrin (MnA): Mn(III) 5,10,15,20-tetrakis(4-sulfonatophenyl) porphyrin chloride (acid form). 

Reaction mixtures dissolved in MeOH from both batch and flow experiments were analyzed by HPLC (1100 Series, Agilent, Santa Clara, CA, USA) with DAD detector and Waters XBridge C18 column (3.5 µm, 4.6 × 150 mm). The mobile phase consisted of solvent A (acetonitrile) and solvent B (0.2% vol. H_3_PO_4_ in water) with gradient elution from 90 to 10% B in 15 min, followed by keeping 10% B for 3 min, which was then replaced by 90% B kept for the next 2 min. The flow rate was 1 mL/min with a column temperature of 35 °C and UV detection at 320 nm. Mass spectra analyses were carried out on an HPLC (1290 Infinity II, Agilent Technologies, Santa Clara, CA, USA)—MS (6120 Quadrupole LC/MS, Agilent Technologies) with DAD detector and ACQUITY UPLC BEH C18 Column (1.7 μm, 2.1 × 50 mm). The flow rate was 0.900 mL/min with a column temperature of 40 °C. The mobile phase consisted of solvent A (0.1% formic acid in H_2_O) and solvent B (0.1% formic acid in acetonitrile). The composition of the mobile phase varied in the following way:
**Time, min****A, %****B, %****Flow, mL/min**0.0097.003.000.9001.500.00100.000.9001.900.00100.000.9002.0097.003.000.900

### 3.2. Synthesis of the Resveratrol Derivatives ***1**–**5***

The starting compounds **1***–***5**, as mixtures of *cis*- and *trans*-isomers, were synthesized by the Wittig reaction. The reaction mixtures were purged with nitrogen for 15 min before adding the reagents. In three-necked round-bottom flasks (100 mL), solutions of the appropriate phosphonium salts (11 mmol) were dissolved in 50 mL of absolute EtOH (dried on a 4 Å sieve). Sodium ethoxide solutions (11 mmol, 1.1 eq Na dissolved in 10 mL absolute ethanol) were added dropwise under strictly anhydrous conditions under a nitrogen atmosphere. Various commercially available aldehydes (11 mmol) were added directly to the mixed solutions. The reaction mixtures were allowed to stir for 72 h at room temperature under a nitrogen bubble. After removal of the solvent, absolute EtOH, using a rotary evaporator under reduced pressure, the oily reaction mixtures were extracted with toluene p.a. (3 × 25 mL). The organic layers were dried over anhydrous MgSO_4_. The products were isolated by column chromatography on a silica-gel column using PE/E as eluent and characterized by spectroscopic methods mainly as pure *trans*-isomers. The first fractions gave corresponding *cis*-isomers in small amounts for **1**, **3***–***5**, and the last fractions *trans*-isomers **1***–***5**. All isolated *cis*- and *trans*-isomers were published in our previous investigation [1].

*(E)-2-(2-(thiophen-2-yl)vinyl)phenol* (**1**) [1]: 495 mg, 82% isolated; white powder, m. p. 126–127 °C; *R_f_* (PE/DCM (50%)) = 0.33; UV (ACN) *λ*_max_/nm (*ε*/dm^3^ mol^−1^ cm^−1^) 335 (27,412); ^1^H NMR (CDCl_3_, 600 MHz) *δ*/ppm: 7.46 (dd, *J* = 7.7, 1.6 Hz, 1H), 7.28 (dt, *J* = 16.1, 0.8 Hz, 1H), 7.20–7.16 (m, 2H), 7.15–7.10 (m, 1H), 7.07 (dt, *J* = 3.7, 0.9 Hz, 1H), 6.99 (dd, *J* = 5.1, 3.5 Hz, 1H), 6.93 (td, *J* = 7.5, 1.2 Hz, 1H), 6.78 (dd, *J* = 8.1, 1.2 Hz, 1H), 4.97 (s, 1H); ^13^C NMR (CDCl_3_, 150 MHz) *δ*/ppm: 152.9, 143.3, 128.6, 127.6, 127.2, 126.0, 124.4, 124.3, 123.3, 122.7, 121.2, 115.9; MS (ESI) (*m*/*z*) (%, fragment): 202 (25), 105 (100).

*(E)-3-(2-(thiophen-2-yl)vinyl)phenol* (**2**) [1]: 13 mg, 5% isolated; white powder; *R_f_* (PE/E (18%)) = 0.24; ^1^H NMR (CDCl_3_, 600 MHz) *δ*/ppm: 7.22–7.18 (m, 3H), 7.06 (d, *J* = 3.9 Hz, 1H), 7.04 (d, *J* = 7.9 Hz, 1H), 7.00 (dd, *J* = 5.4, 3.3 Hz, 1H), 6.94 (t, *J* = 2.1 Hz, 1H), 6.86 (d, *J* = 15.9 Hz, 1H), 6.72 (dd, *J* = 7.9, 2.5 Hz, 1H), 4.78 (s, 3H); ^13^C NMR (CDCl_3_, 150 MHz) *δ*/ppm: 155.8, 142.7, 138.7, 129.9, 127.8, 127.6, 126.3, 124.5, 122.3, 119.3, 114.7, 112.7; MS (ESI) (*m*/*z*) (%, fragment): 203 (100); HRMS (*m*/*z*) for C_12_H_10_OS: [M + H]^+^_calculated_ = 202.0452, [M + H]^+^_measured_ = 202.0458.

*(E)-5-methoxy-2-(2-(thiophen-2-yl)vinyl)phenol* (**3**) [1]: 60 mg, 40% isolated; yellow powder; m. p. 91–94 °C; *R_f_* (PE/E (60%)) = 0.35; UV (ACN) *λ*_max_/nm (*ε*/dm^3^ mol^−1^ cm^−1^) 336 (23,732), 244 (9367), 211 (15320); ^1^H NMR (CDCl_3_, 600 MHz) *δ*/ppm: 7.36 (d, *J* = 8.7 Hz, 1H), 7.17–7.14 (m, 2H), 7.06 (d, *J* = 16.2 Hz, 1H), 7.03 (d, *J* = 3.4 Hz, 1H), 6.98 (dd, *J* = 4.9, 3.7 Hz, 1H), 6.51 (dd, *J* = 8.5, 2.6 Hz, 1H), 6.37 (d, *J* = 2.6 Hz, 1H), 5.03 (s, 1H), 3.79 (s, 3H); ^13^C NMR (CDCl_3_, 150 MHz) *δ*/ppm: 160.2, 153.9, 143.6, 128.2, 127.2, 125.4, 123.8, 122.6, 121.5, 117.3, 107.1, 101.9, 55.4; MS (ESI) (*m*/*z*) (%, fragment): 233 (100); HRMS (*m*/*z*) for C_13_H_12_O_2_S: [M + H]^+^_calculated_ = 232.0558, [M + H]^+^_measured_ = 232.0556.

*(E)-2-(2-(5-methylthiophen-2-yl)vinyl)phenol* (**4**) [1]: 95 mg, 40% isolated; yellow powder; m. p. 88–92 °C; *R_f_* (PE/E (20%)) = 0.54; UV (ACN) *λ*_max_/nm (*ε*/dm^3^ mol^−1^ cm^−1^) 339 (26,378), 240 (10,925), 208 (19,311); ^1^H NMR (CDCl_3_, 600 MHz) *δ*/ppm: 7.44 (d, *J* = 7.3 Hz, 1H), 7.18 (d, *J* = 16.6 Hz, 1H), 7.11 (t, *J* = 7.9 Hz, 1H), 7.01 (d, *J* = 16.6 Hz, 1H), 6.92 (t, *J* = 7.6 Hz, 1H), 6.85 (d, *J* = 2.9 Hz, 1H), 6.78 (d, *J* = 7.9 Hz, 1H), 6.64–6.64 (m, 1H), 4.93 (s, 1H), 2.48 (s, 3H); ^13^C NMR (CDCl_3_, 150 MHz) *δ*/ppm: 152.8, 141.2, 139.3, 128.3, 127.1, 126.3, 125.7, 124.5 123.8, 121.4, 121.2, 115.9, 115.6; MS (ESI) (*m*/*z*) (%, fragment): 217 (100); HRMS (*m*/*z*) for C_13_H_12_OS: [M + H]^+^_calculated_ = 216.0609, [M + H]^+^_measured_ = 216.0607.

*(E)-5-methoxy-2-(2-(5-methythiophen-2-yl)vinyl)phenol* (**5**) [1]: 5 mg, 6% isolated; yellow powder; m.p. 91–95 °C; *R_f_* (PE/E (60%)) = 0.45; ^1^H NMR (CDCl_3_, 600 MHz) *δ*/ppm: 7.33 (d, *J* = 8.4 Hz, 1H), 7.06 (d, *J* = 16.3 Hz, 1H), 6.91 (d, *J* = 16.3 Hz, 1H), 6.80 (d, *J* = 3.4 Hz, 1H), 6.62 (d, *J* = 3.1 Hz, 1H), 6.50 (dd, *J* = 8.7, 2.6 Hz, 1H), 6.37 (d, *J* = 2.5 Hz, 1H), 5.03 (s, 1H), 3.78 (s, 3H), 2.47 (s, 3H); ^13^C NMR (CDCl_3_, 150 MHz) *δ*/ppm: 160.0, 153.9, 141.5, 138.7, 128.0, 125.7, 121.9, 121.3, 117.5, 107.1, 101.9, 55.4, 15.6; MS (ESI) (*m*/*z*) (%, fragment): 245 (100); HRMS (*m*/*z*) for C_14_H_14_O_2_S: [M + H]^+^_calculated_ = 246.0715, [M + H]^+^_measured_ = 246.0712.

### 3.3. Photochemistry of the Resveratrol Derivatives ***1**–**5*** in a Batch Reactor

On pure isomers **1***–***5**, direct photochemical preparative illuminations were carried out without the use of catalysts at wavelengths of 313 nm and 365 nm in the Rayonet RPR 100 photoreactor, using 10 halogen lamps for all experiments. The irradiations were carried out in 30 min with about 0.001 mol/dm^3^ working concentrations of the studied compounds **1***–***5**, without stirring purges air/oxygen before irradiation, after which the products were determined using HPLC and NMR spectroscopy. 

*(Z)-2-(2-(thiophen-2-yl)vinyl)phenol* (*cis*-**1**) [2]: 60 mg (isolated max. 62%), colorless oil; *R_f_* (PE/DCM (50%)) = 0.44; UV (CH_3_CN) *λ*_max_/nm (*ε*/dm^3^ mol^−1^ cm^−1^) 289 (26,313); ^1^H-NMR (CDCl_3_, 600 MHz) *δ*/ppm: 7.28 (tdd, *J* = 7.9, 1.7, 0.7 Hz, 1H), 7.20 (dt, *J* = 7.7, 1.2 Hz, 1H), 7.13 (dt, *J* = 5.1, 0.9 Hz, 1H), 7.01–6.93 (m, 4H), 6.90 (dd, *J* = 5.1, 3.6 Hz, 1H), 6.43 (d, *J* = 11.6 Hz, 1H), 5.08 (s, 1H); ^13^C-NMR (CDCl_3_, 150 MHz) *δ*/ppm: 153.2 (s), 138.7 (s), 129.2 (d), 128.3 (d), 127.6 (d), 126.7 (s), 125.4 (d), 125.1 (d), 123.5 (d), 122.9 (d), 118.9 (d), 114.4 (d); MS (ESI) *m*/*z* (%, fragment): 203 (100).

*(4bS,5R,10bS,11R)-5,11-di(thiophen-2-yl)-4b,5,10b,11-tetrahydrochromeno[4,3-c]chromene* (**6**) [2]: 6 mg (isolated max. 15%); white powder; m.p. = 155–158 °C; *R_f_* (PE/DCM (50%)) = 0.65; UV (CH_3_CN) *λ*_max_/nm (*ε*/dm^3^ mol^−1^ cm^−1^) 283 (25,196), 276 (27,036); ^1^H-NMR (CDCl_3_, 600 MHz) *δ*/ppm: 7.40 (dd, *J* = 5.1, 1.2 Hz, 1H), 7.25 (dd, *J* = 4.4, 1.5, Hz, 1H), 7.13 (dd, *J* = 8.7, 1.8 Hz, 1H), 7.09–7.07 (m, 1H), 6.88 (dd, *J* = 8.1, 1.2 Hz, 1H), 6.83–6.76 (m, 2H), 5.70–5.64 (m, 1H), 3.60–3.53 (m, 1H); ^13^C-NMR (CDCl_3_, 150 MHz) *δ*/ppm: 144.3 (s), 130.8 (s), 128.1 (d), 127.1 (d), 127.0 (d), 126.6 (d), 125.7 (d), 125.4 (s), 121.5 (d), 117.9 (d), 42.6 (d), 29.7 (d); MS (ESI) *m*/*z* (%, fragment): 342 (5), 301 (15), 279 (100).

### 3.4. Photochemistry of the Resveratrol Derivatives ***1**–**5*** in a Flow Reactor

**Figure 15:**
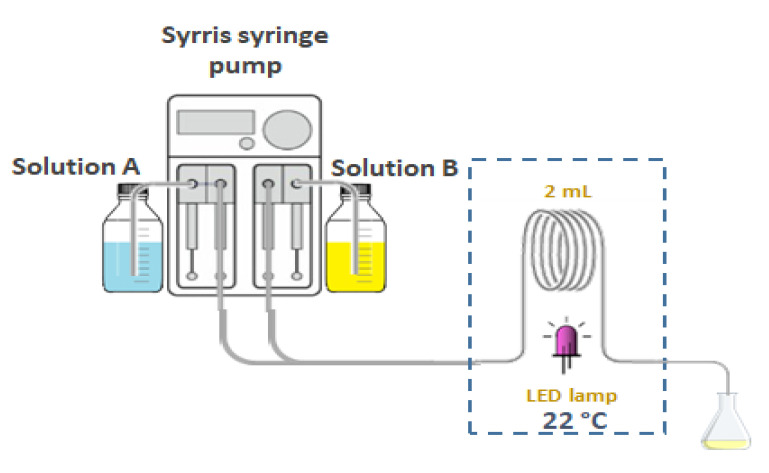
Experimental flow scheme.

The following solutions were prepared for the experimental procedure in a flow reactor (Figure 15). Solution A: compounds **1***–***5** were dissolved in air-saturated technical acetone in a volumetric flask of 20 mL so that the concentration of the solution was 0.001 M. Solutions were placed under air-filled balloon; solution B: catalysts MnC, MnA, FbC or FbA were dissolved in oxygen saturated H_2_O in a volumetric flask of 20 mL so that the concentration of the solution was 0.0001 M. Solutions were placed under oxygen filled balloon. Two residence times were examined: (a) *τ* = 2 min; (b) *τ* = 10 min, as well as four LED lamps: (c) 365 nm; (d) 405 nm; (e) 420 nm; (f) 450 nm.

Solution A (compounds **1***–***5**; Syrris syringe pump; (a) 0.500 mL/min; (b) 0.100 mL/min) and solution B (catalysts MnC, MnA, FbC or FbA; Syrris syringe pump; (a) 0.500 mL/min; (b) 0.100 mL/min) were pumped through the reactor (Vapourtec UV-150 reactor equipped with LED lamps (c); (d); (e); (f) at 50% power; 2 mL; high purity PFA; OD: 1/16″; ID = 1.06 mm). One milliliter of the reaction mixtures was collected per vial (the number of vials varied from 4 to 8). Every combination of compound **1** and catalysts MnC, MnA, FbC, or FbA was examined for residence times (a) and (b) and LED lamps (c), (d), (e), and (f) at 22 °C as well as direct irradiation for (a) and (c).

The following conditions were examined at 40 °C for compound **1**:
**Compound 1****Catalyst****MnC****MnA****FbC****FbA***τ*, min102210*λ*, nm365365405420

The following conditions were examined at 22 °C for compounds **2**–**5**: direct irradiation, *τ* = 2 min, *λ* = 365 nm and catalyst FbA, *τ* = 10 min, *λ* = 420 nm. The quantities of substances used to prepare the solutions were as follows:
**Compound*****c*, mol/dm^3^*****V* (acetone), mL*****m*, mg*****n*, mol****1**0.001204.050.0002**2**0.001204.050.0002**3**0.001204.640.0002**4**0.001204.320.0002**5**0.001204.920.0002**Catalyst*****c*, mol/dm^3^*****V* (H_2_O), mL*****m*, mg*****n*, mol**MnC0.0001201.820.00002MnA0.0001202.060.00002FbC0.0001202.730.00002FbA0.0001202.080.00002

### 3.5. Cholinesterase Inhibitory Activity

Acetylcholinesterase (AChE) and butyrylcholinesterase (BChE) inhibitory potentials were determined using modified Ellman’s method [21] as described earlier [1]. The results are expressed as percentage inhibition of enzyme activity and IC_50_.

### 3.6. Computational Study

All CASSCF//CASPT2 calculations were carried out using an active space of 12 electrons and 12 orbitals, as described below, using the 6-31G* basis set and four states equally weighted in the averaged wavefunction. The computations were carried out using the 23.02 version of OpenMOLCAS [22]. All geometries were optimized by CASSCF, and the energy was reevaluated at the CASPT2 level. For the study at the TD-DFT level, the ωB97XD/6-31G* was used together with the Tamm-Dancoff approximation to better address triplet energies. For finding the critical minimum energy crossing points, the easyMECP software [23] was used, also including the TDA approach. All TD-DFT calculations were performed using Gaussian 16 Rev C.01 [24].

## 4. Conclusions

The last biological tests have shown that some resveratrol analogs exhibited significant antioxidative and cholinesterase inhibitory potential, as evidenced by lower IC_50_ values compared to the established standards, resveratrol and galantamine, respectively. Photochemical transformations were made in parallel on those compounds in the presence of porphyrin photocatalysts in batch and microreactor, showing the significant advantage of flow photochemistry concerning productivity, selectivity, and yields. In this research, the products of photocatalysis and direct irradiation (photolysis) of resveratrol analogs have been compared to elucidate how the types and ratios of the products depend on the excitation energy, to reveal the effects of the substituent on the photoinduced reactions and to rationalize experimentally and computationally the nature and ratio of the obtained products. Generally, the type of photocatalyst, retention time, and wavelength determine the selectivity of photochemical transformations that can be directed in this way. Two main paths were computed in agreement with the experimental results: isomerization with the participation of triplet state intermediates to yield the experimentally detected *cis*-isomers and their subsequent cyclization following a pathway not available for the *trans*-isomers. The investigation of five model compounds confirmed the advantages of the flow photoreactor in the photochemical reactions of heterocyclic resveratrol analogs. As a final confirmation of the much higher efficiency of the photochemical transformations of resveratrol derivatives **1**–**5** in the flow reactor, there are productivity values that for the batch reactor range from 0.016 to 0.051 μmol mL^−1^ min^−1^ and for the flow reactor 0.201–0.500 μmol mL^−1^ min^−1^.

## Figures and Tables

**Table 1:** Inhibition potency of the dimeric photoproduct **6**.

AChE		BChE **	
IC_50_/μM	% Inhibition *	IC_50_/μM	% Inhibition *
-	16.7 ± 0.6 (250)	9.3	83.2 ± 0.9 (250)

* Numbers given in parentheses represent maximal concentrations tested in μM. ** IC_50_ (Galantamine—standard reversible inhibitor, activity for BChE) = 7.9 μM.

**Table 2:** Yields (%) of photoproducts obtained from the starting compound **1** under direct irradiation or using Mn(III) and free base cationic/anionic porphyrins for reactions performed in a batch reactor (followed by HPLC).

Reaction Type	Photocatalyst/ Wavelength (nm)	Conversion (%)	Yields of Photoproducts (%)							
			Irr. Time	*cis*-1	6	7	8	9	1	Undefined
Direct irradiation	313	80	30 min	52	8	3	1	16	20	-
	365	100	30 min	91	-	7	2	-	-	-
Photocatalytic reaction [2]	free base cationic (>380)	80	2 h	31	25	-	2	1	20	21
	free base anionic (>380)	97	2 h	15	18	-	-	-	3	64
	cationic Mn(III) (>380)	93	6 h	74	-	-	13	1	7	5
	anionic Mn(III) (>380)	98	6 h	54	10	-	3	2	2	29

**Table 3:** Yields (%) of photoproducts obtained from the starting compounds **1**–**5** under direct irradiation for reactions performed in a batch reactor (followed by HPLC).

Compound	Wavelength (nm)	Conversion (%)	Yields of Photoproducts (%)						
			Irr. Time	*cis*-	Dimer	Cycl. *	Cycl.2OH **	*trans*-	Undefined
**1**	313	80	30 min	52	8	3	1	20	16
	365	100	30 min	91	-	7	2	-	-
**2**	313	91	30 min	65	-	12	4	19	-
	365	93	30 min	62	-	9	5	24	-
**3**	313	17	30 min	17	-	-	-	83	-
	365	31	30 min	24	-	7	-	69	-
**4**	313	75	30 min	58	-	2	-	25	15
	365	83	30 min	83	-	-	-	17	-
**5**	313	90	30 min	45	-	15	-	10	30
	365	85	30 min	38	-	19	-	15	28

* product of photochemical electrocyclization depending on the starting compound (**7**, **11**, **13**–**15**, Scheme 1, Scheme 2 and Scheme 3); ** cyclization products with 2OH functional groups (**8** and **12**, Scheme 1 and Scheme 2).

**Table 4:** Yields (%) of photoproducts obtained from the starting compound **1** using cationic Mn(III) porphyrin (MnC) for reactions performed in a flow reactor (followed by HPLC).

Retention Time (min)	Wavelength (nm)/Temp. °C	Flow Rate (mL/min)	Conversion (%)	Selectivity (*cis*/*trans* Ratio)	Yields of Photoproducts (%)					
					*cis*-1	6	7	8	1	Undefined
2	450/22	0.5	5.8	0.025	2.4	-	-	-	94.2	3.4
	420/22	0.5	2.6	0.026	2.5	-	-	-	97.4	-
	405/22	0.5	33.8	0.421	27.9	-	-	-	66.2	5.9
	365/22	0.5	68.7	1.885	59.0	-	-	1.7	31.3	8.0
10	450/22	0.1	4.6	0.048	4.6	-	-	-	95.4	-
	420/22	0.1	4.0	0.042	4.0	-	-	-	96.0	-
	405/22	0.1	60.9	1.281	50.1	-	-	-	39.1	10.8
	**365/22**	**0.1**	**81.0**	**3.789**	**72.0**	-	-	**9.0**	**19.0**	-
10	**365/40**	**0.1**	**89.2**	**7.806**	**84.3**	-	-	**4.9**	**10.8**	-

HPLC analyses values read at 320 nm.

**Table 5:** Yields (%) of photoproducts obtained from the starting compound **1** using anionic Mn(III) porphyrin (MnA) for reactions performed in a flow reactor (followed by HPLC).

Retention Time (min)	Wavelength (nm)/Temp. °C	Flow Rate (mL/min)	Conversion (%)	Selectivity (*cis*/*trans* Ratio)	Yields of Photoproducts (%)					
					*cis*-1	6	7	8	1	Undefined
2	450/22	0.5	8.5	0.093	8.5	-	-	-	91.5	-
	420/22	0.5	11.0	0.124	11.0	-	-	-	89.0	-
	405/22	0.5	17.8	0.216	17.8	-	-	-	82.2	-
	**365/22**	**0.5**	**91.9**	**11.346**	**91.9**	-	-	-	**8.1**	-
10	450/22	0.1	9.0	0.099	9.0	-	-	-	91.0	-
	420/22	0.1	17.0	0.144	12.0	-	-	-	83.0	5.0
	405/22	0.1	69.6	2.006	61.0	-	-	-	30.4	8.6
	365/22	0.1	82.8	4.221	72.6	-	3.1	-	17.2	7.1
2	**365/40**	**0.5**	**90.0**	**8.790**	**87.9**	**2.1**	-	-	**10.0**	-

HPLC analyses values read at 320 nm.

**Table 6:** Yields (%) of photoproducts obtained from the starting compound **1** using free-base cationic porphyrin (FbK) for reactions performed in a flow reactor (followed by HPLC).

Retention Time (min)	Wavelength (nm)/Temp. °C	Flow Rate (mL/min)	Conversion (%)	Selectivity (*cis*/*trans* Ratio)	Yields of Photoproducts (%)					
					*cis*-1	6	7	8	1	Undefined
2	450/22	0.5	27.3	0.292	21.2	-	6.1	-	72.7	-
	420/22	0.5	74.1	0.923	23.9	-	7.7	3.2	25.9	39.3
	**405/22**	**0.5**	**83.8**	**2.068**	**33.5**	-	**8.0**	-	**16.2**	**42.3**
	365/22	The catalysts do not absorb on this wavelength								
10	450/22	0.1	92.4	0.553	4.2	-	64.8	8.2	7.6	15.2
	420/22	0.1	100.0	-	-	-	76.9	11.1	-	12.0
	405/22	0.1	100.0	-	-	-	78.0	13.0	-	9.9
	365/22	The catalysts do not absorb on this wavelength								
2	**405/40**	**0.5**	**64.2**	**0.958**	**34.3**	-	**16.4**	-	**35.8**	**18.4**

HPLC analyses values read at 320 nm.

**Table 7:** Yields (%) of photoproducts obtained from the starting compound **1** using free-base anionic porphyrin (FbA) for reactions performed in a flow reactor (followed by HPLC).

Retention Time (min)	Wavelength (nm)/Temp. °C	Flow Rate (mL/min)	Conversion (%)	Selectivity (*cis*/*trans* Ratio)	Yields of Photoproducts (%)					
					*cis*-1	6	7	8	1	Undefined
2	450/22	0.5	22.7	0.107	8.3	-	-	-	77.3	14.4
	420/22	0.5	88.8	0.205	2.3	32.5	44.8	-	11.2	9.2
	405/22	0.5	92.5	0.707	5.3	41.0	46.2	-	7.5	-
	365/22	The catalysts does not absorb on this wavelength								
10	450/22	0.1	100	-	-	34.9	32.0	-	-	33.1
	**420/22**	**0.1**	**100**	-	-	**58.1**	**19.3**	-	-	**22.6**
	405/22	0.1	100	-	-	39.3	39.6	-	-	21.1
	365/22	The catalysts does not absorb on this wavelength								
10	**420/40**	**0.1**	**100**	-	-	**4**	**45.1**	-	-	**50.9**

HPLC analyses values read at 320 nm.

**Table 8:** Most successful photochemical reactions of the starting compounds **1**–**5** were performed in a flow reactor (followed by HPLC *).

Comp.	Catalyst	Retention Time (min)	Wavelength (nm)/Temp. °C	Flow Rate (mL/min)	Conversion (%)	Productivity (μmol mL^−1^ min^−1^)	Yields of Photoproducts (%)				
							*cis*-	Dimer	Cycl. **	*trans*-	Undefined
**1**	-	2	365/22	0.5	94	0.470	94.0	-	-	6.0	-
**2**	-	2	365/22	0.5	61.6	0.308	61.6	-	-	38.4	-
**3**	-	2	365/22	0.5	91.7	0.458	71.0	-	-	8.3	20.7
**4**	-	2	365/22	0.5	92.2	0.461	81.9	-	-	7.8	10.3
**5**	-	2	365/22	0.5	91.0	0.455	22.5	4.8	29.8	9.0	30.1
**1**	FbA	10	420/22	0.1	100	0.500	-	58.1	19.3	-	22.6
**2**	FbA	10	420/22	0.1	40.2	0.201	8.1	-	-	59.8	32.1
**3**	FbA	10	420/22	0.1	100	0.500	-	6.4	93.6	-	-
**4**	FbA	10	420/22	0.1	100	0.500	-	58.0	13.8	-	28.1
**5**	FbA	10	420/22	0.1	100	0.500	-	-	90.1	-	9.9

HPLC analyses values read at 320 nm; ** product of photochemical electrocyclization reaction.

**Table 9:** Relative energies at the SA-CASPT2//CASSCF level of the critical points on the potential energy surface for **1** (energies in kcal/mol relative to the most stable *trans***-1** isomer).

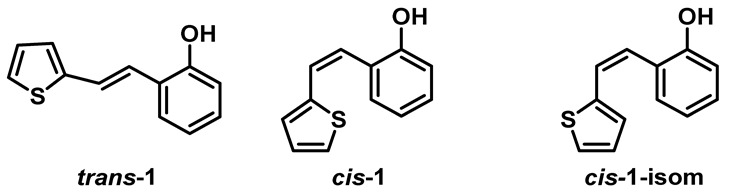			
	***trans*** **-1**	***cis*** **-1**	***cis*** **-1-Isom**
S_0_	0	2.91	3.27
S_1_	95.82	112.62	110.16
S_2_	117.61	143.75	133.81
S_3_	121.78	151.40	153.03

**Table 10:** Relative energies at the CASPT2 level of the critical points on the potential energy surface for **1** (energies in kcal/mol relative to the most stable *trans***-1** isomer).

	FC-*cis*-isom	S_1_Min_cis-isom_	CI-cyc
S_0_	3.27	11.66	99.85
S_1_	110.16	101.68	91.60
S_2_	133.81	121.86	143.88
S_3_	153.03	135.28	158.58

## Data Availability

The data presented in this study are available upon request from the corresponding author. The data are not publicly available due to privacy.

## Supplementary Materials

The following supporting information can be downloaded at https://www.mdpi.com/article/10.3390/molecules29010201/s1. 1. Orbitals included in the active space (12,12) (Figure S1); 2. Optimized Geometries coordinates; 3. Spectral series recorded during the direct irradiations of the various cis-isomers in time at 313 and 365 nm (Figures S2–S4); 4. Chromatograms for data in Table 8 and Table 5. NMR spectra of starting compounds, photoproducts and photomixtures (Figures S5–S34).

## References

[1] Mlakić M., Odak I., Barić D., Talić S., Šagud I., Štefanić Z., Molčanov K., Lasić Z., Kovačević B., Škorić I. (2024). New resveratrol analogs as improved biologically active structures: Design, synthesis and computational modeling. Bioorg. Chem..

[2] Mlakić M., Ljubić A., Šalić A., Zelić B., Horváth O., Milašinović V., Gojun M., Molčanov K., Škorić I. (2022). Photocatalytic transformations of the resveratrol derivative in microflow reactor. Catalysts.

[3] Marchiori C.F.N., Damas G.B., Araujo C.M. (2022). Tuning the photocatalytic properties of porphyrins for hydrogen evolution reaction: An in-silico design strategy. J. Power Sources Adv..

[4] Rybicka-Jasińska K., Wdowik T., Łuczak K., Wierzba A.J., Drapała O., Gryko D. (2022). Porphyrins as Promising Photocatalysts for Red-Light-Induced Functionalizations of Biomolecules. ACS Org. Inorg. Au.

[5] Zhang Y., Ren K., Wang L., Wang L., Fan Z. (2022). Porphyrin-based heterogeneous photocatalysts for solar energy conversion. Chinese Chem. Lett..

[6] Kuramochi Y., Satake A. (2023). Porphyrins Acting as Photosensitizers in the Photocatalytic CO_2_ Reduction Reaction. Catalysts.

[7] O’Neill J.S., Kearney L., Brandon M.P., Pryce M.T. (2022). Design components of porphyrin-based photocatalytic hydrogen evolution systems: A review. Coord. Chem. Rev..

[8] Wu Y., Liu J.-C., Li R.-Z., Ci C.-G. (2022). Different metal upper porphyrin based self-assembly sensitizers for application in efficient dye-sensitized solar cells. Polyhedron.

[9] Zhang Y., Higashino T., Imahori H. (2023). Molecular designs, synthetic strategies, and properties for porphyrins as sensitizers in dye-sensitized solar cells. J. Mater. Chem. A.

[10] Oyim J., Amuhaya E., Matshitse R., Mack J., Nyokong T. (2022). Integrated photocatalyst adsorbents based on porphyrin anchored to activated carbon granules for water treatment. Carbon Trends.

[11] Luo H., Yu W., Chen S., Wang Z., Tian Z., He J., Liu Y. (2022). Application of metalloporphyrin sensitizers for the treatment or diagnosis of tumors. J. Chem. Res..

[12] Isokuortti J., Kuntze K., Virkki M., Ahmed Z., Vuorimaa-Laukkanen E., Filatov M.A., Turshatov A., Laaksonen T., Priimagi A., Durandin N.A. (2021). Expanding excitation wavelengths for azobenzene photoswitching into the near-infrared range via endothermic triplet energy transfer. Chem. Sci..

[13] Zhang R., Horner J.H., Newcomb M. (2005). Laser Flash Photolysis Generation and Kinetic Studies of Porphyrin−Manganese−Oxo Intermediates. Rate Constants for Oxidations Effected by Porphyrin−Mn(V)−Oxo Species and Apparent Disproportionation Equilibrium Constants for Porphyrin−Mn(IV)−Oxo Species. J. Am. Chem. Soc..

[14] Newcomb M., Zhang R., Pan Z., Harischandra D., Chandrasena R., Horner J., Martinez II E. (2006). Laser flash photolysis production of metal-oxo derivatives and direct kinetic studies of their oxidation reactions. Catal. Today.

[15] Zhang R., Newcomb M. (2008). Laser Flash Photolysis Generation of High-Valent Transition Metal−Oxo Species: Insights from Kinetic Studies in Real Time. Acc. Chem. Res..

[16] Ahadi E., Hosseini-Monfared H., Spieß A., Janiak C. (2020). Photocatalytic asymmetric epoxidation of trans -stilbene with manganese–porphyrin/graphene-oxide nanocomposite and molecular oxygen: Axial ligand effect. Catal. Sci. Technol..

[17] Horspool W.M., Song P.S. (1995). CRC Handbook of Organic Photochemistry and Photobiology.

[18] Griesbeck A., Oelgemöller M., Ghetti F. (2012). CRC Handbook of Organic Photochemistry and Photobiology.

[19] Mlakić M., Rajič L., Ljubić A., Vušak V., Zelić B., Gojun M., Odak I., Čule I., Šagud I., Šalić A. (2022). Synthesis of new heterocyclic resveratrol analogues in milli- and microreactors: Intensification of the Wittig reaction. J. Flow Chem..

[20] Galván I.F., Delcey M.G., Pedersen T.B., Aquilante F., Lindh R. (2016). Analytical state-average complete-active-space self-consistent field nonadiabatic coupling cevtors: Impletation with density-fitted two-electron integrals and application to conical intersections. J. Chem. Theory Comput..

[21] Ellman G.L., Courtnex K.D., Andres V., Featherstone R.M. (1961). A new and rapid colorimetric determination of acetylcholinesterase activity. Biochem. Pharmacol..

[22] Li Manni G., Galván I.F., Alavi A., Aleotti F., Aquilante F., Autschbach J., Avagliano D., Baiardi A., Bao J.J., Battaglia S. (2023). The OpenMolcas Web: A community-driven approach to advancing computational chemistry. J. Chem. Theory Comput..

[23] https://github.com/jaimergp/easymecp.

[24] Frisch M.J., Trucks G.W., Schlegel H.B., Scuseria G.E., Robb M.A., Cheeseman J.R., Scalmani G., Barone V., Petersson G.A., Nakatsuji H. (2019). Gaussian 16, Revision C.01.

