# Supplementary material for: Photochemical Transformations of Diverse Biologically Active Resveratrol Analogs in Batch and Flow Reactors
Source: Molecules. 2023 Dec 29;29(1):201. doi: 10.3390/molecules29010201 (PMC10781098; doi:10.3390/molecules29010201)
Supplement: Supplementary file 1 [file molecules-29-00201-s001.zip › molecules-2775530-supplementary.pdf]

Article

Photochemical Transformations of Diverse Biologically Active Resveratrol Analogs in Batch and Flow Reactors

Milena Mlakić [1], Hana Perinić [1,2], Vitomir Vušak [2,*], Ottó Horváth [3], Diego Sampedro [4], Raúl Losantos [4], Ilijana Odak [5] and Irena Škorić [1,*]

1 Department of Organic Chemistry, Faculty of Chemical Engineering and Technology, University of Zagreb, Marulićev trg 19, HR-10 000 Zagreb, Croatia; mdragojev@fkit.unizg.hr (M.M.); hperinic@fkit.hr (H.P.)
2 Pliva R&D, Teva Pharmaceutical Industries Ltd., Prilaz baruna Filipovića 29, HR-10 000 Zagreb, Croatia
3 Environmental and Inorganic Photochemistry Research Group, Center for Natural Sciences, Faculty of Engineering, University of Pannonia, P.O. Box 1158, H-8210 Veszprém, Hungary; horvath.otto@mk.uni-pannon.hu
4 Departamento de Química, Centro de Investigación en Síntesis Química (CISQ), Universidad de La Rioja, 26006 Logroño, Spain; diego.sampedro@unirioja.es (D.S.); raul.losantosc@unirioja.es (R.L.)
5 Department of Chemistry, Faculty of Science and Education, University of Mostar, Matice Hrvatske bb, 88000 Mostar, Bosnia and Herzegovina; ilijana.odak@fpmoz.sum.ba
* Correspondence: vitomir.vusak@pliva.com (V.V.); iskoric@fkit.unizg.hr (I.Š.)

Content:

1. Orbitals included in the active space (12,12)…………………………………1

2. Optimized Geometries coordinates………………………………………………………...2

3. Spectral series recorded during the direct irradiations of the various *cis*-isomers in time at 313 and 365 nm………………………………………………………………………..9

4. Chromatograms for data in Table 8………………………………………………………. 11

5. NMR spectra of starting compounds, photoproducts and photomixtures…………..15

Citation: Mlakić, M.; Perinić, H.; Vušak, V.; Horváth, O.; Sampedro, D.; Losantos, R.; Odak, I.; Škorić, I. Photochemical Transformations of Diverse Biologically Active Resveratrol Analogs in Batch and Flow Reactors. *Molecules* **2024**, *29*, x. https://doi.org/10.3390/xxxxx

Academic Editor: Shengjie Wang

Received: 30 November 2023
Revised: 22 December 2023
Accepted: 27 December 2023
Published: 29 December 2023

Copyright: © 2023 by the authors. Submitted for possible open access publication under the terms and conditions of the Creative Commons Attribution (CC BY) license (https://creativecommons.org/licenses/by/4.0/).

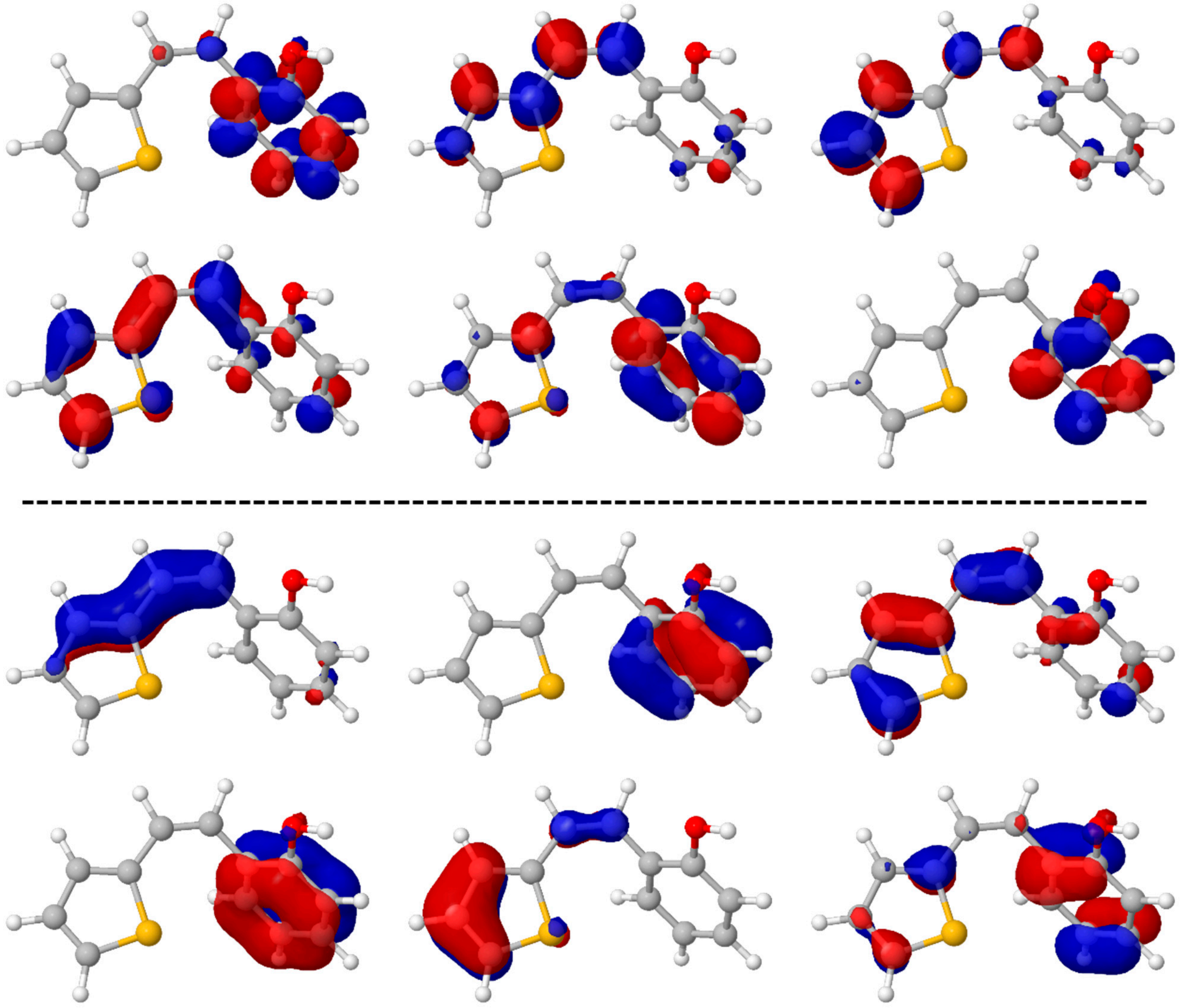

Figure S1. Orbitals included in the active space (12,12).

2. Optimized Geometries coordinates

cis-**1**

C	-2.34912146	-2.83721517	0.32746740
S	-0.69808801	-2.29338239	0.22038612
C	-1.21224600	-0.64319055	-0.11274048
C	-2.58064195	-0.57109337	-0.12386376
C	-3.23484975	-1.82768457	0.12096442
C	-0.34285525	0.52548167	-0.25202138
C	0.99923626	0.64265601	-0.34999265
C	2.02699990	-0.41816300	-0.48403727
C	3.12723392	-0.43174642	0.38950750
C	4.14358887	-1.37314254	0.24758984
C	4.08761555	-2.31327051	-0.78456777
C	3.01645986	-2.30276838	-1.67652697
C	2.00057940	-1.35654550	-1.52318680
O	3.15417780	0.51259682	1.36762991
H	4.97117549	-1.37531072	0.93741124
H	4.87610422	-3.03702732	-0.88682149
H	2.97103020	-3.01388893	-2.48168011
H	1.17984151	-1.33839736	-2.21573463
H	3.92371646	0.40692719	1.90802397
H	1.38959225	1.64464932	-0.32533343
H	-0.89681322	1.44879733	-0.22964683
H	-3.11102387	0.34931551	-0.28742356
H	-4.30158051	-1.95016447	0.14080128
H	-2.56236265	-3.86786269	0.52717946

cis-**1-isom**

C	-3.03300176	-2.36377236	-0.19868236
S	-2.80040865	-0.70493161	-0.66885509
C	-1.11856660	-0.73751505	-0.15332330
C	-0.79903983	-1.97050482	0.34592535
C	-1.89566605	-2.90268143	0.31722412
C	-0.33018305	0.49853045	-0.23870226
C	1.00873996	0.65267975	-0.30963780
C	2.04808998	-0.39213461	-0.46536871
C	3.17279282	-0.38658358	0.37424800
C	4.18900814	-1.32751404	0.21913658
C	4.10464426	-2.28767307	-0.79065029
C	3.00351922	-2.30056957	-1.64608838

C	1.99045107	-1.35560378	-1.48032098
O	3.22104277	0.56806207	1.34180965
H	5.03725393	-1.31328769	0.88298848
H	4.89282132	-3.00979347	-0.90475703
H	2.93726067	-3.02668346	-2.43628862
H	1.14615198	-1.35588344	-2.14432102
H	4.02599863	0.50010467	1.83466310
H	1.38136498	1.65993365	-0.24978848
H	-0.91558287	1.40125817	-0.20882316
H	0.16815575	-2.21365425	0.74115458
H	-1.82185124	-3.91323000	0.67405885
H	-3.98392253	-2.83878883	-0.33232666

trans-**1**

C	1.75412925	2.17949264	0.05833662
C	3.16893397	2.16987005	0.01075835
C	3.88546327	3.33656821	-0.01082022
C	3.23432316	4.57380224	0.02042995
C	1.84983360	4.61749724	0.07873412
C	1.12524489	3.42193895	0.10118830
H	4.96114483	3.29894697	-0.05592030
H	3.81385330	5.47950359	0.00229168
H	1.33007360	5.55824247	0.11520140
H	0.05514598	3.46538889	0.17382041
O	3.76953695	0.95532409	-0.02058462
H	4.71100455	1.05219000	-0.03302007
C	1.02752524	0.89890666	0.07281712
H	1.62290968	0.03693863	0.30811373
C	-0.28824721	0.73760064	-0.19753484
H	-0.88989179	1.58322808	-0.48154357
C	-1.01400577	-0.52267209	-0.16995985
C	-2.32762250	-0.71022342	-0.49986458
S	-0.28479497	-2.08988717	0.31823970
C	-2.77417521	-2.07234230	-0.37950207
H	-2.96572783	0.09267145	-0.82311042
C	-1.79418935	-2.91150973	0.04471124
H	-3.77479011	-2.39052823	-0.60264951
H	-1.86760451	-3.96610388	0.21414542

CI-cycle

C	-0.55067706	-2.50569611	-1.06306979
C	-1.62576676	-2.15316498	0.76701841
C	-1.38831959	-0.91707745	0.01911302
C	-0.23327418	-0.10159040	-0.09679271
C	0.97476989	-0.79270068	-0.36183669
C	0.85390860	-2.09116602	-0.77992953
C	-3.01552521	-2.48023497	0.88059346
C	-3.85438620	-1.60527816	0.27216011
S	-2.96585357	-0.29838792	-0.54556673
C	-0.79501238	-3.96723224	-1.17245655
C	0.24605936	-4.81783943	-1.21251017
C	1.64051146	-4.39198013	-1.03054120
C	1.91042319	-3.08573027	-0.81825213
O	3.15350375	-2.59145726	-0.59680534
H	2.42235058	-5.13017501	-1.05212349
H	0.06258366	-5.86319367	-1.38847790
H	-1.80202599	-4.30784118	-1.32085902
H	-0.99052586	-1.97343643	-1.88912684
H	3.78887351	-3.29400559	-0.59523213
H	1.93379413	-0.31691379	-0.26660688
H	-0.34309129	0.94677959	-0.29869543
H	-0.85949889	-2.58090820	1.37965071
H	-3.35844232	-3.33441591	1.43432859
H	-4.92448884	-1.61025179	0.26475724

1-MinS$_1$

C	-1.64400900	-0.14677500	0.00051700
C	-2.57300000	0.94738100	0.00200100
C	-3.94516000	0.73983600	0.00060600
C	-4.45426500	-0.56032700	-0.00261900
C	-3.57897800	-1.65289700	-0.00459900
C	-2.21136700	-1.45842500	-0.00317200
H	-4.61928900	1.59413300	0.00196600
H	-5.52844400	-0.71723300	-0.00365900
H	-3.97895300	-2.66248900	-0.00740700
H	-1.55090700	-2.31904000	-0.00548300
O	-2.03478100	2.18779000	0.00500900
H	-2.74344200	2.84359200	0.00595800
C	-0.25439200	0.11403600	0.00266700
H	0.04739100	1.15793200	0.00389300

C	0.74920400	-0.86221800	0.00428600
H	0.48586600	-1.91695100	0.00758300
C	2.11940800	-0.57376400	0.00255100
C	3.21954000	-1.45466300	0.00547600
S	2.70109100	1.10837400	-0.00505600
C	4.45152600	-0.79458800	0.00163400
H	3.09473600	-2.53140000	0.01033000
C	4.33848600	0.58654700	-0.00399800
H	5.41029400	-1.30088200	0.00310900
H	5.14158600	1.31118400	-0.00756100

ISC T_2S_1

C	1.99942900	1.59866600	-0.88705400
C	3.38962700	1.55085800	-0.56925600
C	4.25396200	2.58351400	-0.92716800
C	3.76998400	3.69355600	-1.60995900
C	2.42231800	3.77810300	-1.93853800
C	1.55196900	2.75724300	-1.58806500
H	5.30926700	2.51592400	-0.66920300
H	4.45230300	4.49352800	-1.88443600
H	2.04620800	4.64582500	-2.47177100
H	0.50339700	2.84131000	-1.85400900
O	3.82386500	0.45534300	0.09537300
H	4.77265100	0.53281900	0.25576000
C	1.16011400	0.54288300	-0.50999000
H	1.60374600	-0.29363400	0.02281700
C	-0.24386900	0.50432800	-0.78119300
H	-0.71770500	1.33136400	-1.30254700
C	-1.05170800	-0.53765400	-0.41288300
C	-2.43483300	-0.74536300	-0.58987900
S	-0.35718500	-1.94239400	0.44949700
C	-2.87147900	-1.96724800	-0.05335500
H	-3.06887000	-0.02264000	-1.08979100
C	-1.87713900	-2.70912300	0.53078300
H	-3.90167200	-2.30370100	-0.09170900
H	-1.97686200	-3.67622200	1.00646200

IC T_1 T_2

C	1.98880900	1.65093700	-0.91693700
C	3.34864200	1.57853300	-0.59264600
C	4.34800300	2.69730600	-0.96526900
C	3.87055000	3.79169000	-1.63599700

C	2.51090900	3.88049100	-1.96867500
C	1.52641800	2.75778400	-1.59141700
H	5.39380900	2.58920800	-0.68822600
H	4.53563600	4.60326400	-1.91852900
H	2.13043100	4.74995400	-2.50148200
H	0.48683400	2.87641800	-1.87302300
O	3.84782800	0.51527100	0.06649800
H	4.79944000	0.63181600	0.20561800
C	1.12767100	0.50535900	-0.50139000
H	1.64706500	-0.30026600	0.02644300
C	-0.16586700	0.40872100	-0.72201700
H	-0.69092100	1.20365000	-1.24522100
C	-1.04969900	-0.71326500	-0.32384700
C	-2.38419300	-0.80187100	-0.55404600
S	-0.48591900	-2.11944100	0.52077200
C	-2.98070800	-2.00960100	-0.05203000
H	-2.94184300	-0.02582000	-1.07122300
C	-2.06108900	-2.81021400	0.55271700
H	-4.02416500	-2.25681600	-0.13989600
H	-2.22012000	-3.77582800	1.01971100

ISC T_1 S_0 66º

C	1.91753000	1.60447500	-0.29055200
C	3.33155400	1.78249600	-0.18965200
C	4.06622400	2.29876600	-1.24508900
C	3.43127300	2.66102100	-2.43488700
C	2.04997100	2.50370700	-2.56555100
C	1.31072700	1.98877400	-1.51632500
H	5.14231900	2.42484400	-1.14021500
H	4.01861600	3.06743200	-3.25178400
H	1.55338400	2.78720600	-3.48828600
H	0.23548800	1.87680600	-1.61142200
O	3.90703700	1.42036400	0.98726000
H	4.85795800	1.57078100	0.93248200
C	1.19233500	1.07849000	0.79910100
H	1.73517200	0.89879100	1.72380200
C	-0.24088100	0.78858700	0.79697700
H	-0.87126600	1.32223200	1.50924900
C	-0.87067700	-0.17999100	0.03226600
C	-2.24251100	-0.51822600	0.02962400
S	-0.00435500	-1.23433100	-1.08794000
C	-2.55527800	-1.58097800	-0.83148900

H	-2.96116900	0.00351700	0.65111200
C	-1.45911800	-2.07236400	-1.50246400
H	-3.55410200	-1.98377400	-0.95645500
H	-1.43271100	-2.88134300	-2.21987500

ISC T_1 S_0 122º

C	1.97074100	1.64885900	-0.51656300
C	3.37843400	1.71403900	-0.28079300
C	4.20715900	2.46976900	-1.09508400
C	3.67792900	3.18266900	-2.17217700
C	2.30803900	3.13007100	-2.43875500
C	1.47499000	2.37914100	-1.62909100
H	5.27568300	2.50553800	-0.89032100
H	4.33860600	3.77575400	-2.79658500
H	1.89733300	3.67818600	-3.28068200
H	0.41029000	2.33100900	-1.83857700
O	3.85112500	1.00433300	0.77705200
H	4.81051400	1.09669400	0.81623800
C	1.13649300	0.88985900	0.32768500
H	1.59738500	0.34309200	1.14731400
C	-0.32135900	0.81762800	0.18175400
H	-0.91702900	1.71813800	0.33741700
C	-1.00596300	-0.34870400	-0.10647500
C	-2.40028300	-0.54807800	-0.21493900
S	-0.16754800	-1.87285300	-0.40625500
C	-2.75157500	-1.87098500	-0.52600000
H	-3.10892400	0.25804300	-0.06456100
C	-1.66448500	-2.70339300	-0.65908900
H	-3.77317800	-2.21209900	-0.64878500
H	-1.66685500	-3.75942400	-0.89284200

3. Spectral series recorded during the direct irradiations of the various *cis*-isomers in time at 313 and 365 nm

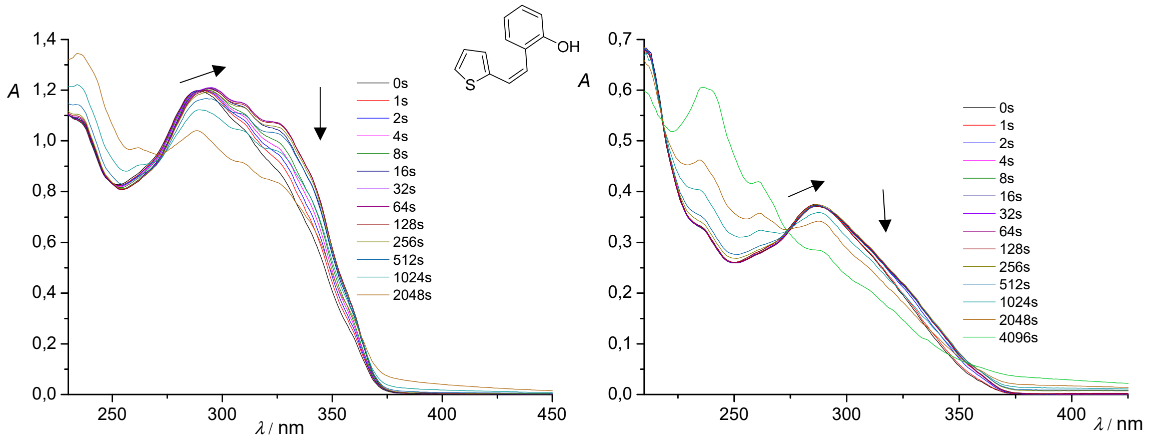

Figure S2. Compound *cis*-**1**: 300 nm (left); 350 nm (right).

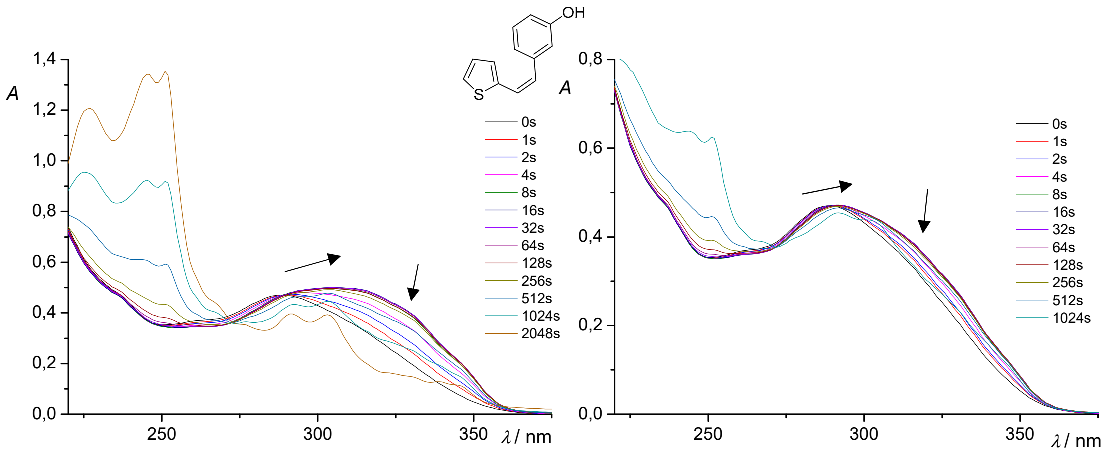

Figure S3. Compound *cis*-**2**: 300 nm (left); 350 nm (right).

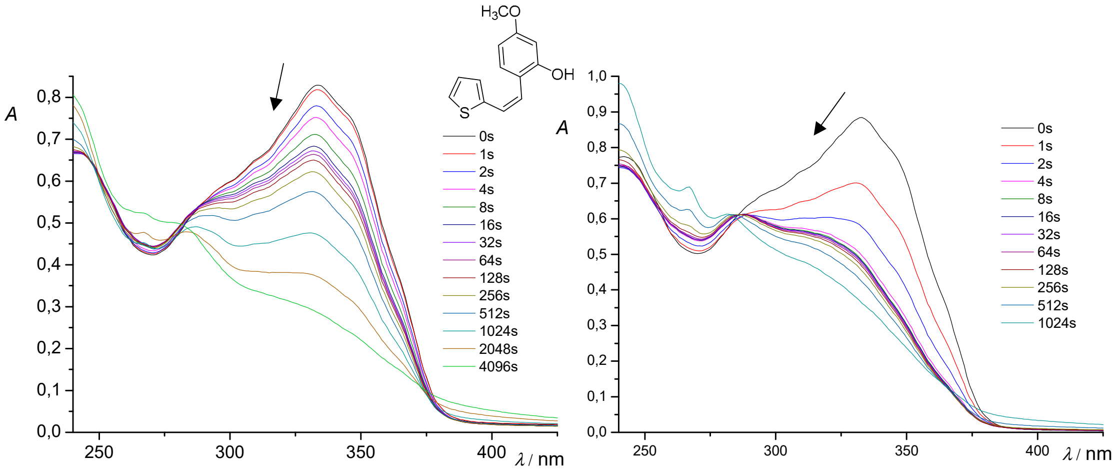

Figure S4. Compound *cis*-**3**: 300 nm (left); 350 nm (right).

4. Chromatograms for data in Table 8 - Most successful photochemical reactions of the starting compounds 1-5 using performed in a flow reactor (followed by HPLC at 320 nm).

Chromatogram for compound **1** and flow reactor (τ = 2′) using LED lamp of 365 nm without photocatalyst:

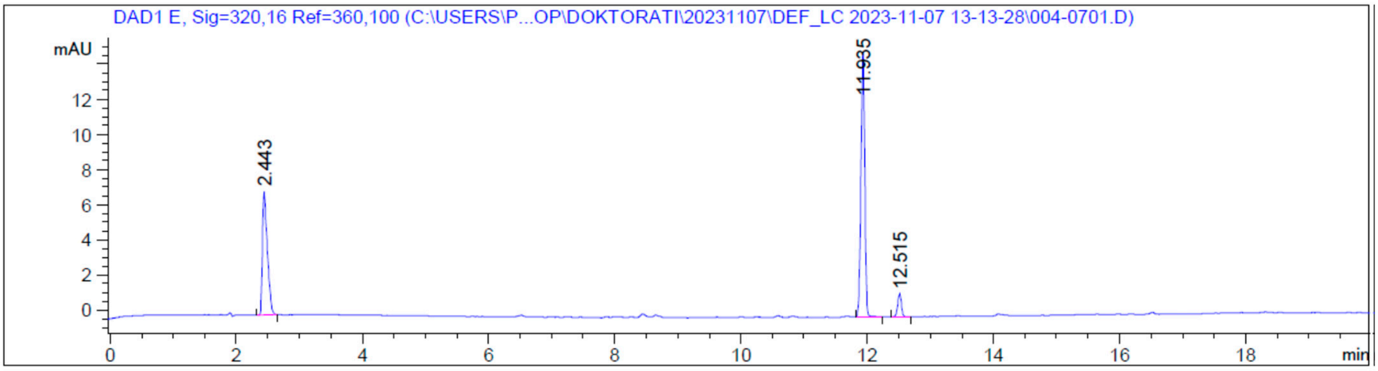

Chromatogram for compound **2** and flow reactor (τ = 2′) using LED lamp of 365 nm without photocatalyst:

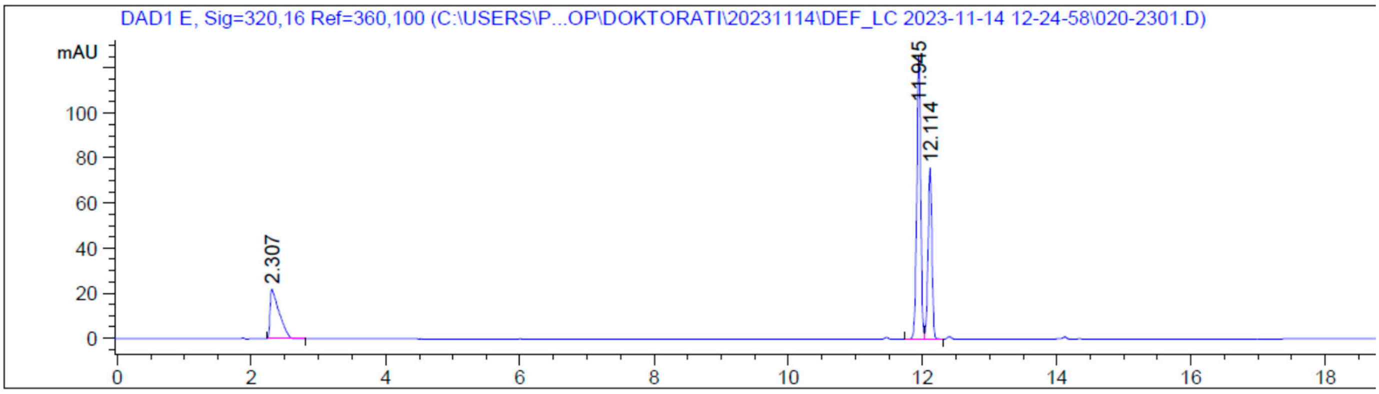

Chromatogram for compound **3** and flow reactor (τ = 2′) using LED lamp of 365 nm without photocatalyst:

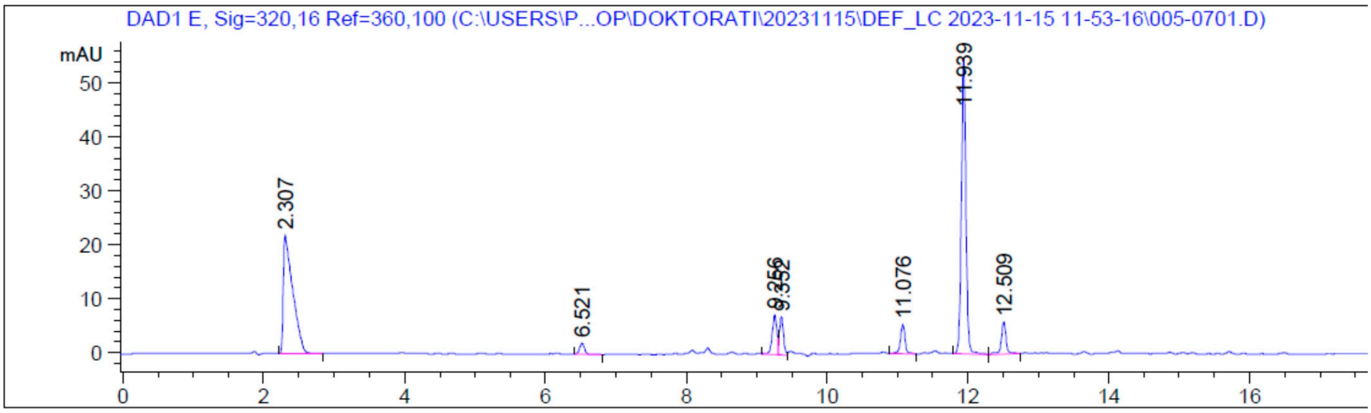

Chromatogram for compound **4** and flow reactor (τ = 2′) using LED lamp of 365 nm without photocatalyst:

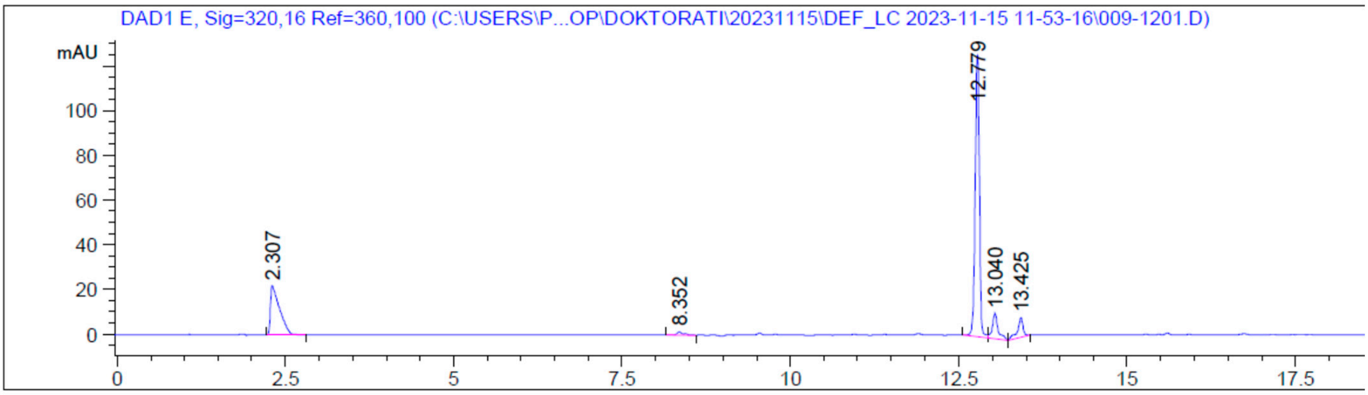

Chromatogram for compound **5** and flow reactor (τ = 2′) using LED lamp of 365 nm without photocatalyst:

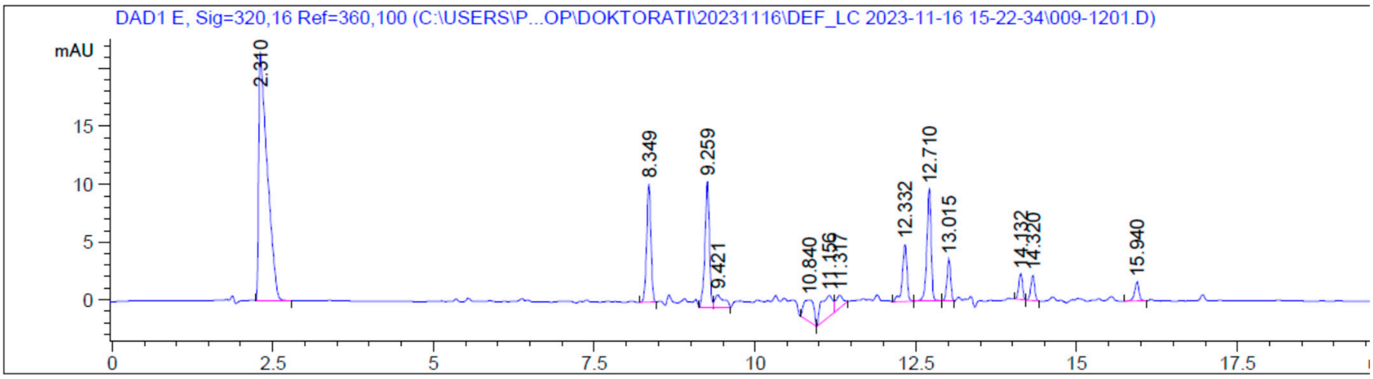

Chromatogram for compound **1** and flow reactor (τ = 10′) using LED lamp of 420 nm with photocatalyst free-base anionic porphyrin:

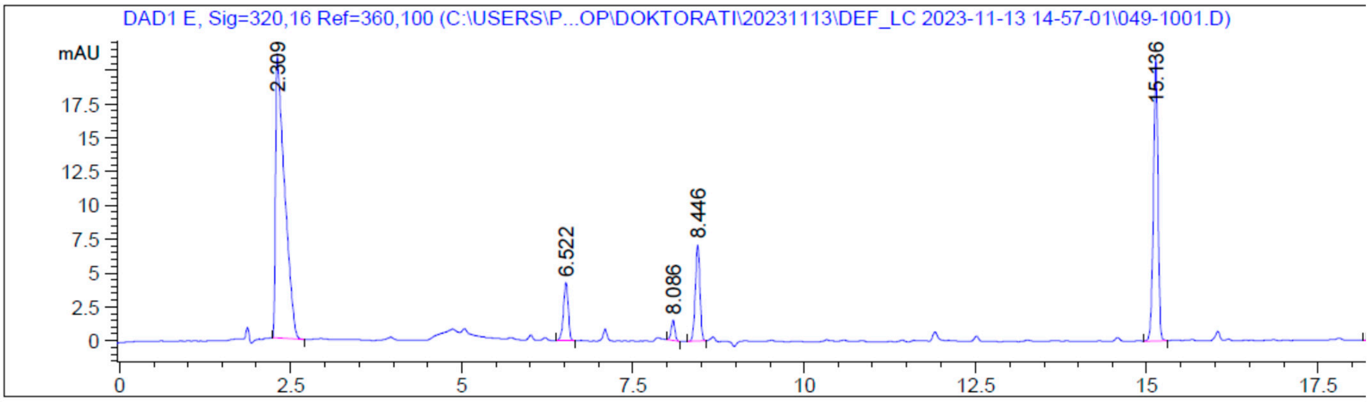

Chromatogram for compound **2** and flow reactor (τ = 10′) using LED lamp of 420 nm with photocatalyst free-base anionic porphyrin:

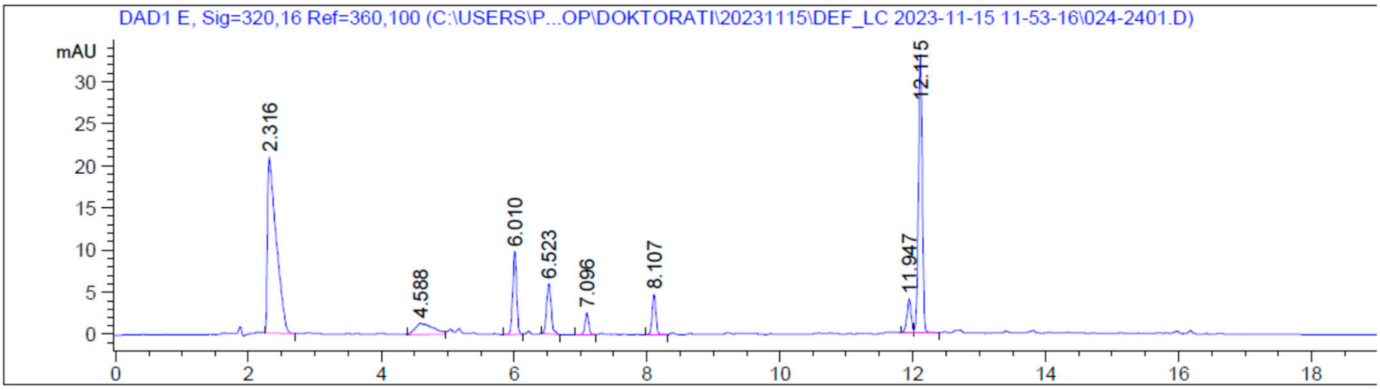

Chromatogram for compound **3** and flow reactor (τ = 10′) using LED lamp of 420 nm with photocatalyst free-base anionic porphyrin:

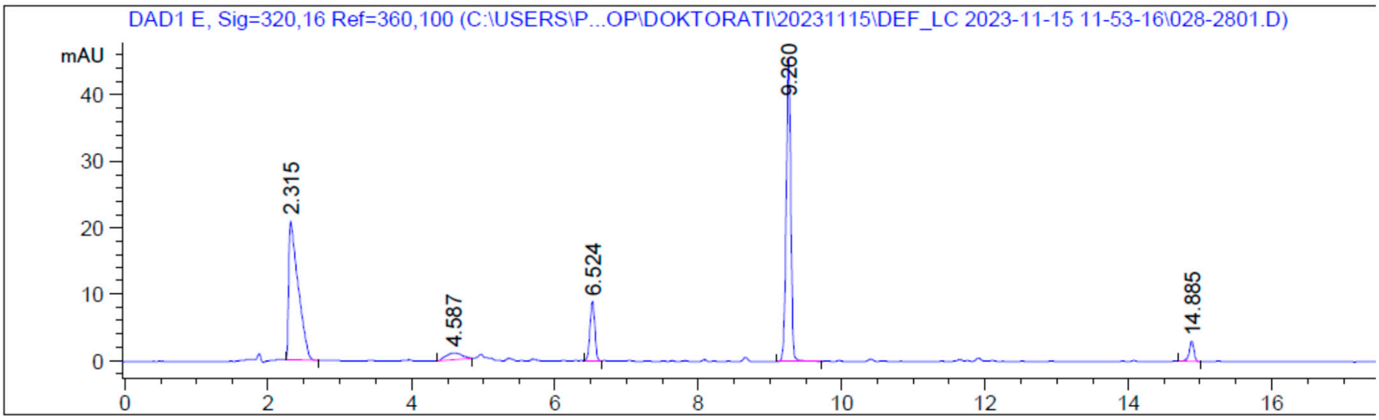

Chromatogram for compound **4** and flow reactor (τ = 10′) using LED lamp of 420 nm with photocatalyst free-base anionic porphyrin:

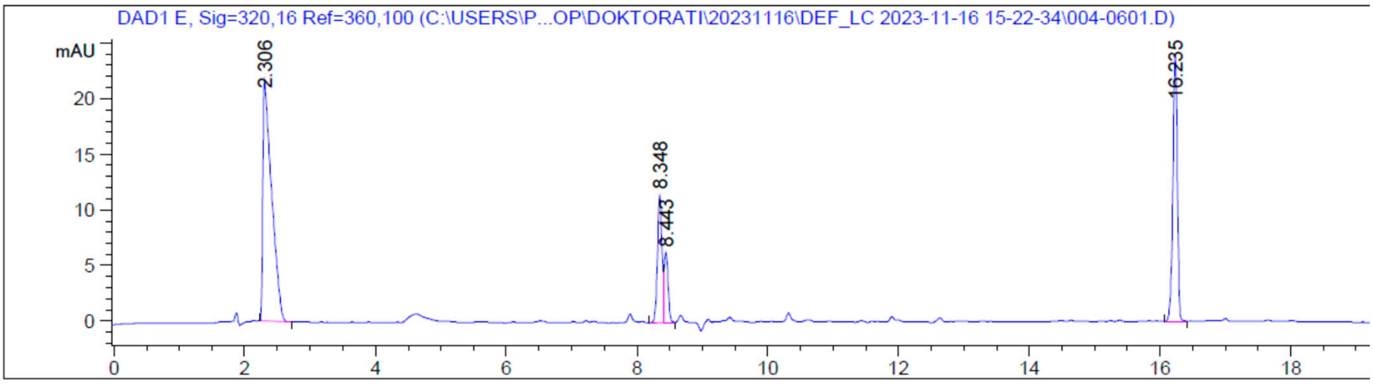

Chromatogram for compound **5** and flow reactor (τ = 10′) using LED lamp of 420 nm with photocatalyst free-base anionic porphyrin:

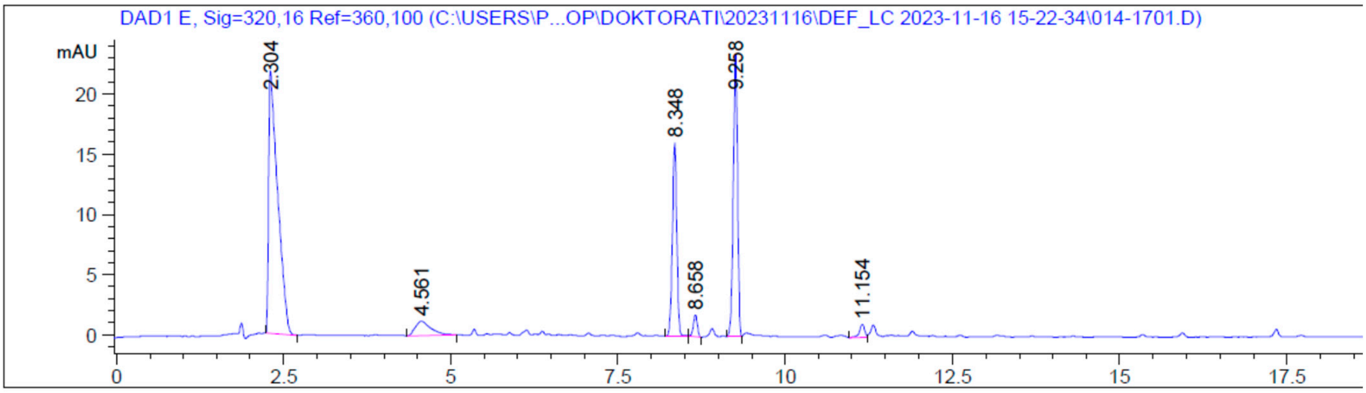

5. NMR spectra of starting compounds, photoproducts and photomixtures

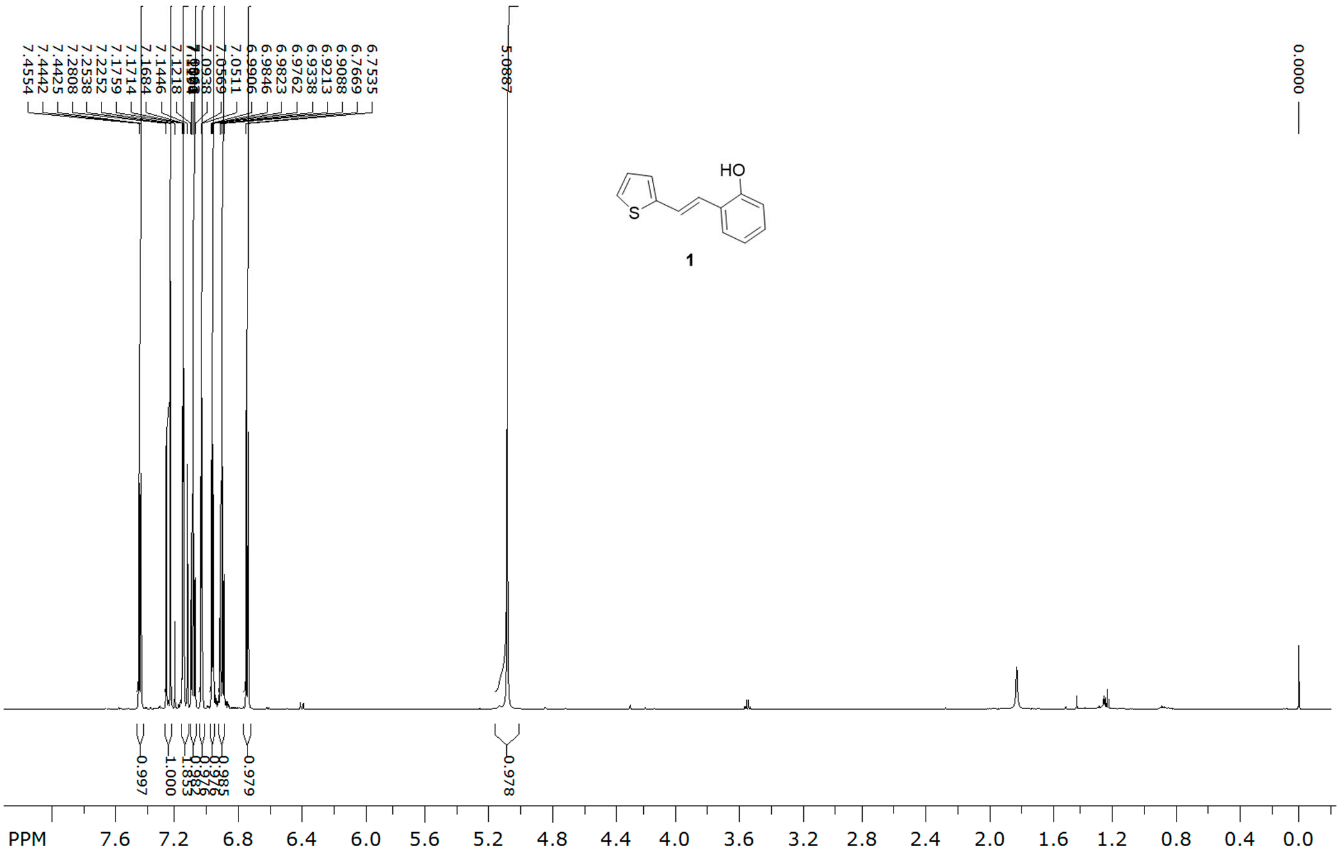

Figure S5. ^{1}H NMR ($CDCl_3$) spectrum of **1**.

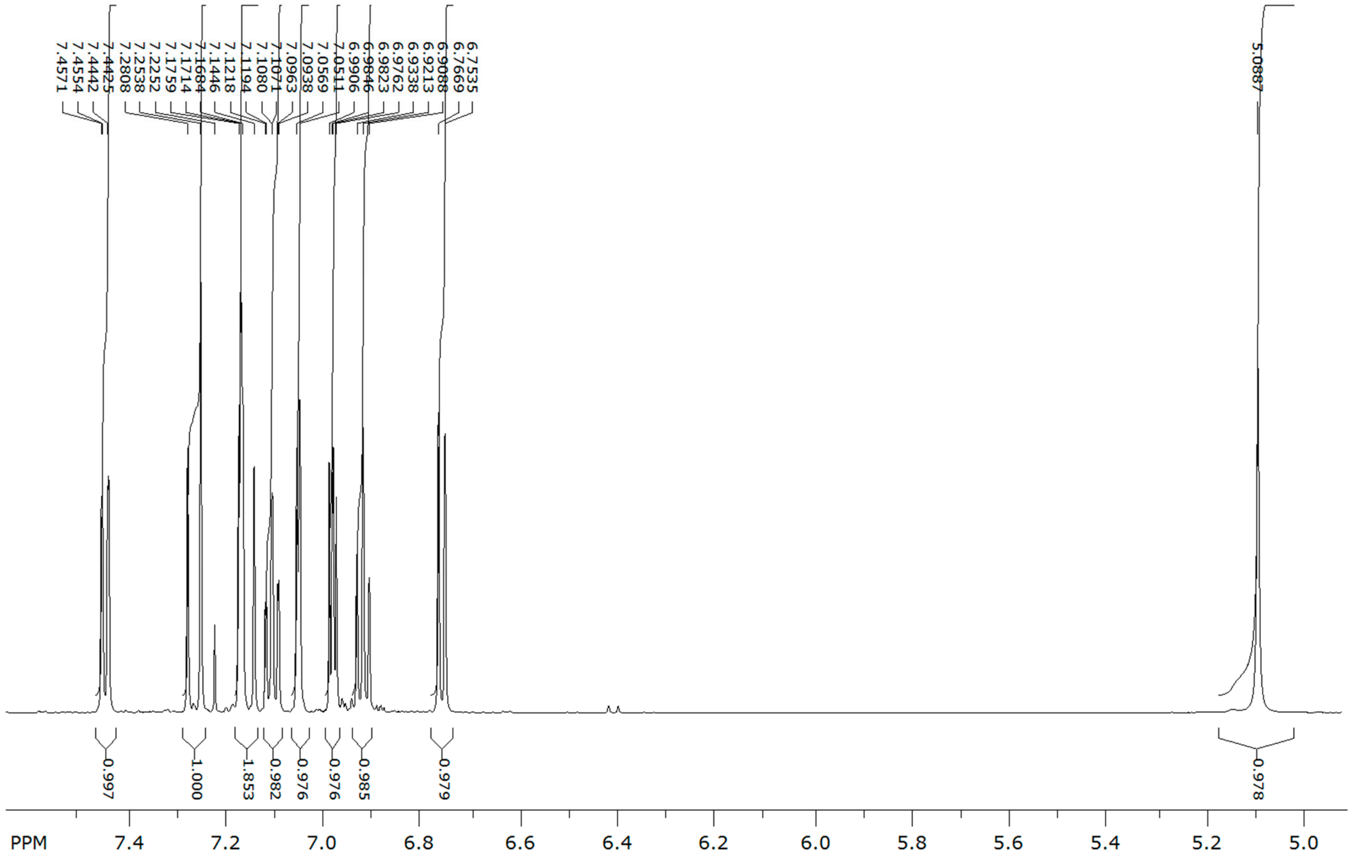

Figure S6. ^{1}H NMR ($CDCl_3$) spectrum of aromatic part of **1**.

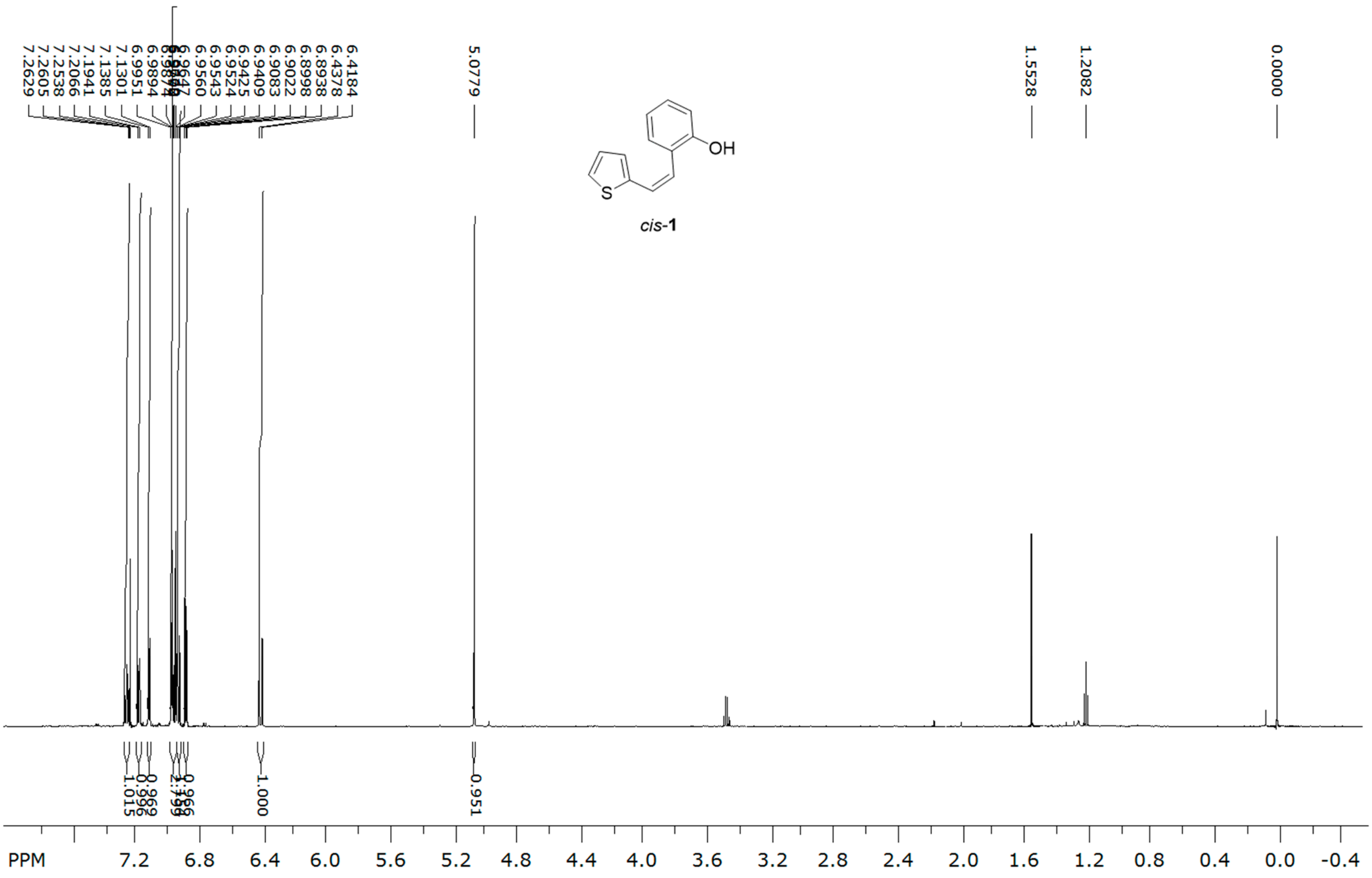

Figure S7. ^{1}H NMR ($CDCl_3$) spectrum of *cis*-**1**.

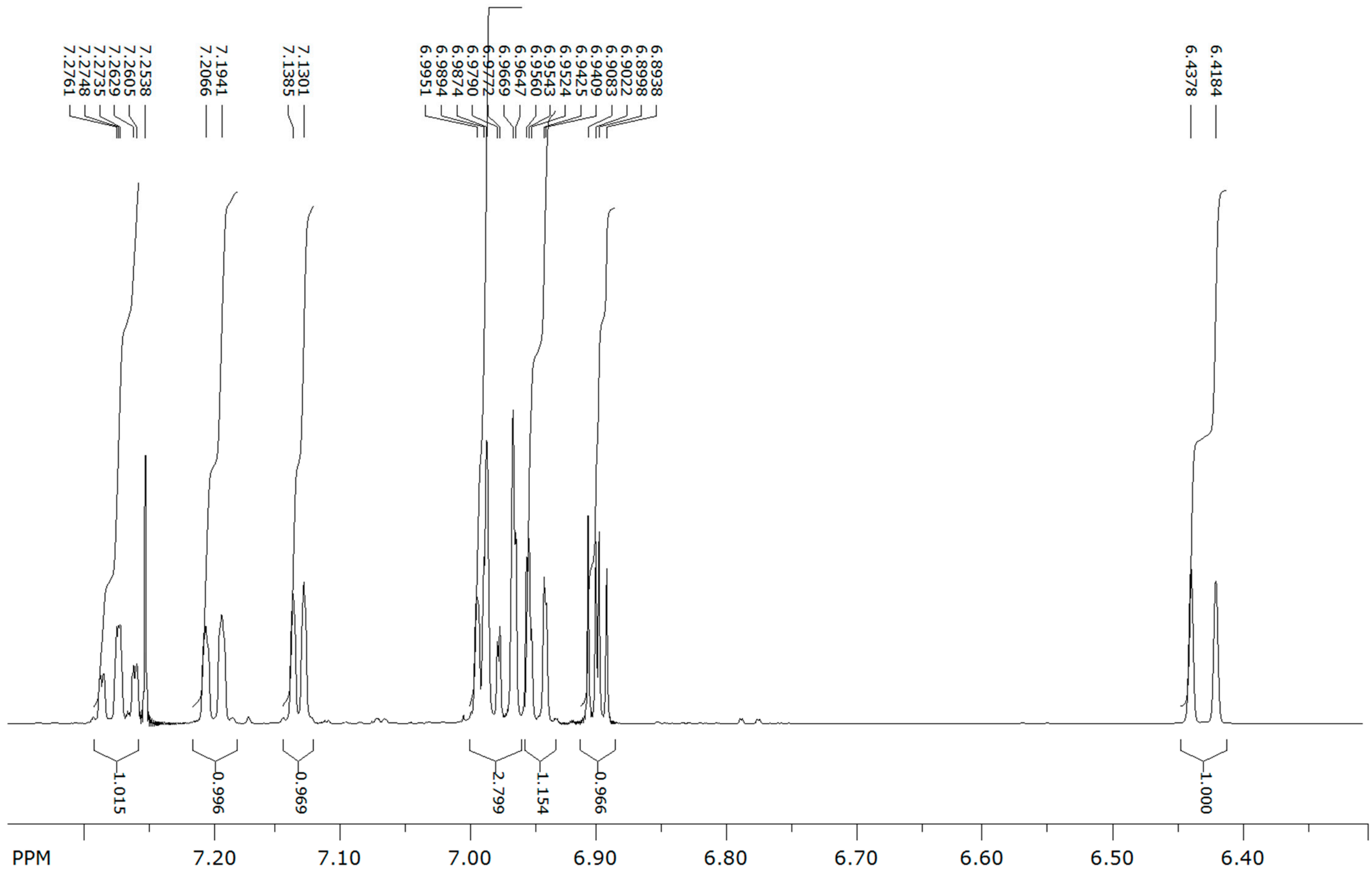

Figure S8. ^{1}H NMR ($CDCl_3$) spectrum of aromatic part of *cis*-**1**.

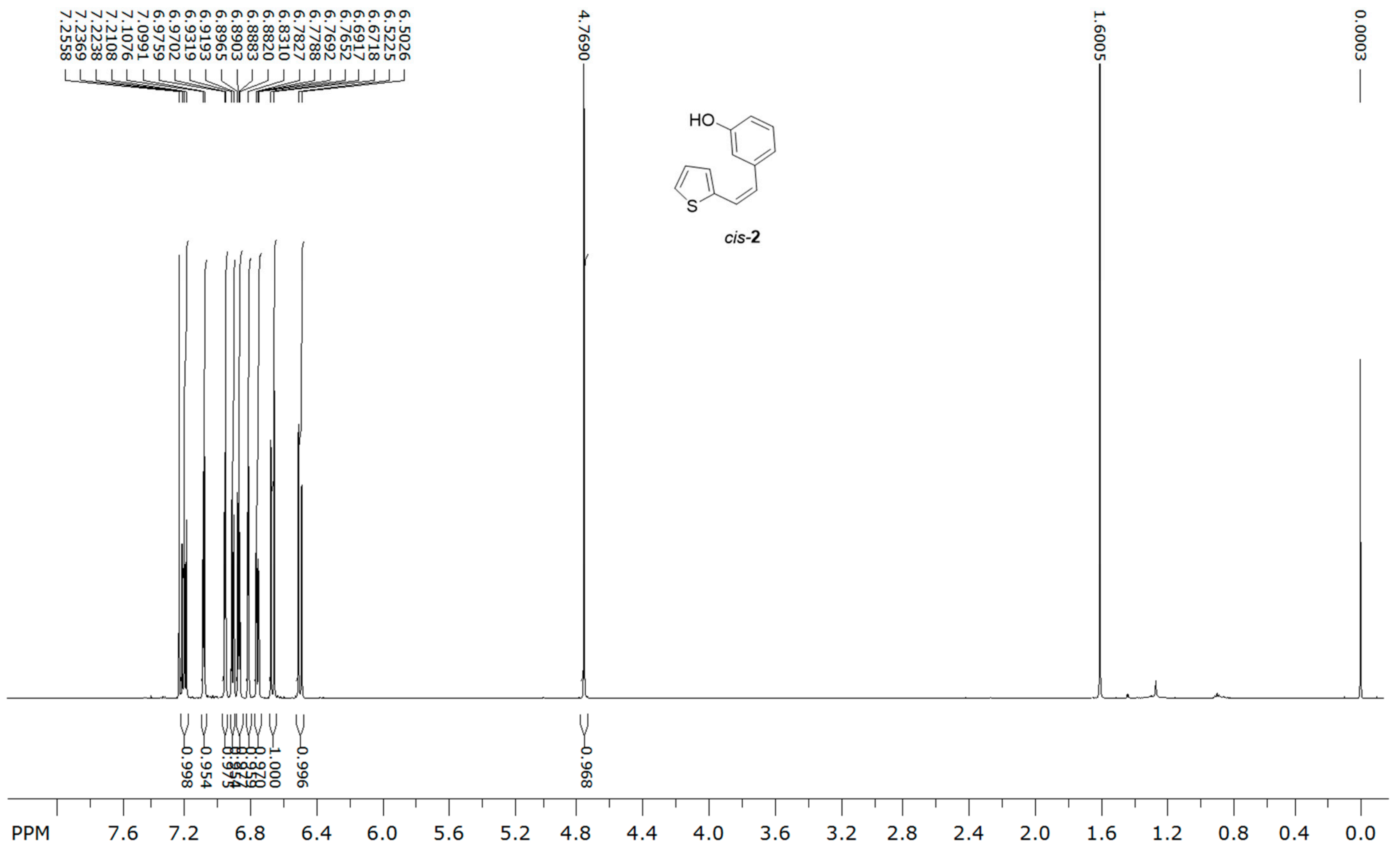

Figure S9. ^{1}H NMR ($CDCl_3$) spectrum of *cis*-**2**.

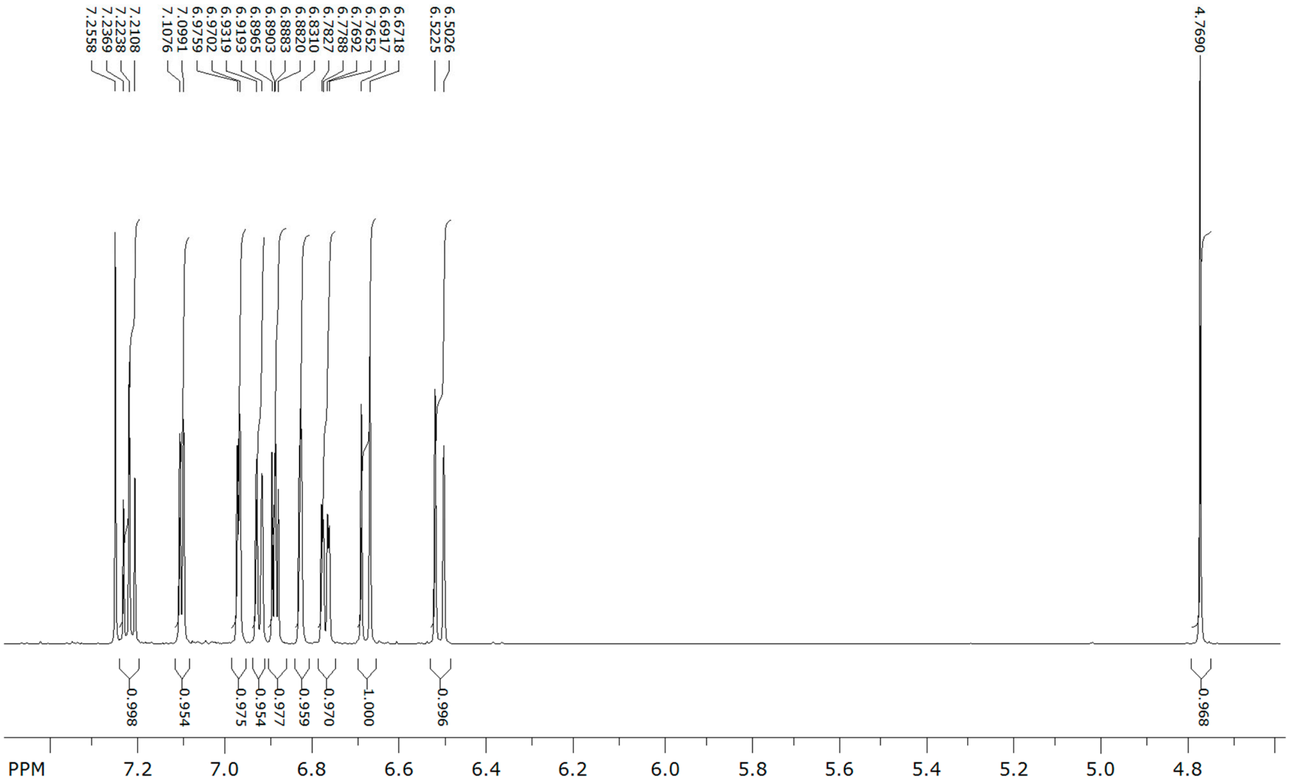

Figure S10. ^1H NMR ($CDCl_3$) spectrum of aromatic part of *cis*-**2**.

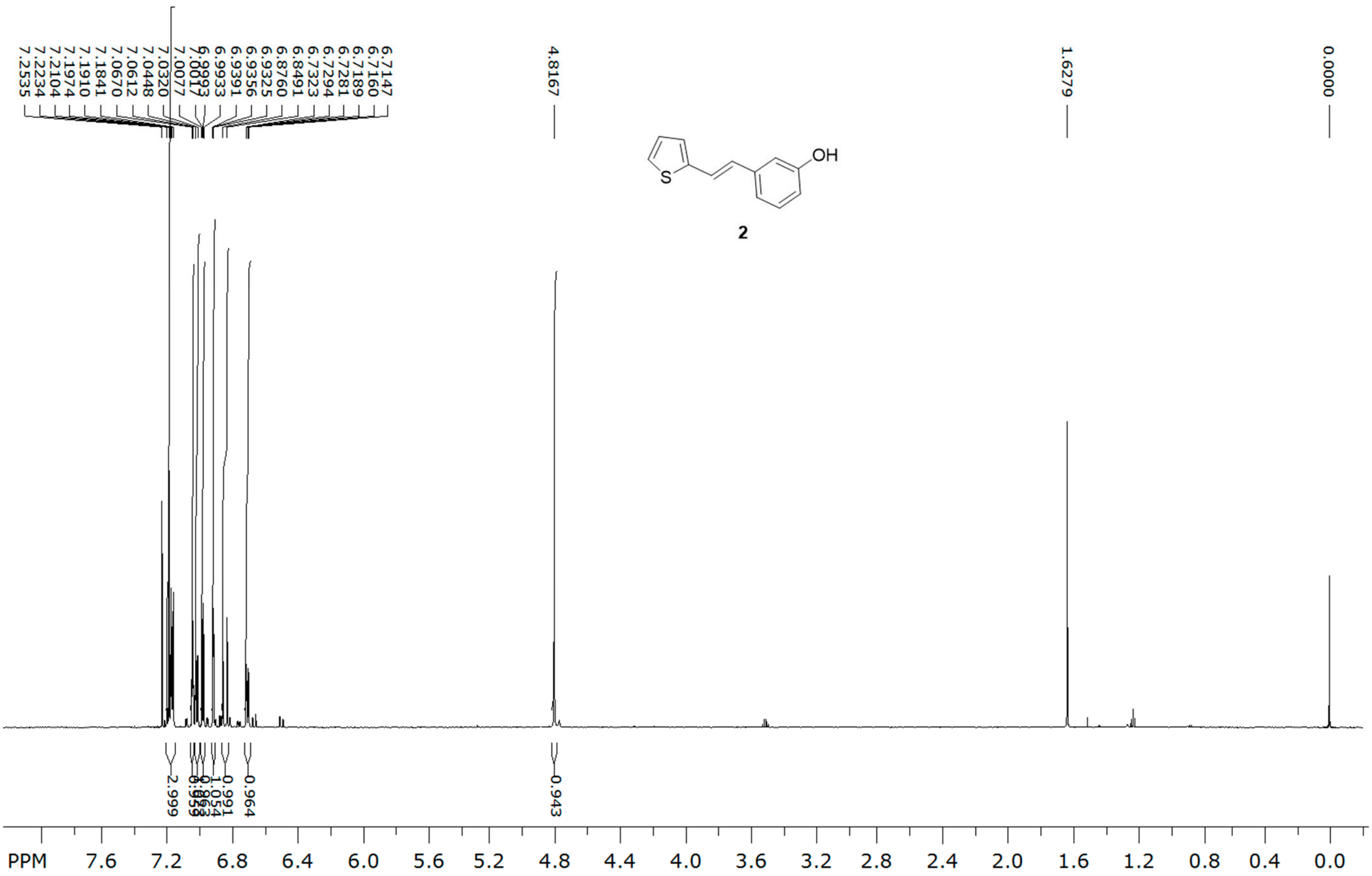

Figure S11. ^{1}H NMR ($CDCl_3$) spectrum of **2**.

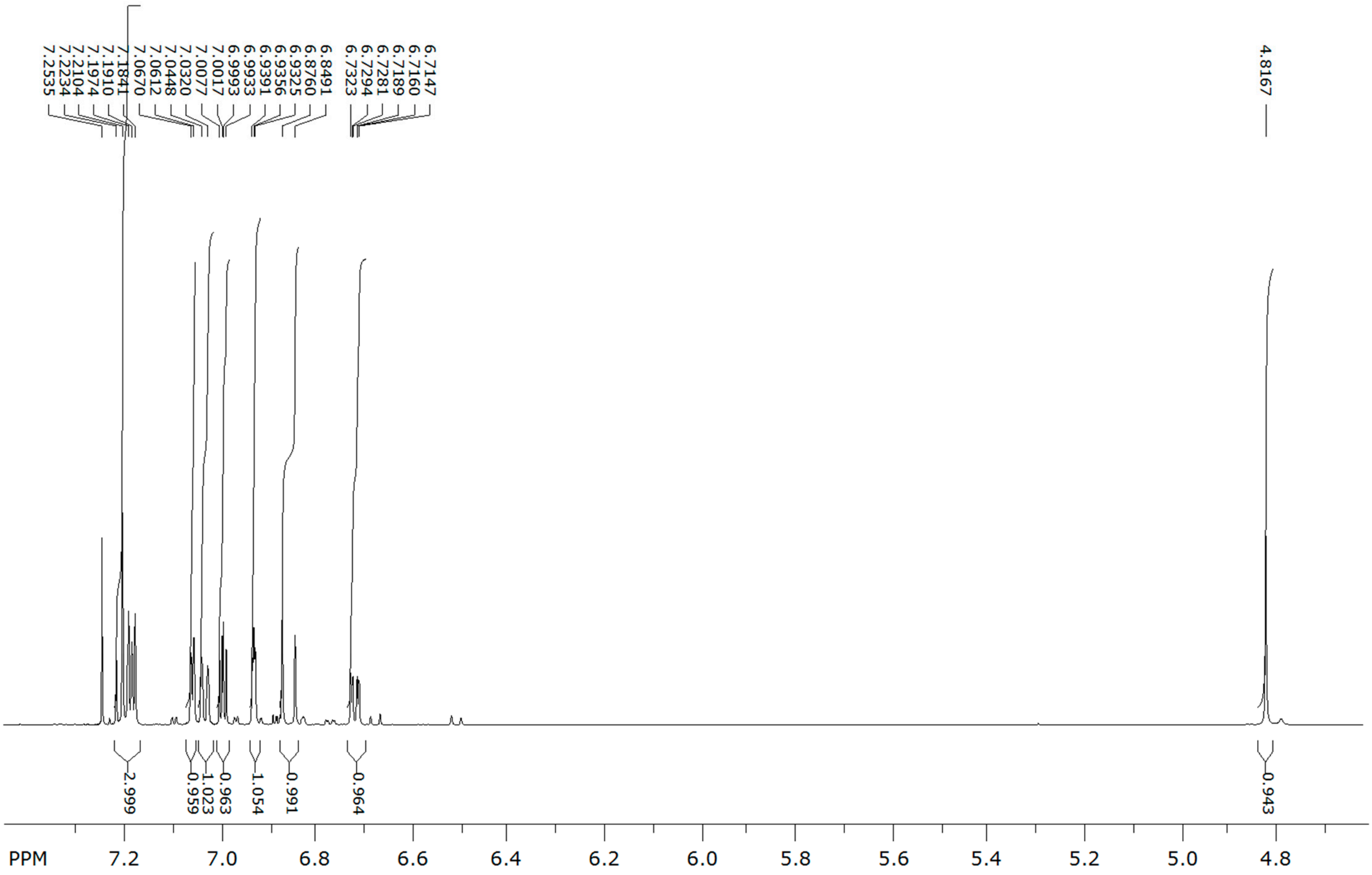

Figure S12. ^{1}H NMR ($CDCl_3$) spectrum of aromatic part of **2**.

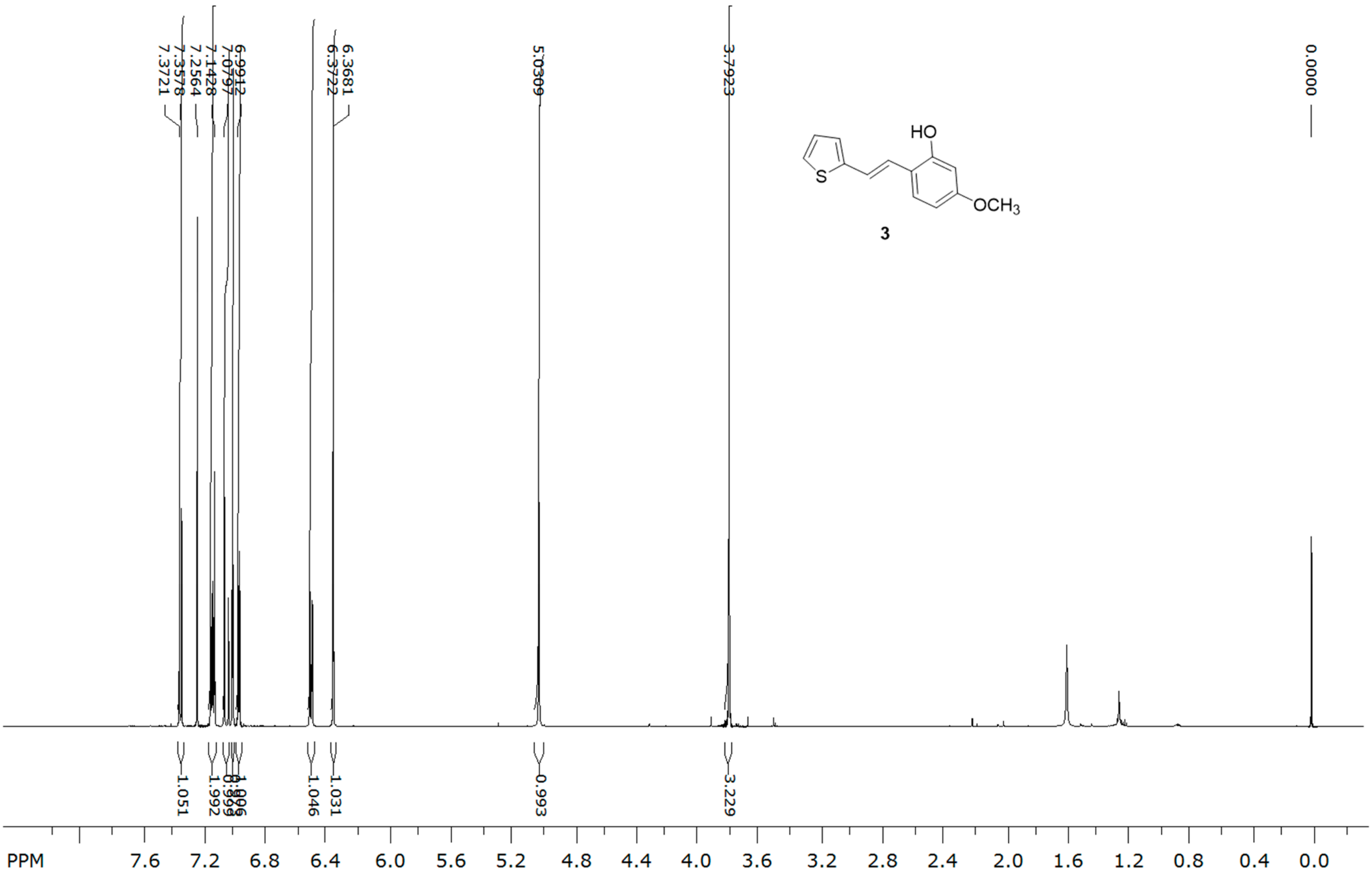

Figure S13. ^{1}H NMR ($CDCl_3$) spectrum of **3**.

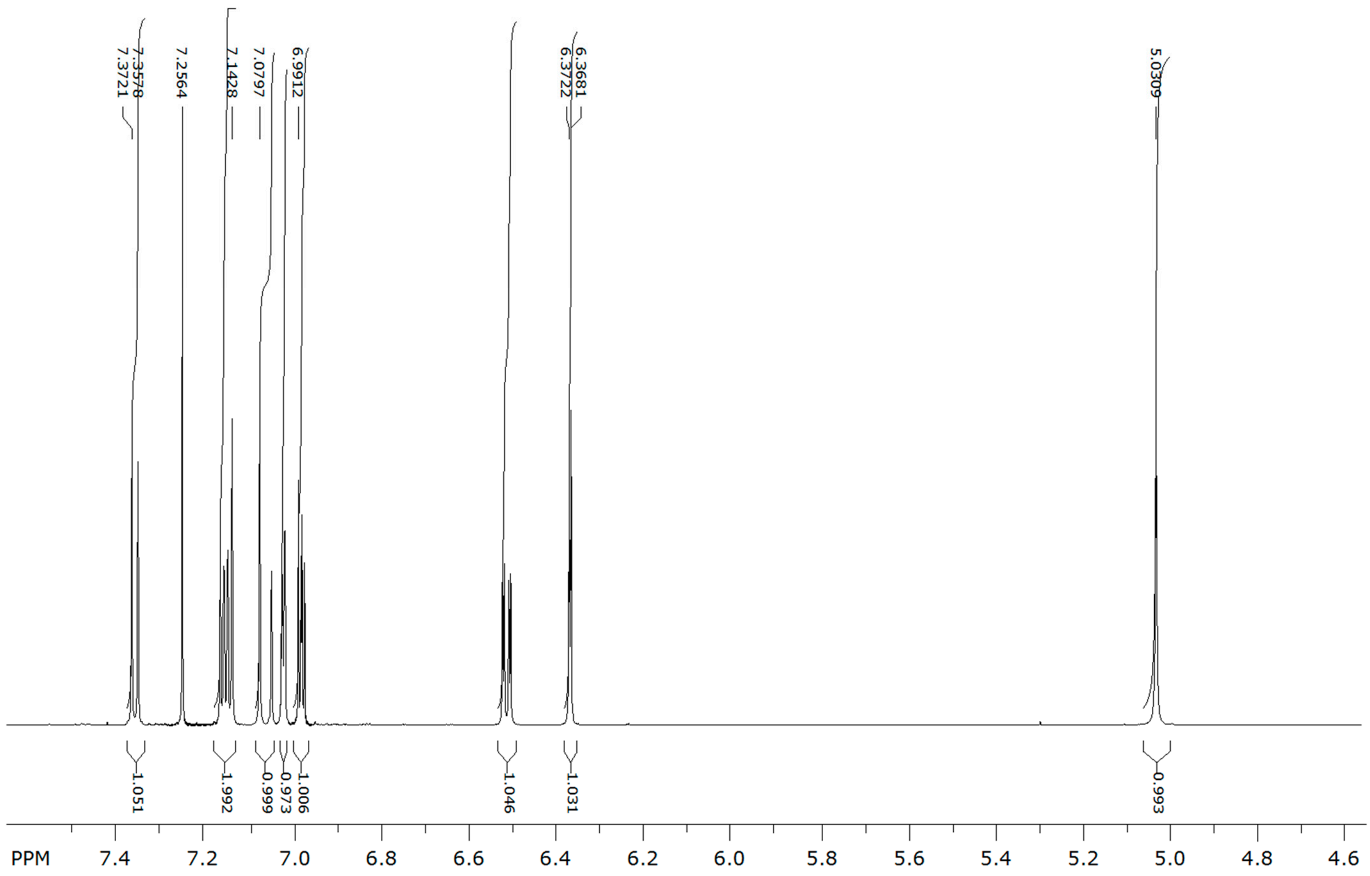

Figure S14. ^1H NMR ($CDCl_3$) spectrum of aromatic part of **3**.

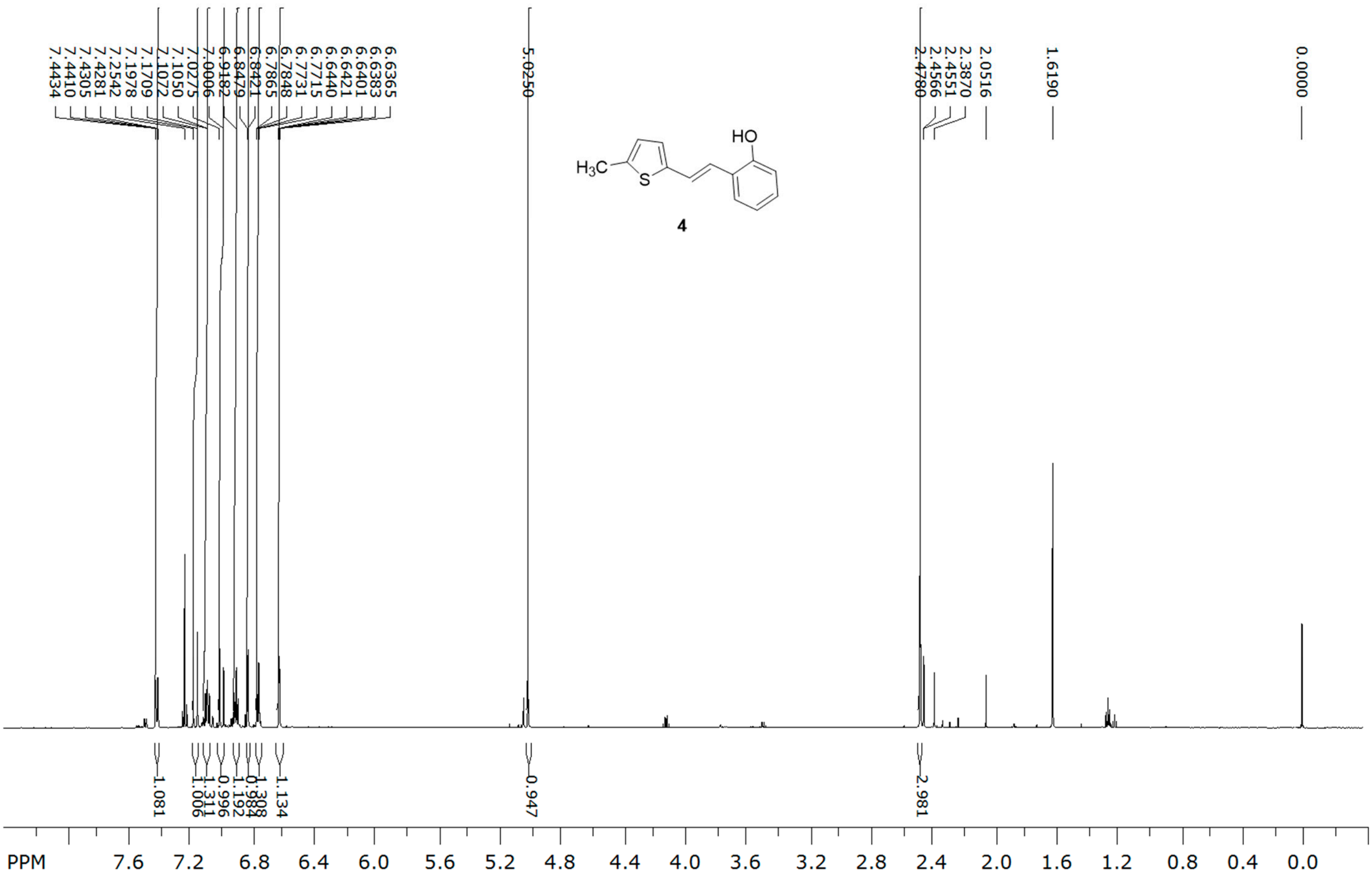

Figure S15. ^{1}H NMR ($CDCl_3$) spectrum of **4**.

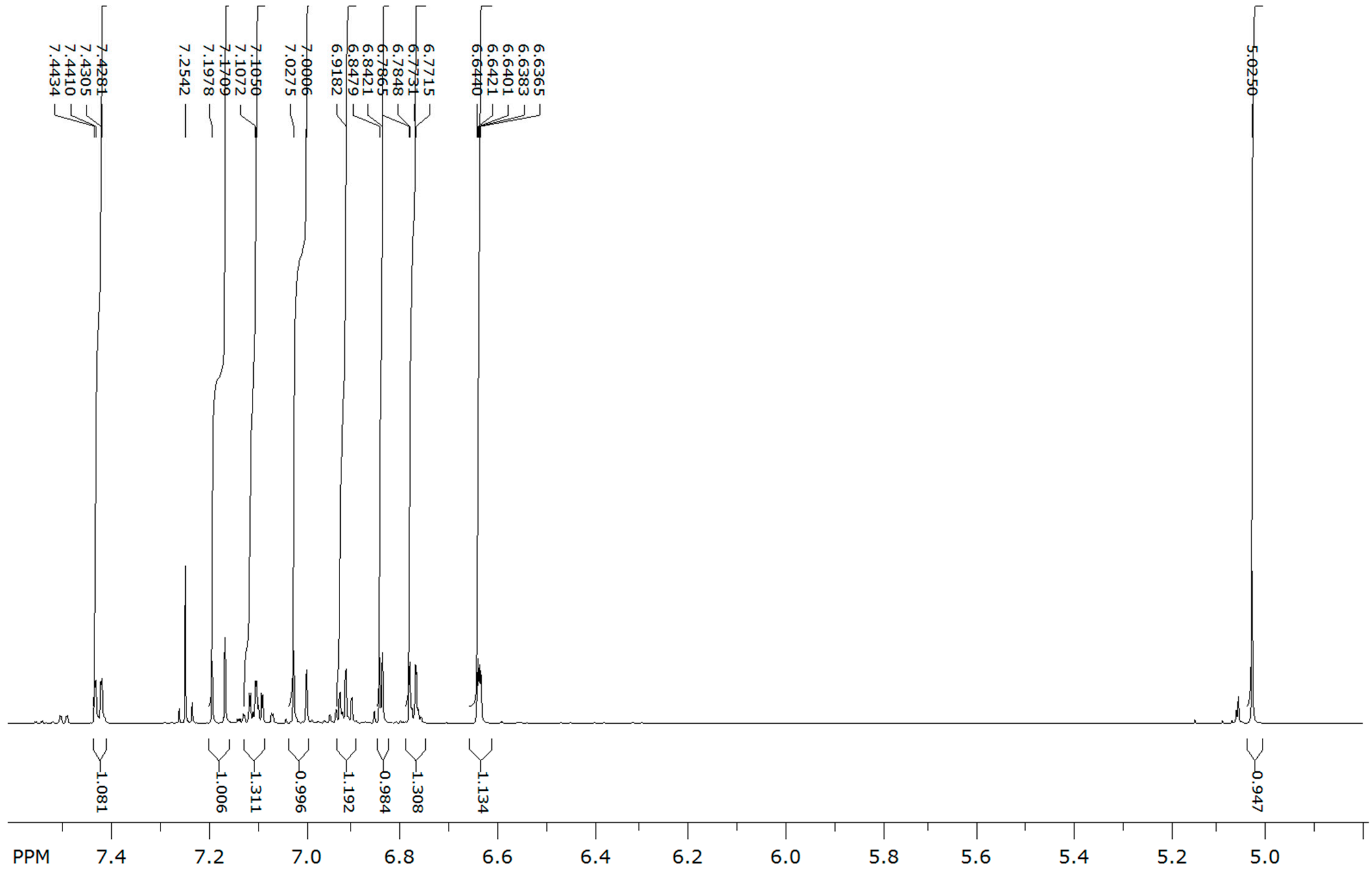

Figure S16. ^{1}H NMR ($CDCl_3$) spectrum of aromatic part of **4**.

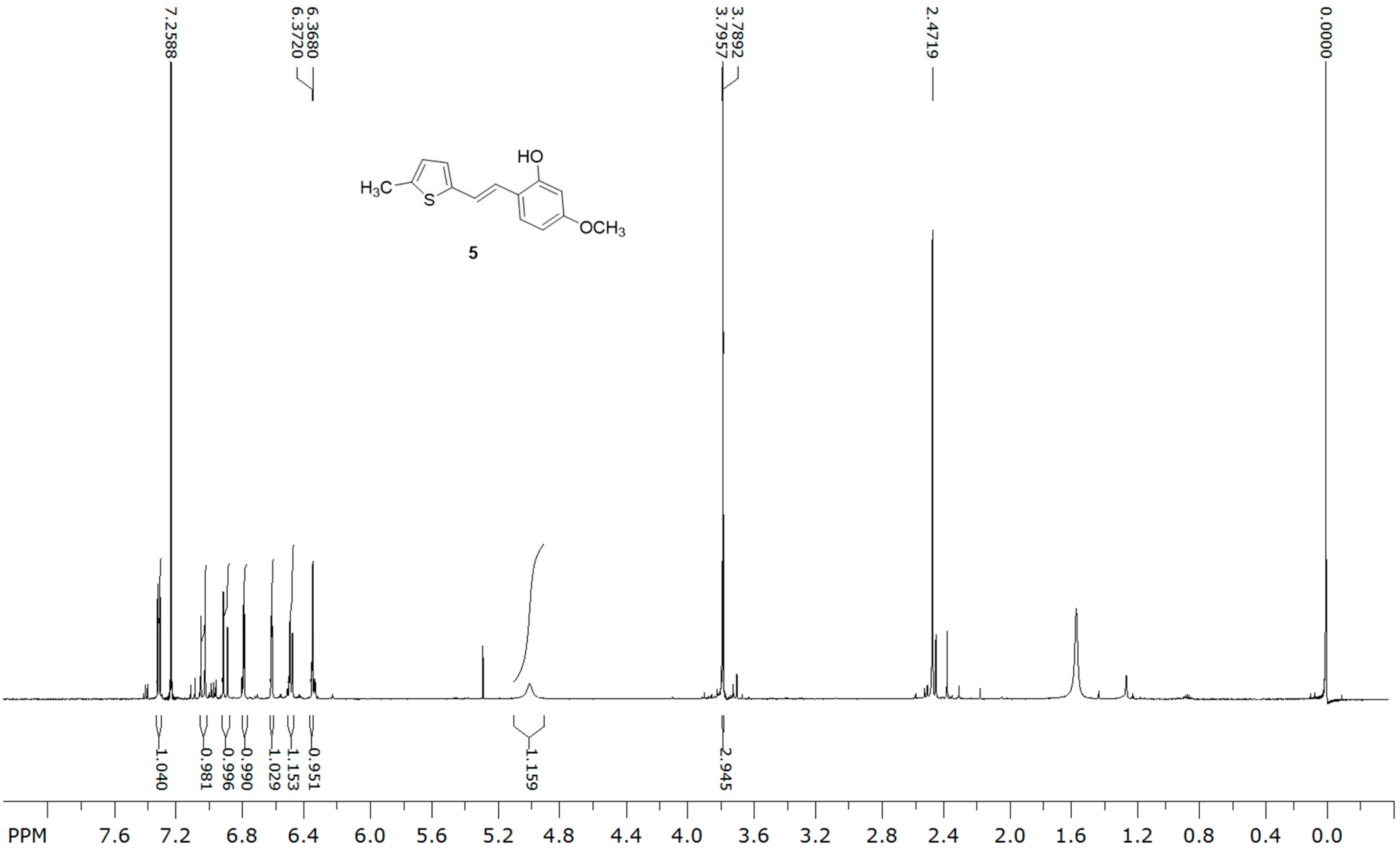

Figure S17. ^{1}H NMR ($CDCl_3$) spectrum of **5**.

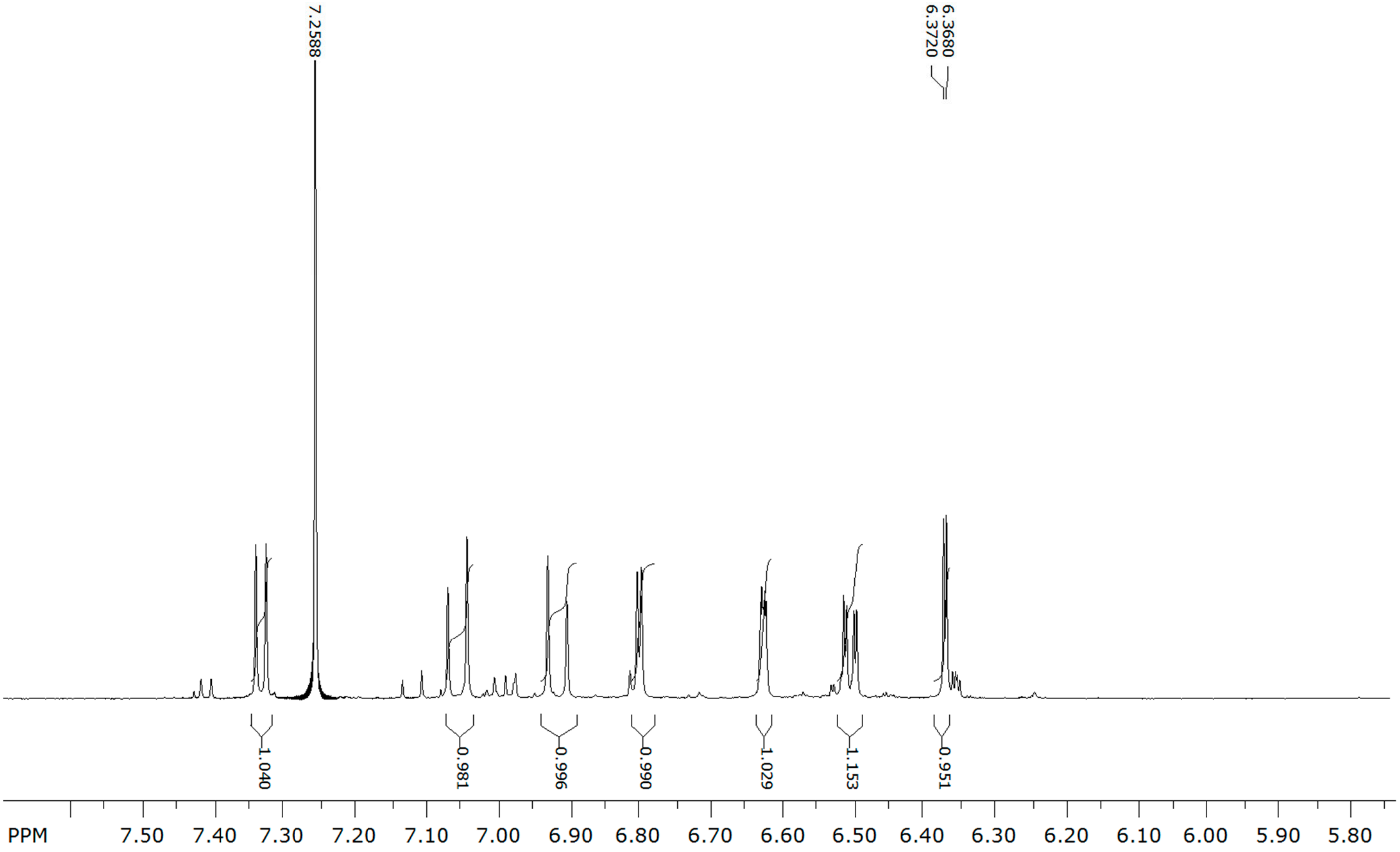

Figure S18. ^{1}H NMR ($CDCl_3$) spectrum of aromatic part of **5**.

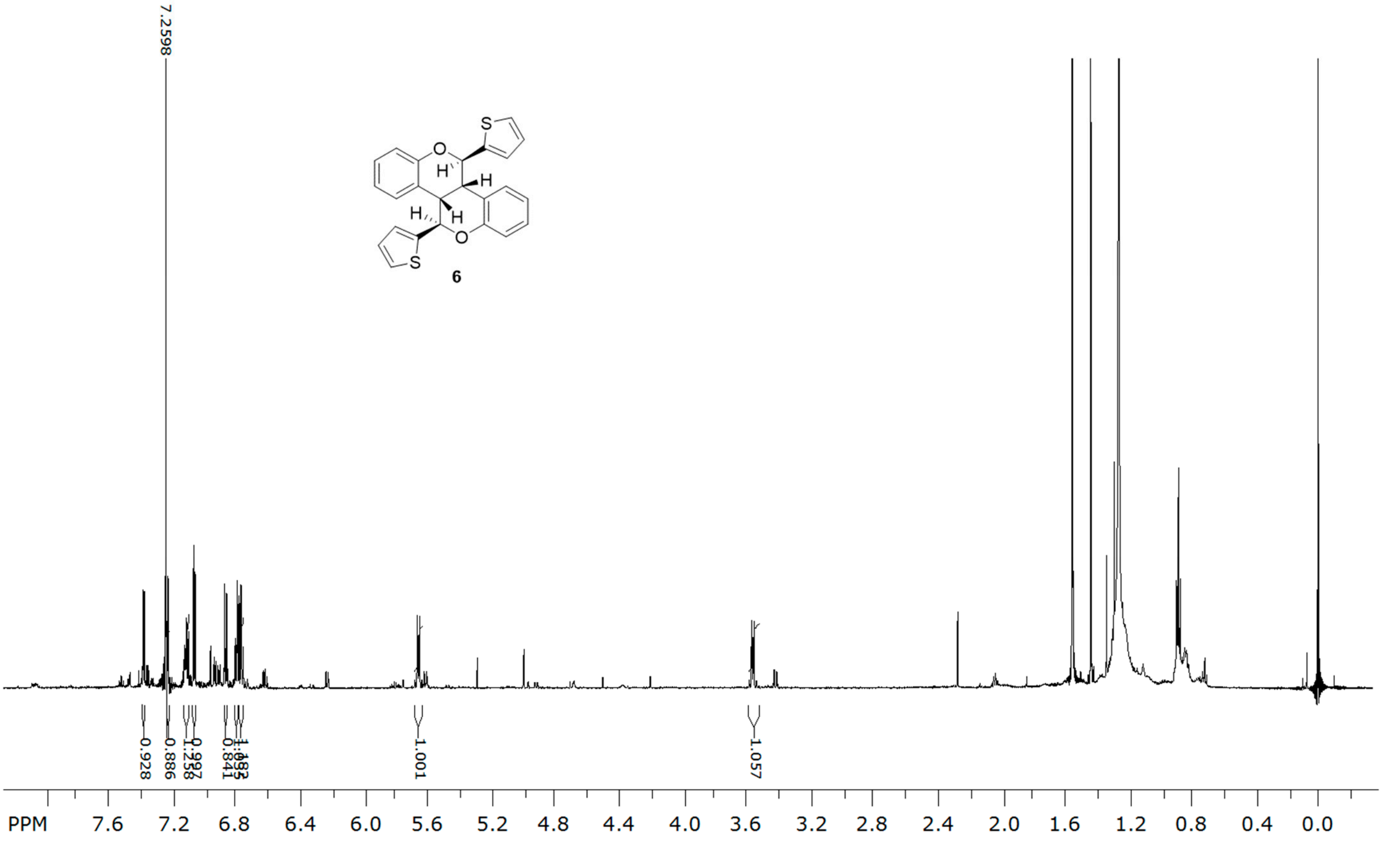

Figure S19. ^{1}H NMR ($CDCl_3$) spectrum of **6**.

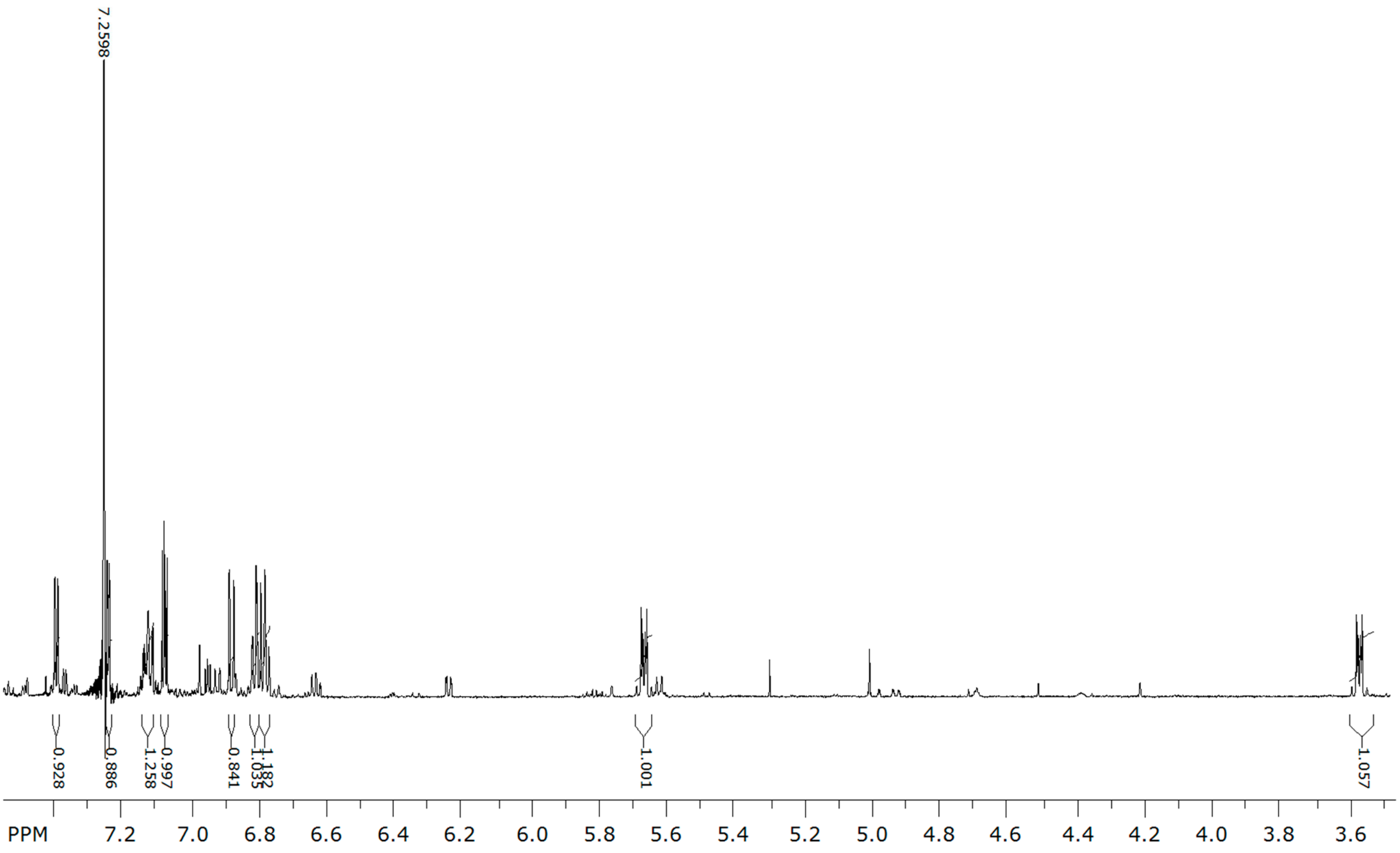

Figure S20. ^{1}H NMR ($CDCl_3$) spectrum of aromatic part of **6**.

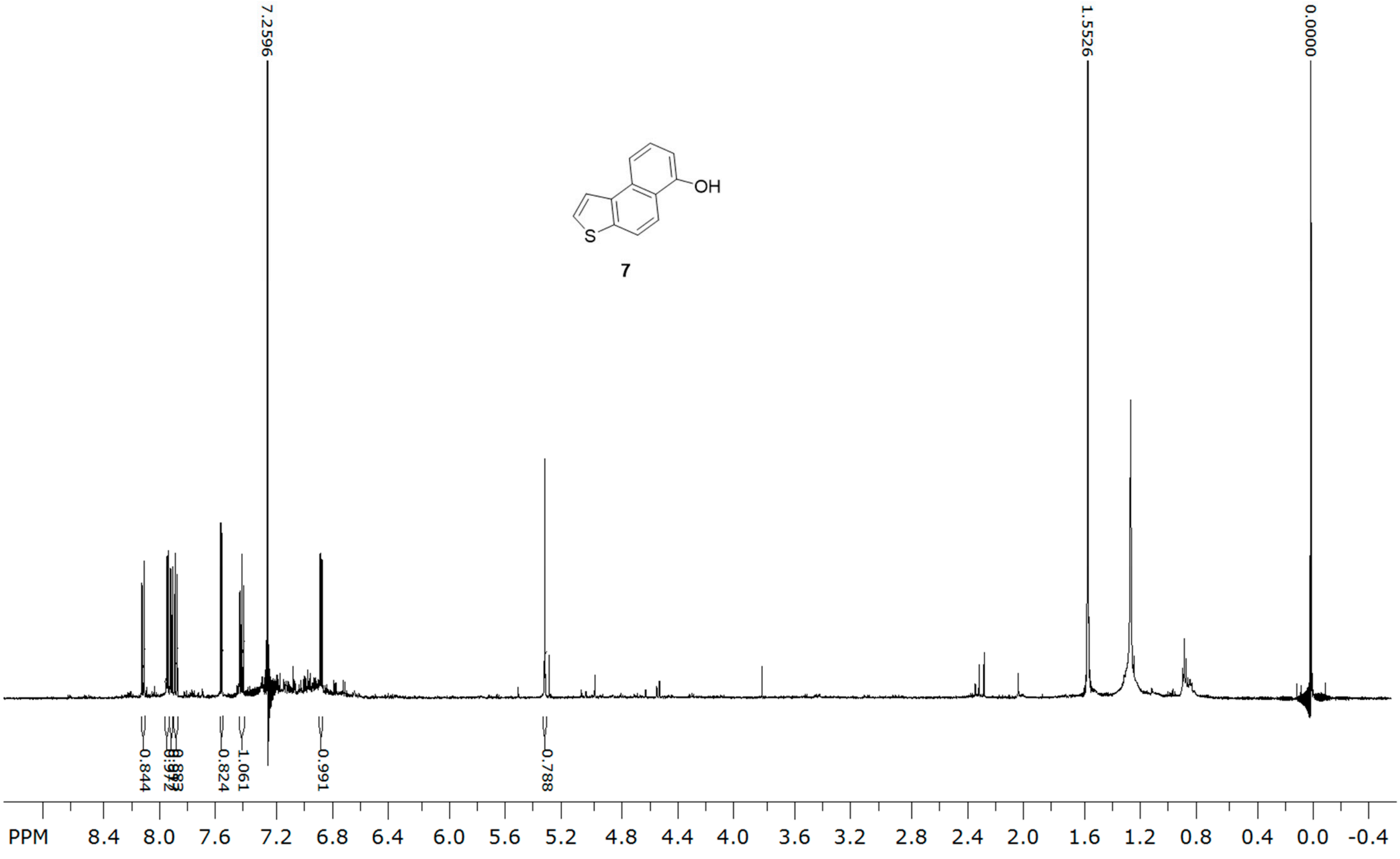

Figure S21. ^{1}H NMR ($CDCl_3$) spectrum of **7**.

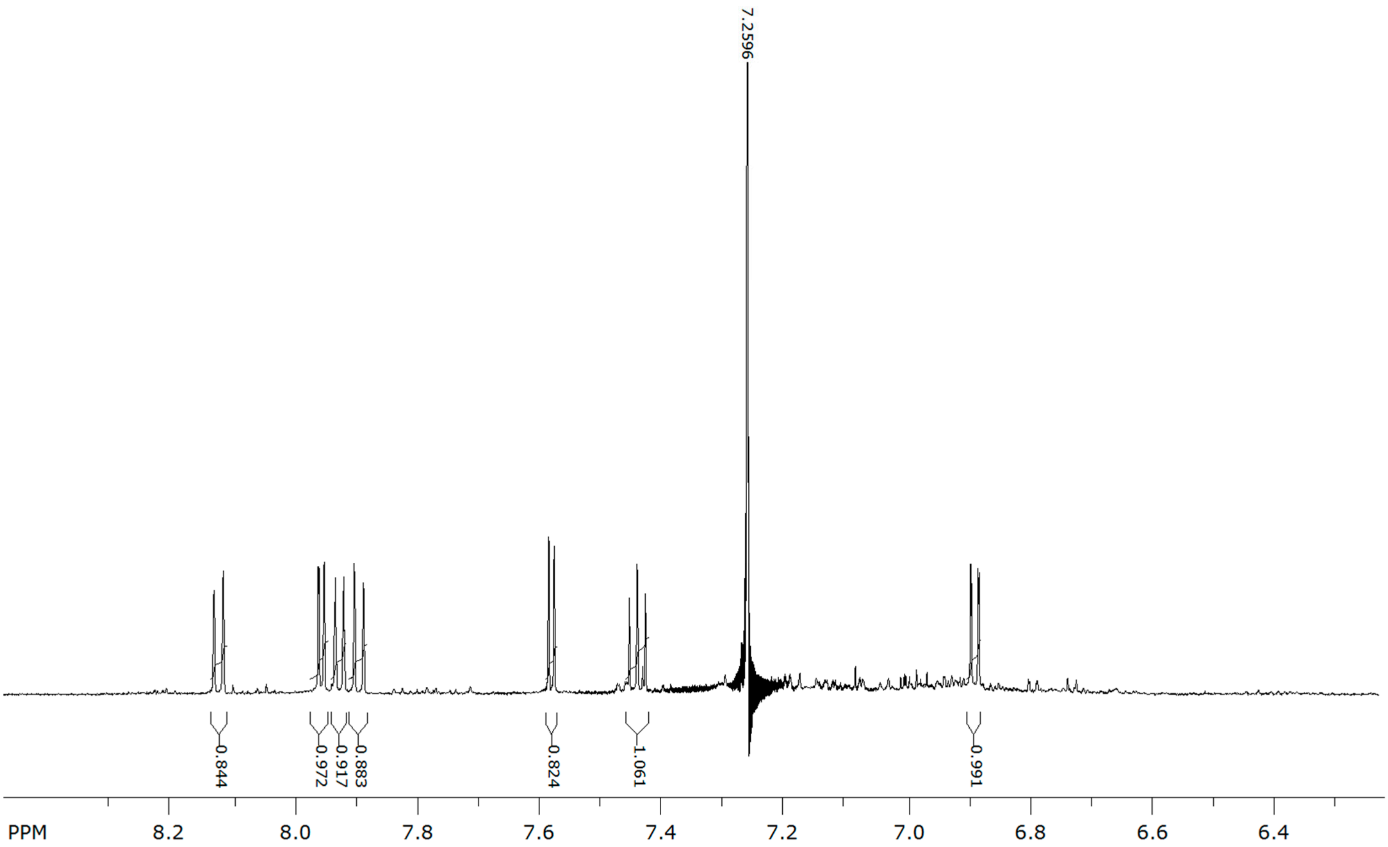

Figure S22. ^{1}H NMR ($CDCl_3$) spectrum of aromatic part of **7**.

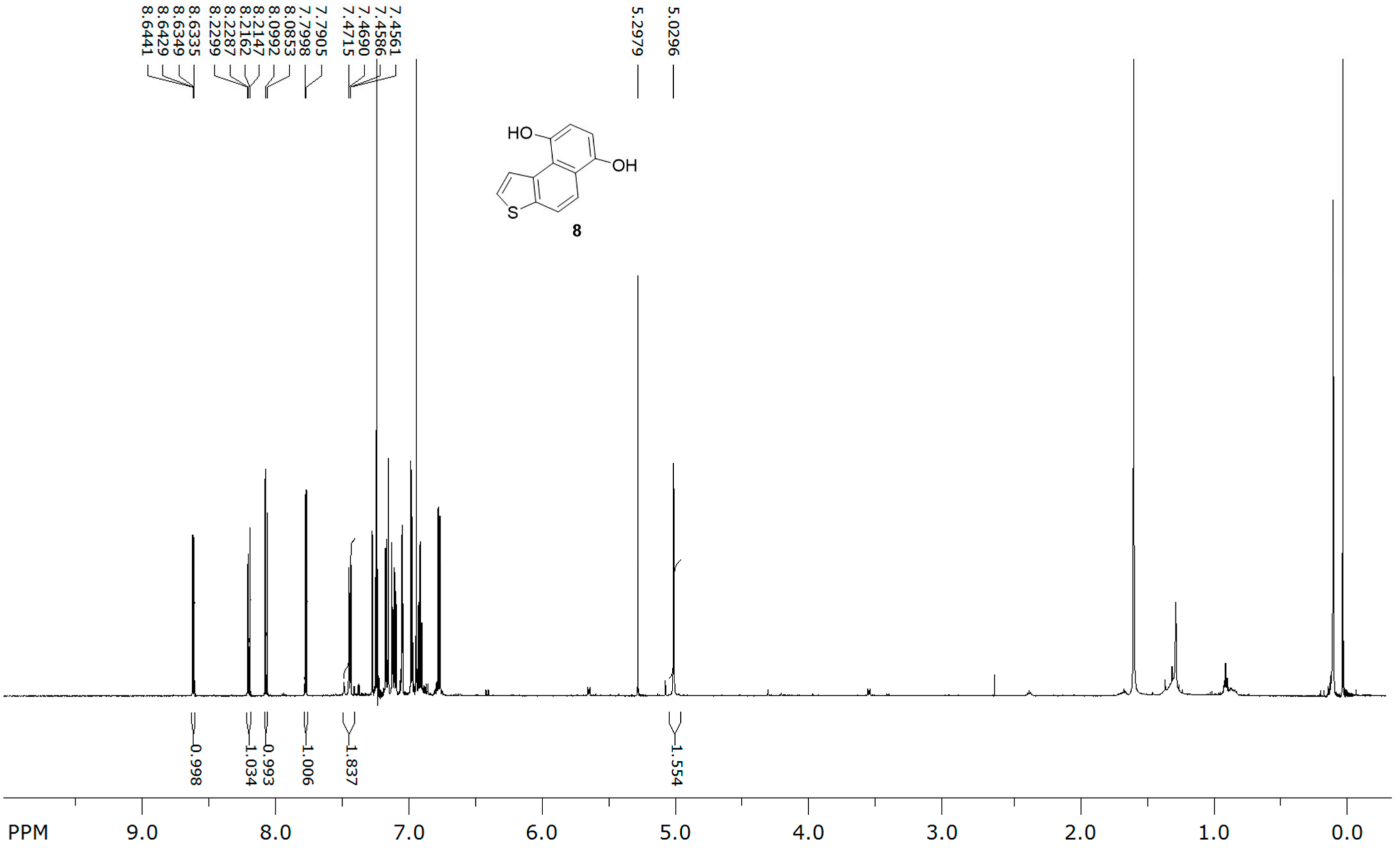

Figure S23. ^{1}H NMR ($CDCl_3$) spectrum of **8**.

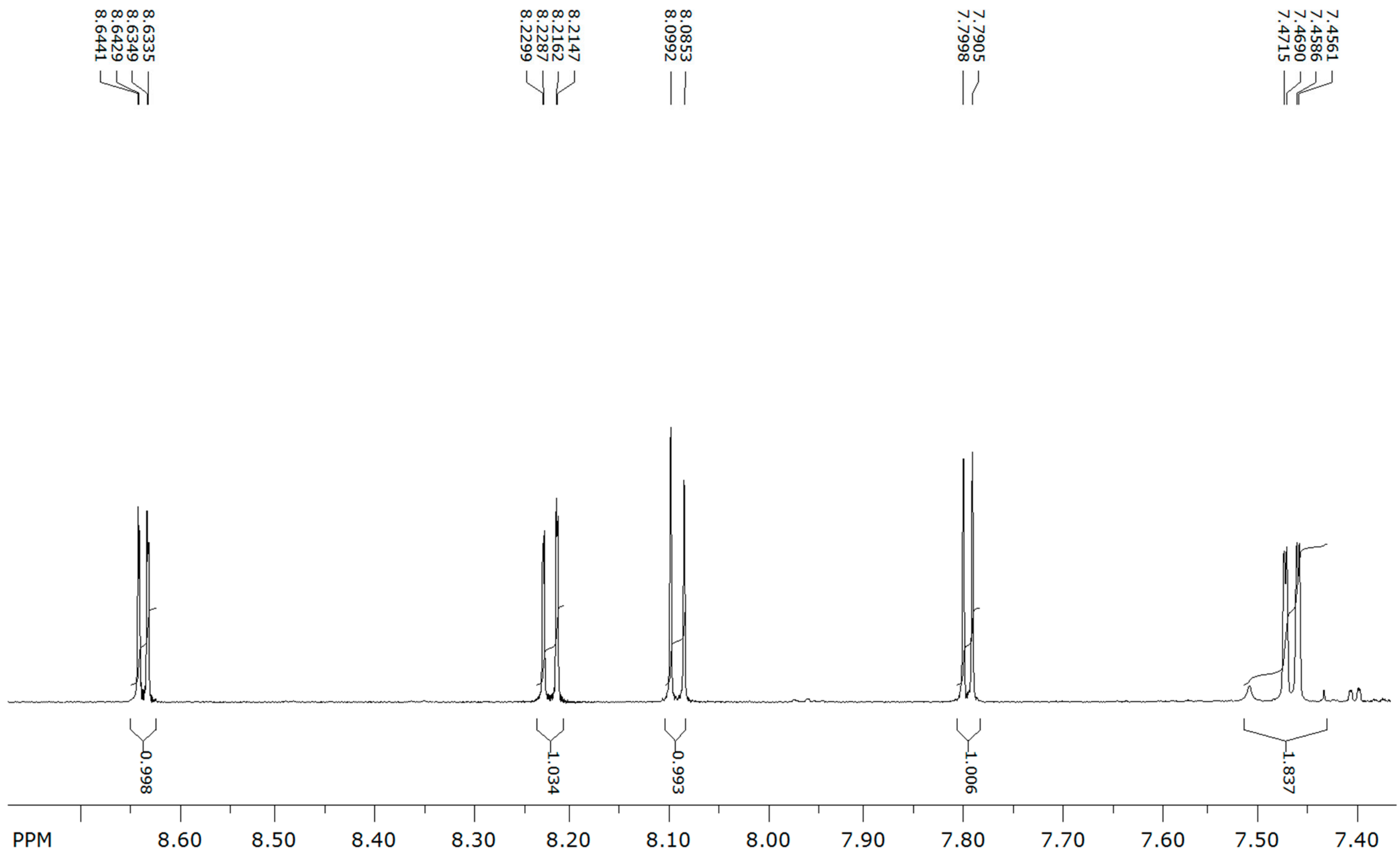

Figure S24. ^{1}H NMR ($CDCl_3$) spectrum of aromatic part of **8**.

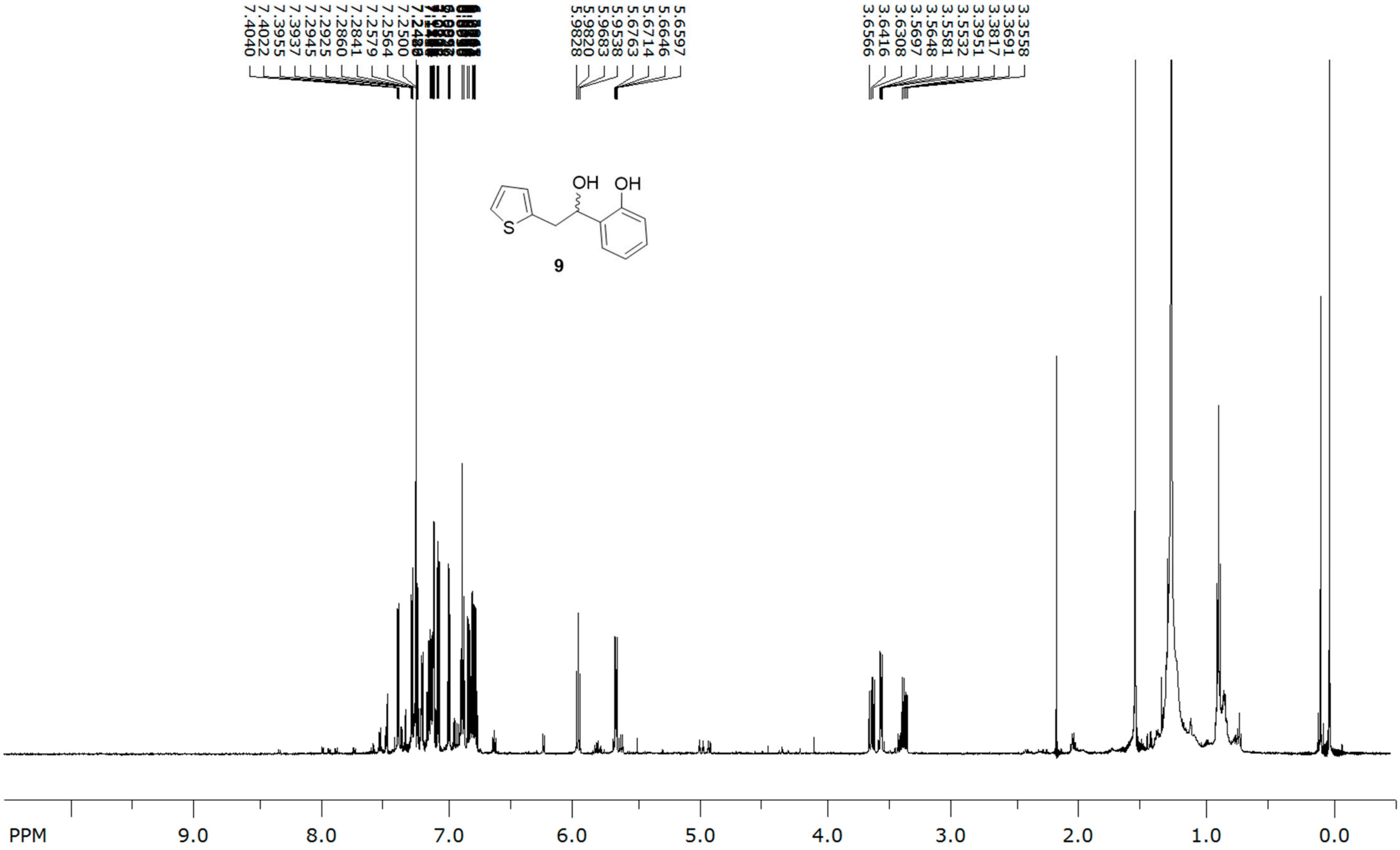

Figure S25. ^{1}H NMR ($CDCl_3$) spectrum of **9**.

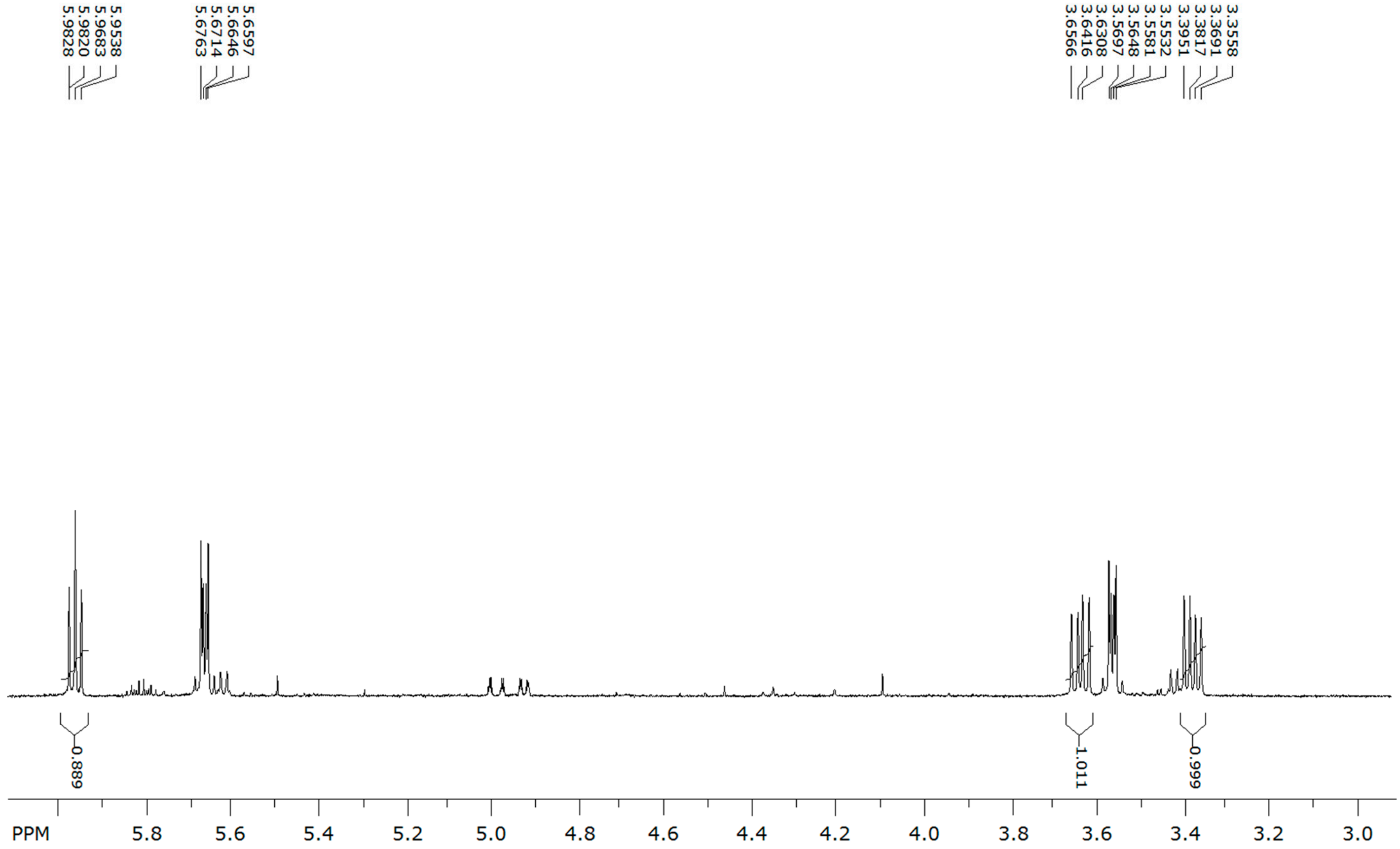

Figure S26. ^{1}H NMR ($CDCl_3$) spectrum of aromatic part of **9**.

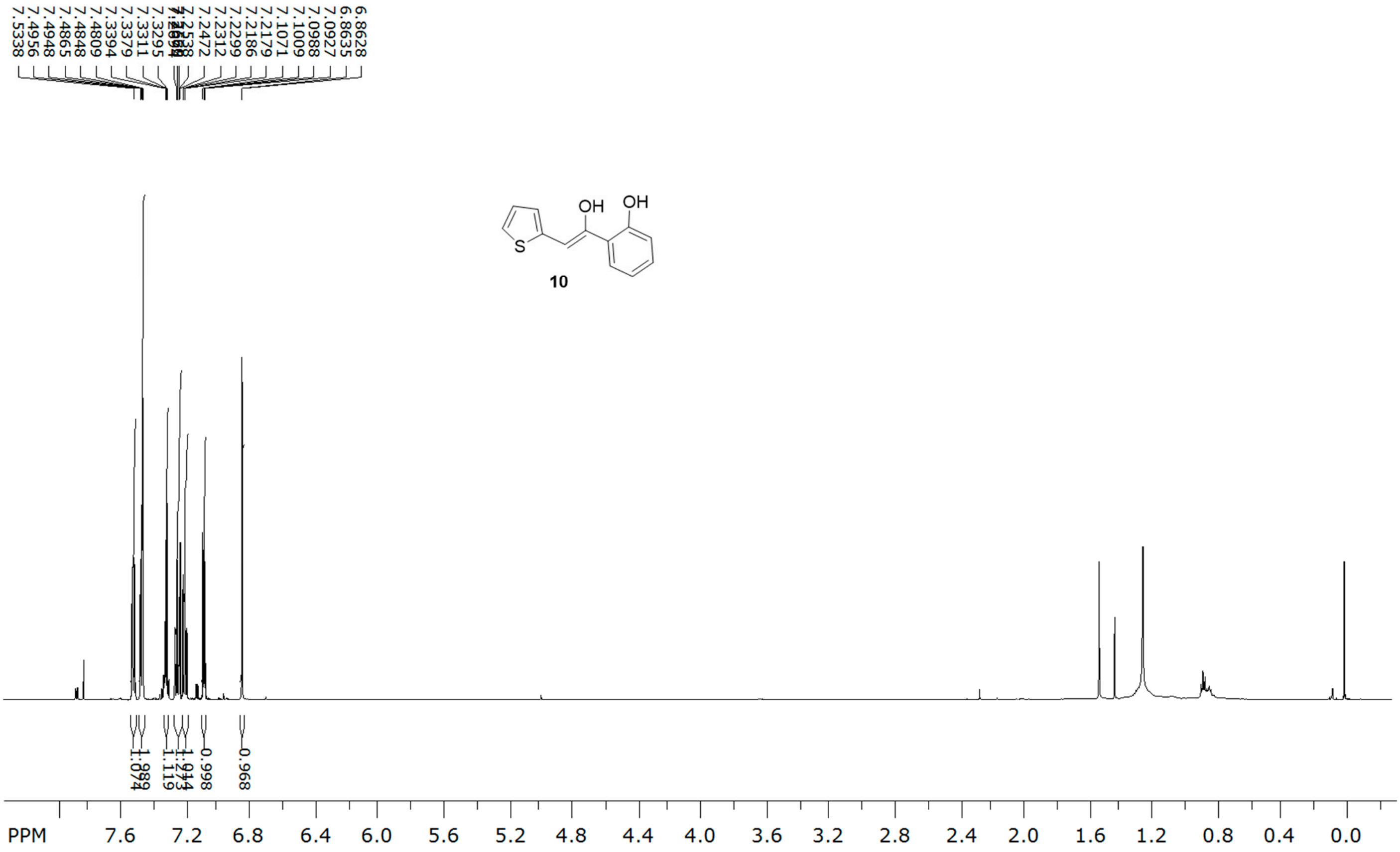

Figure S27. ^{1}H NMR ($CDCl_3$) spectrum of **10**.

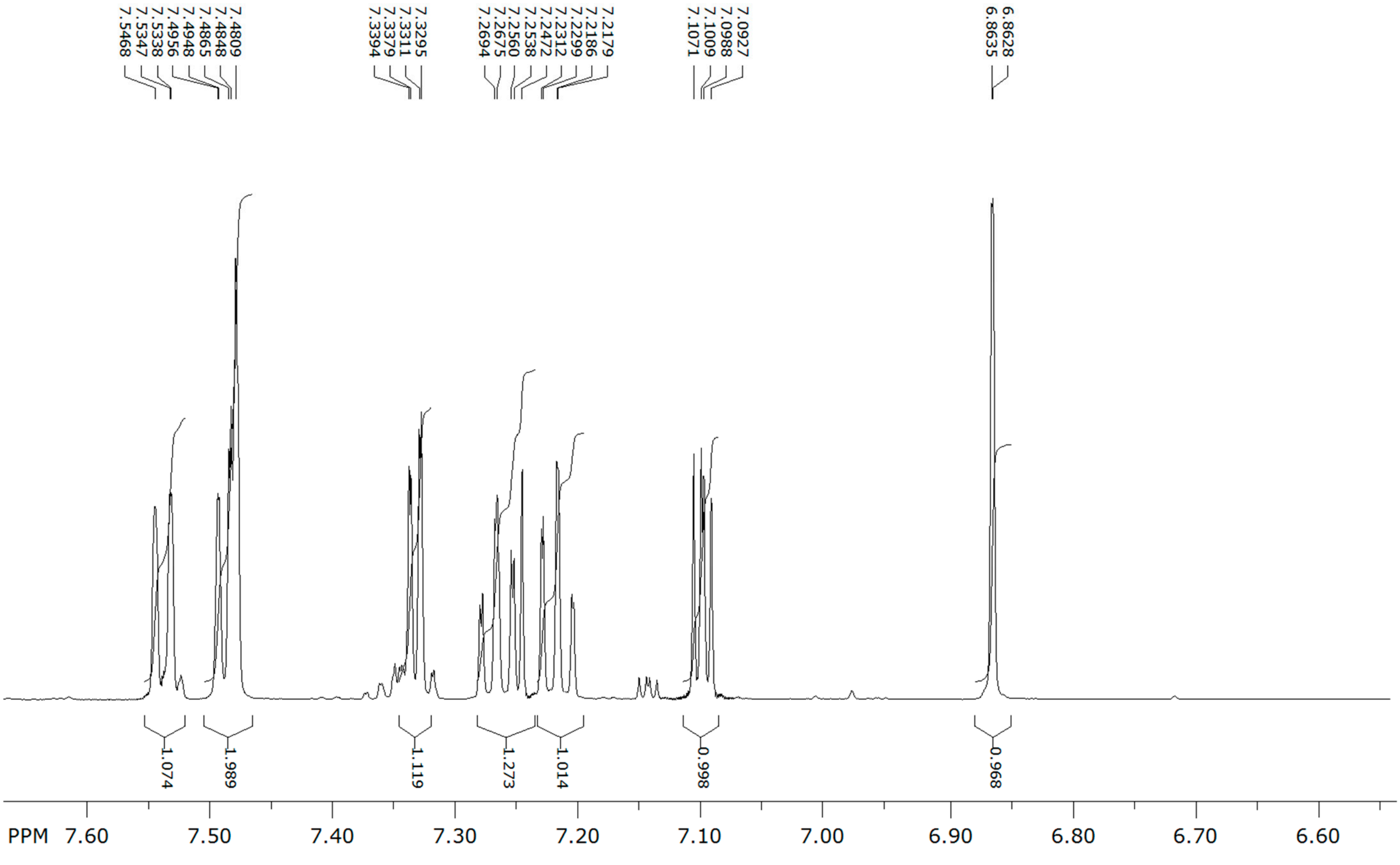

Figure S28. ^1H NMR ($CDCl_3$) spectrum of aromatic part of **10**.

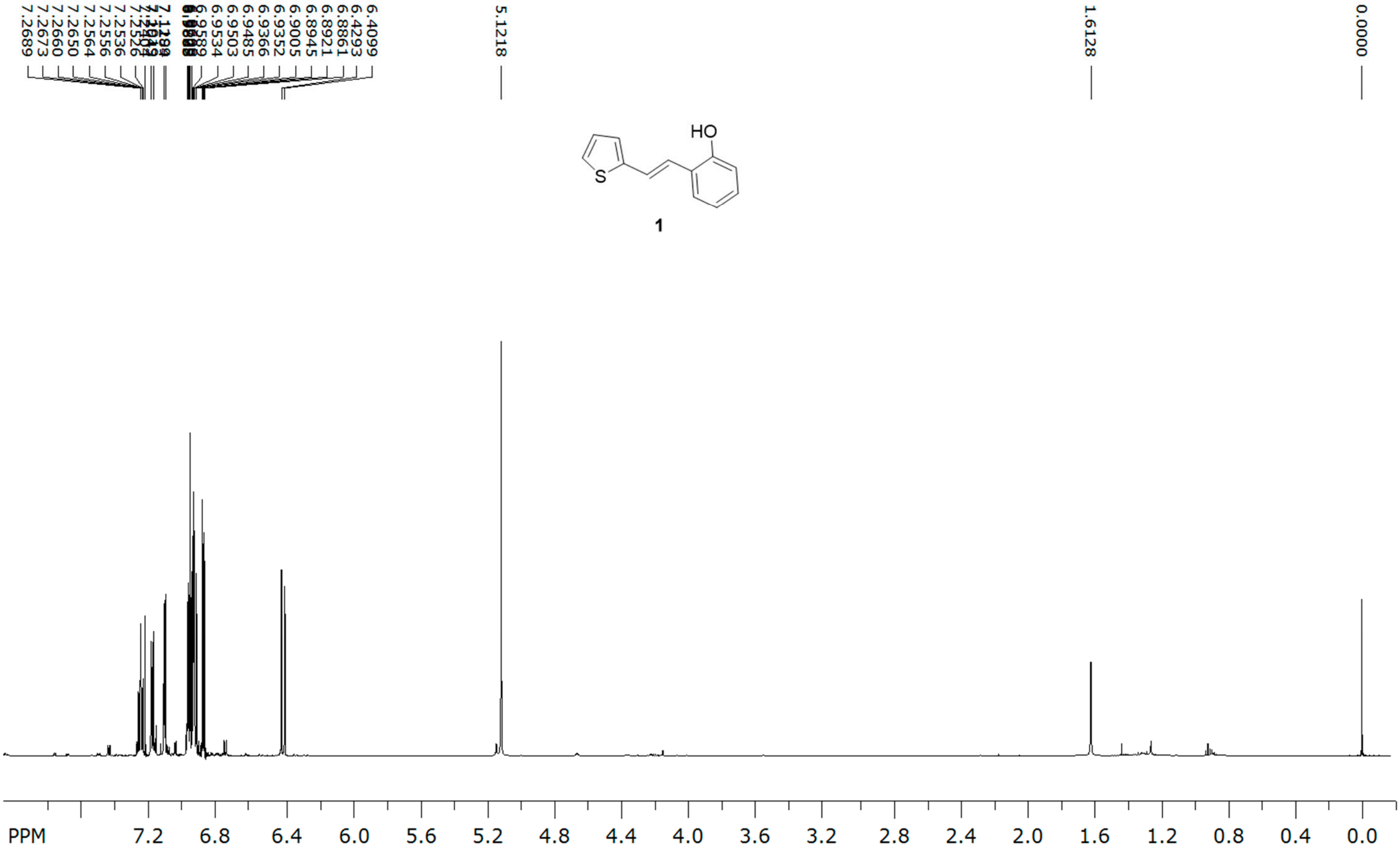

Figure S29. ^1H NMR ($CDCl_3$) spectrum of the photomixtures from **1** after 30′ of direct irradiation at 365 nm in a batch reactor.

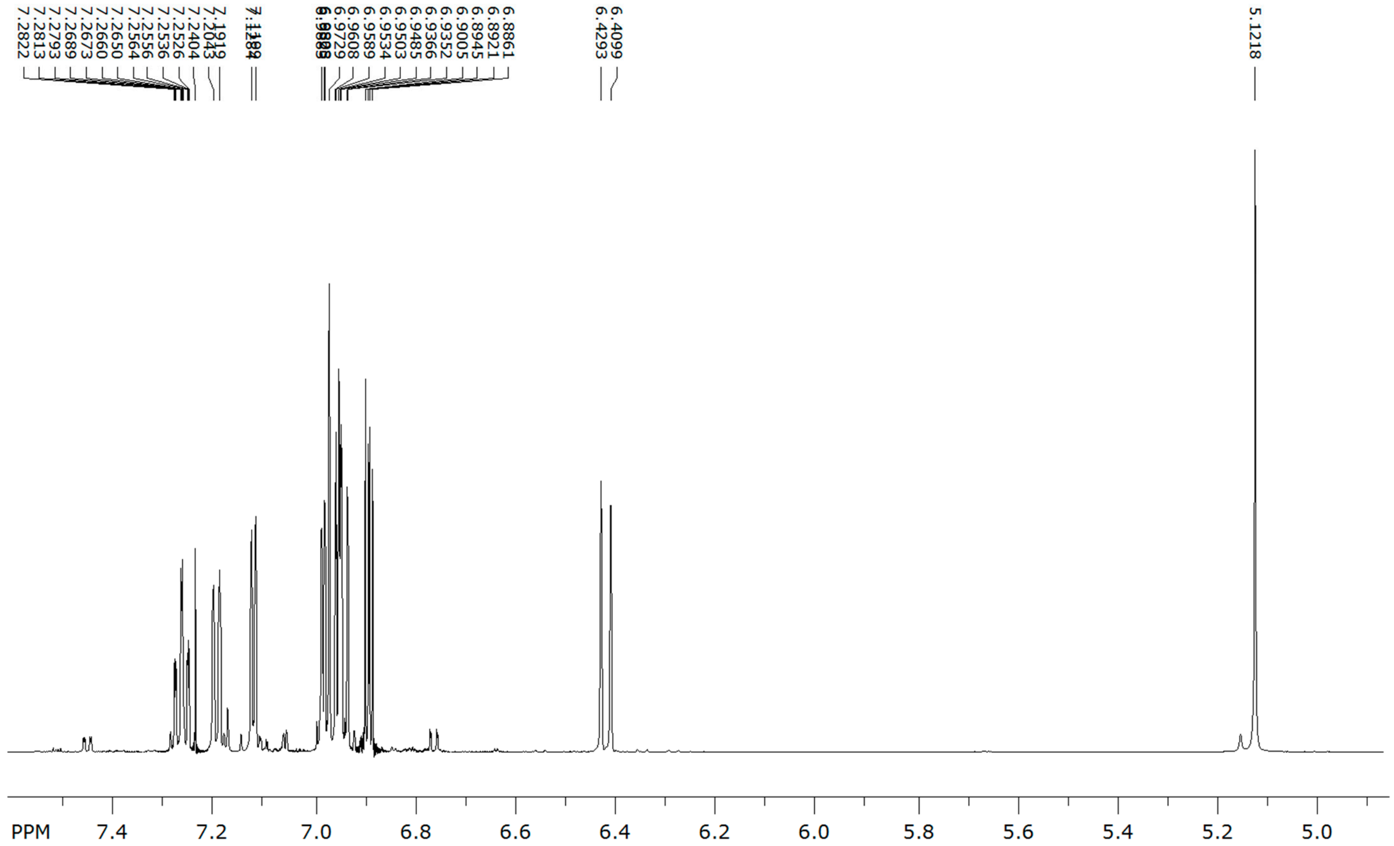

Figure S30. ^1H NMR ($CDCl_3$) spectrum of aromatic part of the photomixtures from **1** after 30′ of direct irradiation at 365 nm in a batch reactor.

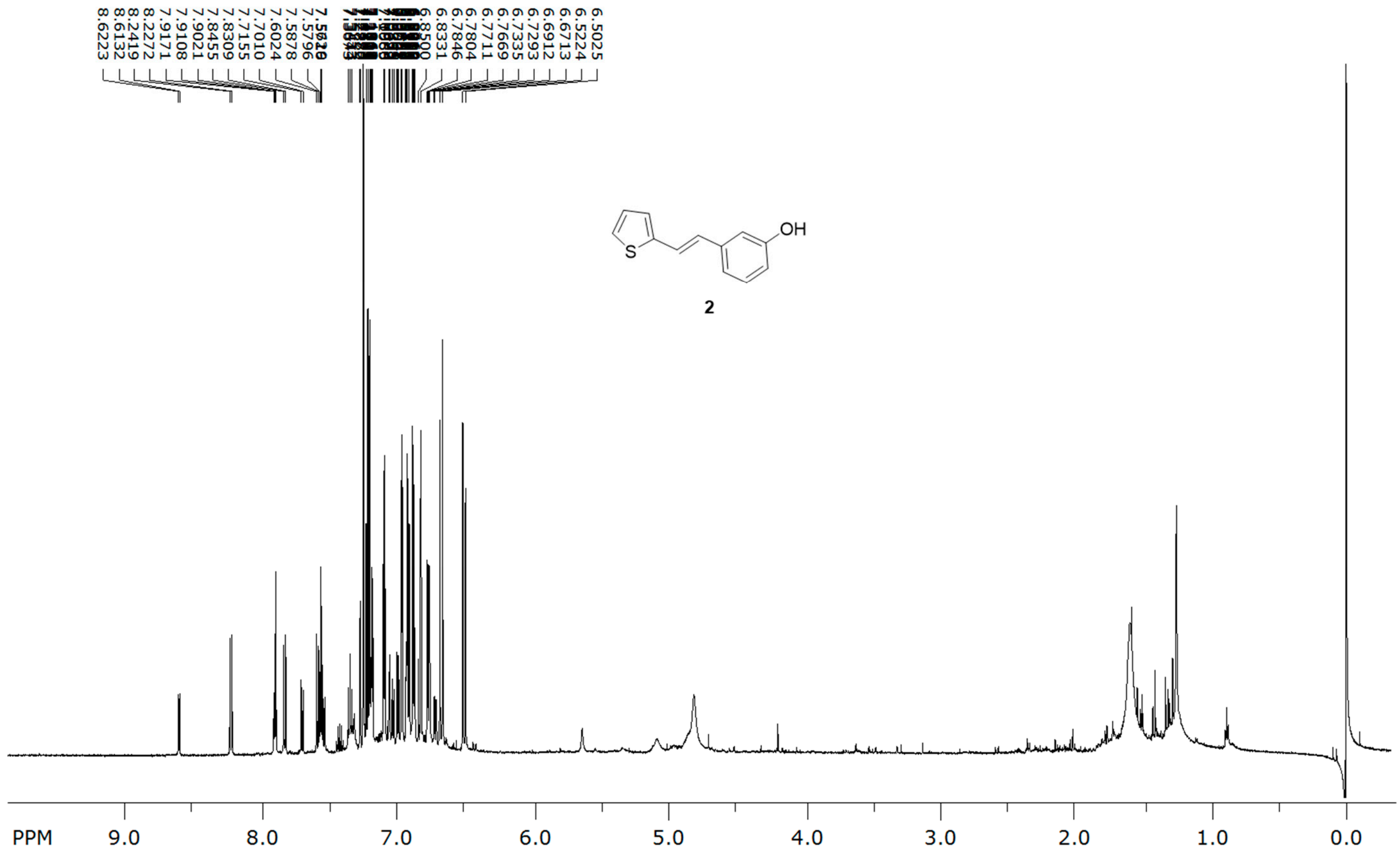

Figure S31. ^{1}H NMR ($CDCl_3$) spectrum of the photomixtures from **2** after 30′ of direct irradiation at 365 nm in a batch reactor.

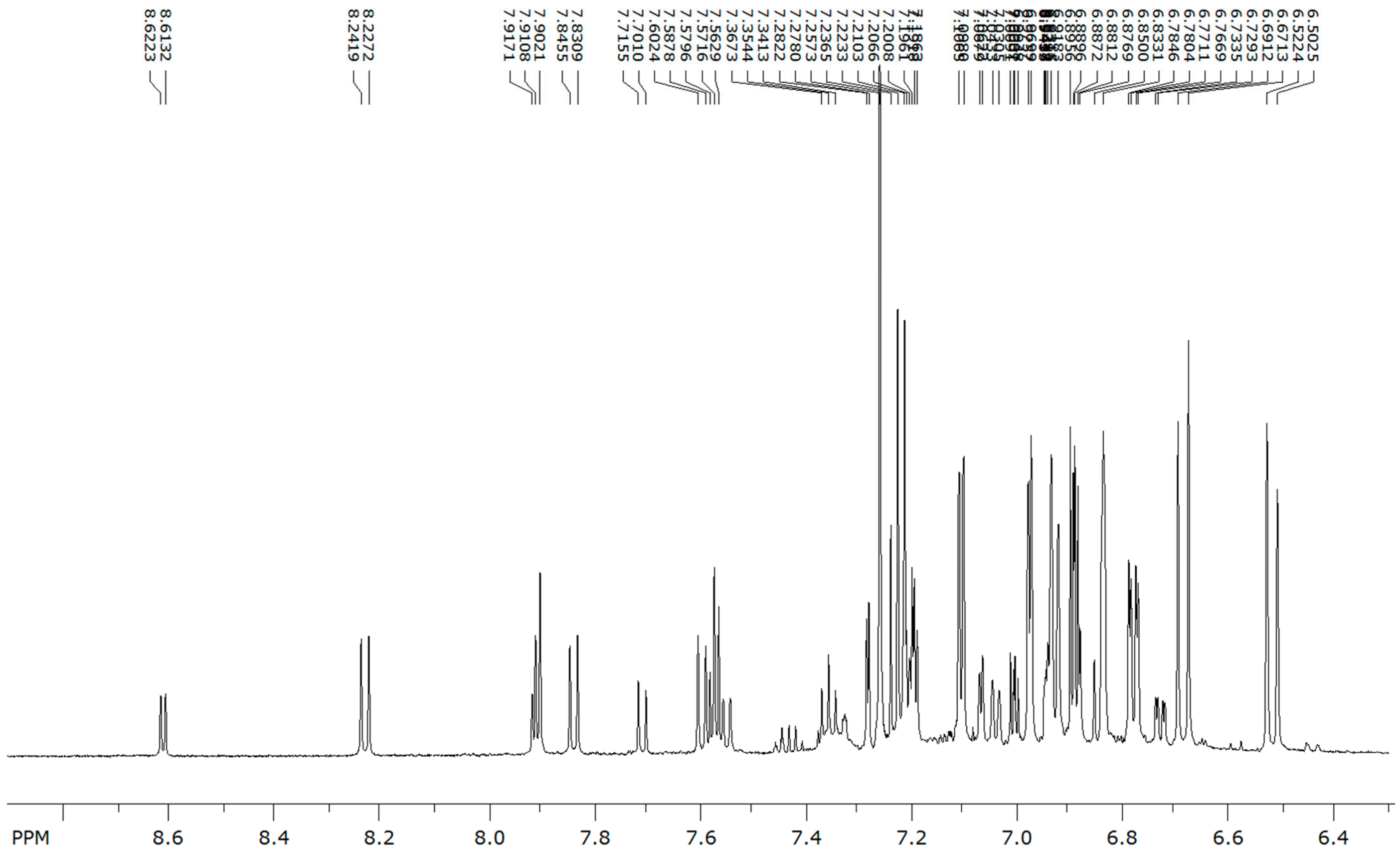

Figure S32. ^{1}H NMR ($CDCl_3$) spectrum of aromatic part of the photomixtures from **2** after 30′ of direct irradiation at 365 nm in a batch reactor.

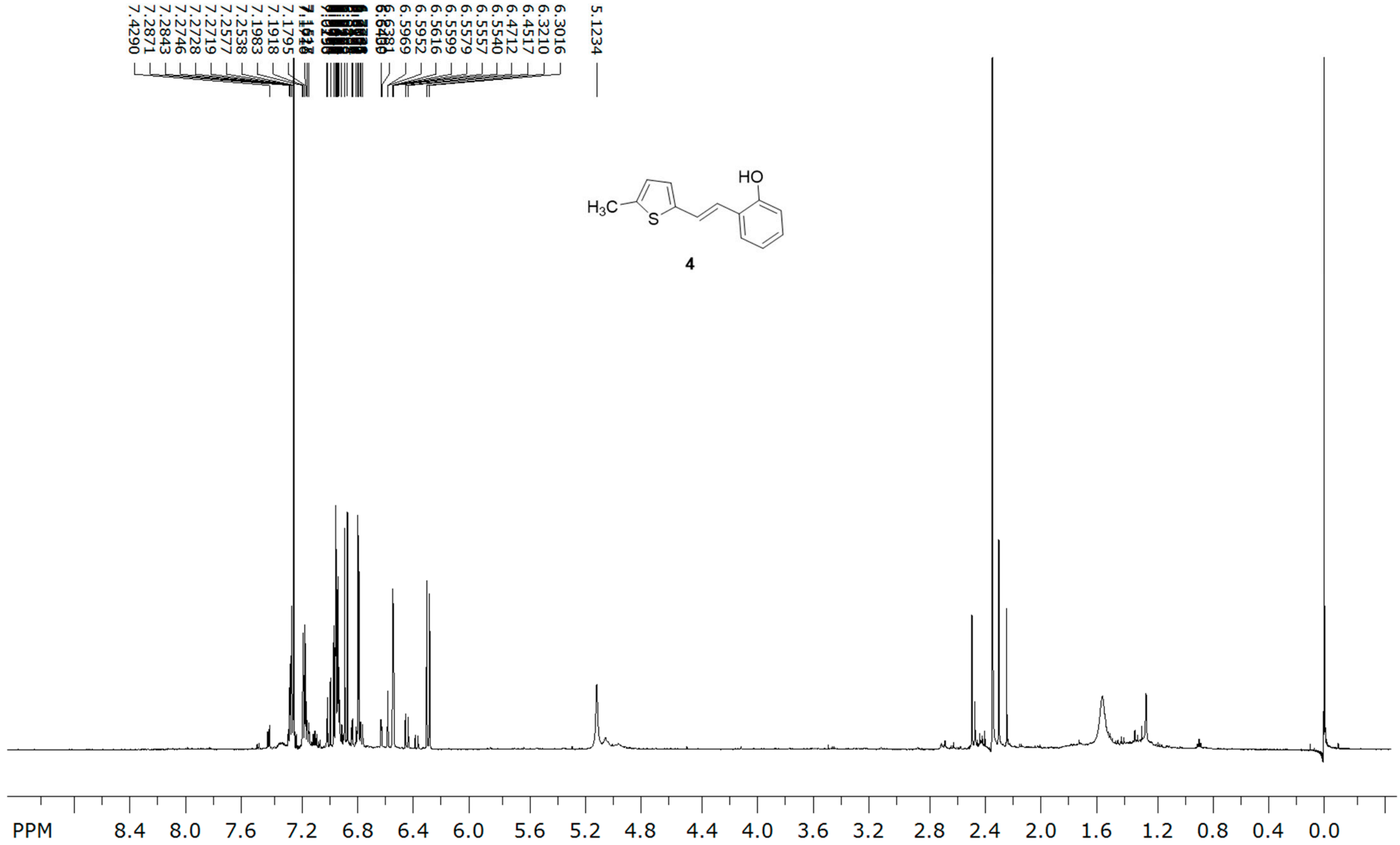

Figure S33. ^{1}H NMR ($CDCl_3$) spectrum of the photomixtures from **4** after 30′ of direct irradiation at 365 nm in a batch reactor.

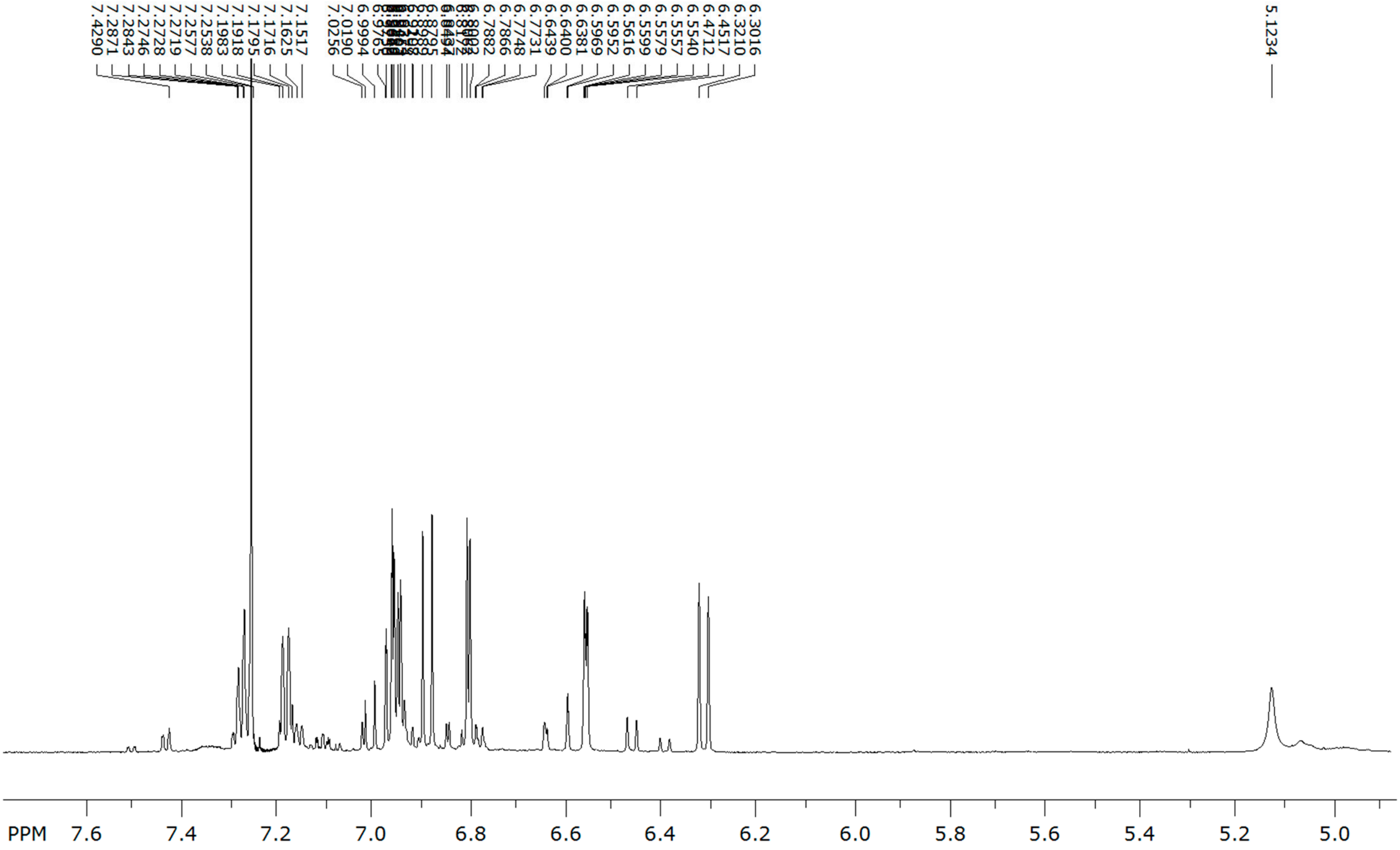

Figure S34. ^{1}H NMR ($CDCl_3$) spectrum of aromatic part of the photomixtures from **4** after 30′ of direct irradiation at 365 nm in a batch reactor.
